# Synthesis and in silico studies of certain benzo[*f*]quinoline-based heterocycles as antitumor agents

**DOI:** 10.1038/s41598-024-64785-z

**Published:** 2024-07-05

**Authors:** Eman A. E. El-Helw, Mahmoud Asran, Mohammad E. Azab, Maher H. Helal, Abdullah Y. A. Alzahrani, Sayed K. Ramadan

**Affiliations:** 1 Chemistry Department, Faculty of Science, Ain Shams University, Cairo, 11566 Egypt; 2 Chemistry Department, Faculty of Science, Helwan University, Ain-Helwan, Cairo, Egypt; 3 Chemistry Department, Faculty of Science and Arts, King Khalid University, Mohail Assir, Abha, Saudi Arabia

**Keywords:** Benzo[*f*]quinoline, Antiproliferative, Docking, Chromene, Cyanoacetohydrazone, In silico studies, Cancer, Chemical biology, Chemistry

## Abstract

A series of benzoquinoline-employing heterocycles was synthesized by treating 3-chlorobenzo[*f*]quinoline-2-carbaldehyde with *N*-phenyl-3-methylpyrazolone, 4-aminoacetophenone, 1,2-diaminoethane, and 2-cyanoethanohydrazide. Also, pyridine, chromene, α,β-unsaturated nitrile, thiosemicarbazone, and 1,2-bis-aryl hydrazine derivatives were prepared from the cyanoethanohydrazone obtained. The DFT calculations and experiment outcomes were consistent. In vitro screening of their antiproliferative efficacy was examined against HCT116 and MCF7 cancer cell lines. The pyrazolone **2** and cyanoethanohydrazone **5** derivatives exhibited the most potency, which was demonstrated by their molecular docking towards the CDK-5 enzyme. The binding energies of compounds **2** and **5** were − 6.6320 kcal/mol (with RMSD of 0.9477 Å) and − 6.5696 kcal/mol (with RMSD of 1.4889 Å), respectively, which were near to that of co-crystallized ligand (EFP). This implies a notably strong binding affinity towards the CDK-5 enzyme. Thus, pyrazolone derivative **2** would be considered a promising candidate for further optimization to develop new chemotherapeutic agents. In addition, the ADME (absorption, distribution, metabolism, and excretion) analyses displayed its desirable drug-likeness and oral bioavailability properties.

## Introduction

Designing and synthesizing scaffolds with biological characteristics is one of the primary goals of organic and medicinal chemistry. Heterocycles, including nitrogen, are targeted compounds in synthetic and medicinal chemistry because they serve as a critical scaffold in a range of physiologically active chemicals^1–10^. Benzoquinoline scaffolds are among the more abundant aza-arenes present in the environment^11–13^. Thus, benzoquinoline compounds exhibit a diverse set of biological properties and act as templates for the synthesis of numerous medications^14–17^. Benzoquinolines are known mutagens in *Salmonella typhimurium*^18^, and are also less toxic than quinolines^19^.

Furthermore, the biological significances of pyrazoles^20–22^, imidazolines^23,24^, and chromenes^25–27^ are well established. As a result, and in continuation to our work^28–33^, it has been of remarkable interest to design and synthesize various benzo[*f*]quinoline-carrying heterocycles to evaluate their antiproliferative properties. Computational chemical, molecular docking, and in silico studies were performed to support the findings.

### Rationale and design

One of the most important targets for the design of new anticancer drugs is DNA, through which drugs can change their configuration preventing replication and transcription leading to cancer cell growth inhibition. Intercalation is the favored binding mode of almost-flat polyaromatic ligands of larger surface area and applicable steric properties. The nature and size of intercalating chromophores are important parameters that govern the binding modes. Noteworthy, many anticancer drugs in clinical utility (doxorubicin, anthracyclines, mitoxantrone) interact with DNA through intercalation^34,35^.

In a statement, most intercalating agents may be either positively charged or include basic groups protonating under physiological conditions^36^. Recently, much consideration has been promoted to design and synthesize novel and efficient DNA-targeted anticancer agents encompassing quinoline scaffold^35,37^.

**Figure 1:**
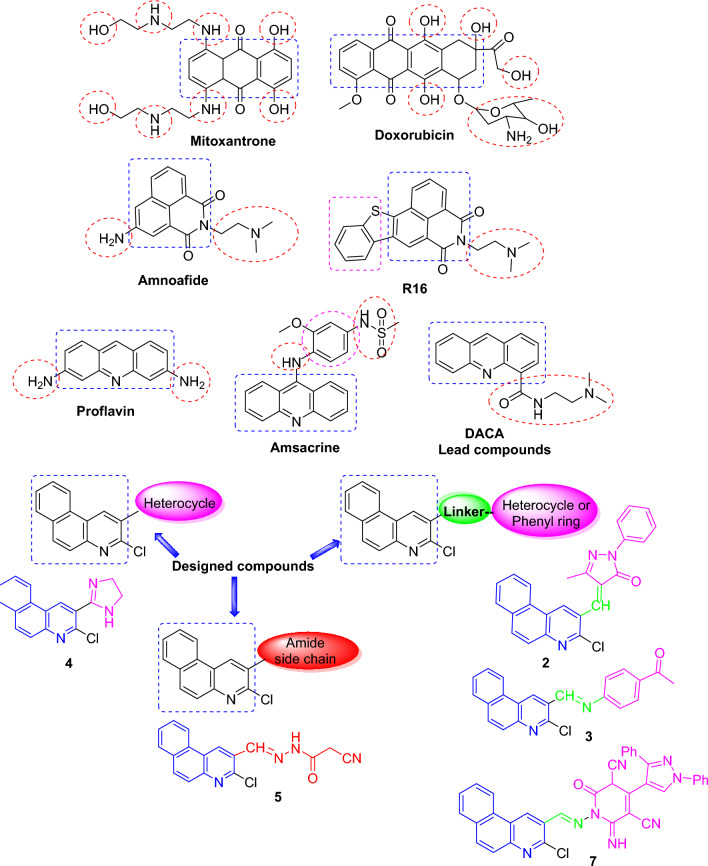
Some DNA-intercalating agents and designed substrates.

Thus, this work reports the molecular design and synthesis of a series of new benzo[*f*]quinolines (planar polyaromatic ligands) encompassing either side chain, phenyl, or heterocyclic scaffold at position-3 of quinoline core like some known DNA-intercalating agents. The rationale for the design of these substrates can be shown in Fig. 1. For example, amonafide-bearing aminoalkyl side chain showed significant cytotoxic potency via intercalation and topoisomerase II inhibition^38^. R16 exhibits potent anticancer properties and anti-multidrug resistant (MDR) capability^39^. Compound **2**, a benzo[*f*]quinoline with a pyrazolone scaffold, displayed the best intercalative properties among synthesized quinolines.

## Results and discussion

### Synthesis

**Scheme 1:**
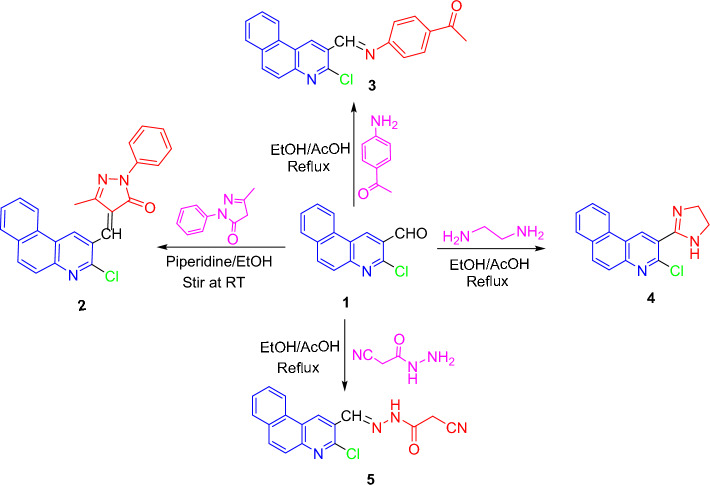
Condensation of 3-chlorobenzoquinoline-2-carbaldehyde **1** with some nucleophiles.

In this work, 3-chlorobenzo[*f*]quinoline-2-carbaldehyde **(1)**^40^ was utilized for the creation of various heterocycles based on the benzo[*f*]quinoline framework. Thus, treating a basic ethanolic solution of aldehyde **1** with 5-methyl-2-phenyl-2,4-dihydropyrazol-3-one furnished the condensation product, pyrazolone **2** (Scheme 1). IR of **2** disclosed pyrazolone’s carbonyl absorption band. Its ^1^H NMR indicated a singlet signal for methyl protons. Besides other peaks, its mass spectrum displayed isotopic peak (M + 2) at *m/z* 399.68 (7%) and molecular ion peak (M^+.^) at *m/z* 397.08 (21%).

On the other side, Schiff base **3**, imidazoline **4**, and cyanoethanohydrazone **5** derivatives were produced by the reactions of aldehyde **1** reacted with 4-acetylaniline, 1,2-diaminoethane, and 2-cyanoethanohydrazide, respectively (Scheme 1). Theres was no aldehydic carbonyl absorption in IR of **3–5**. A singlet signal for methyl protons of acetyl group occurred in ^1^H NMR of **3**. Also, two triplet signals for CH_2_-CH_2_ protons of imidazoline moiety appeared in ^1^H NMR of **4**. A possible pathway for the formation of imidazoline **4** was first condensation between the aldehydic carbonyl group of **1** with one NH_2_ group of 1,2-diaminoethane to remove water molecule pursued by 5-endo-trig cyclization by nucleophilic addition of second NH_2_ group on CH=N moiety, then dehydrogenation.

Absorption bands for NH, C≡N, C=O, and C=N moieties were observed at ν 3255, 2265, 1700, and 1602 cm^−1^, respectively, in IR of hydrazone **5**. Further, its ^1^H NMR implied its existence as a mixture of *anti*- and *syn*-isomers in a ratio of 80.3–19.7%, respectively. As a result, for *anti*-isomer, it offered a singlet signal of CH_2_ protons at 4.37 ppm, a singlet signal for methine proton (CH=N) at δ 8.46 ppm; while for *syn*-isomer, the former signal was at δ 3.89 ppm, and the later signal was at δ 8.64 ppm. Moreover, its mass chart proved its molecular ion peak at *m/z* = 322.35 (52%), in addition to the isotopic (M + 2) peak at *m/z* = 324.50 (20%) (cf. Experimental).

**Scheme 2:**
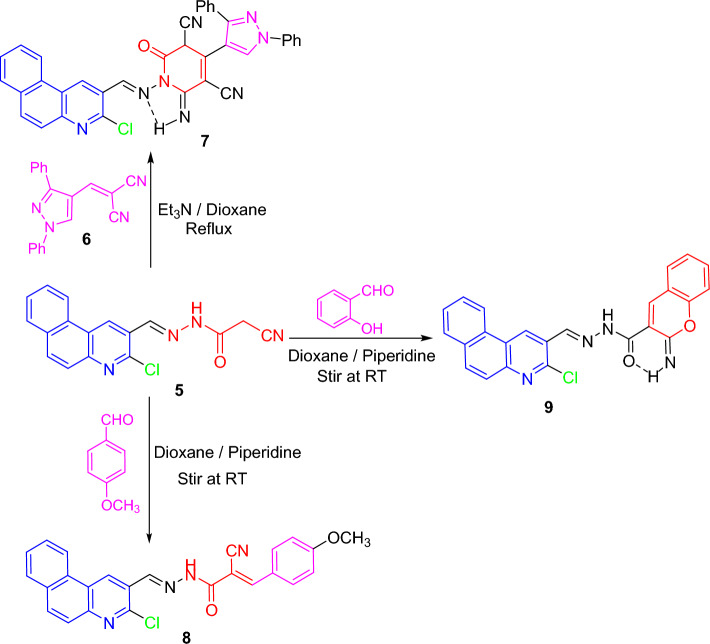
Interaction of cyanoacetohydrazone **5** with some carbon electrophiles.

**Scheme 3:**
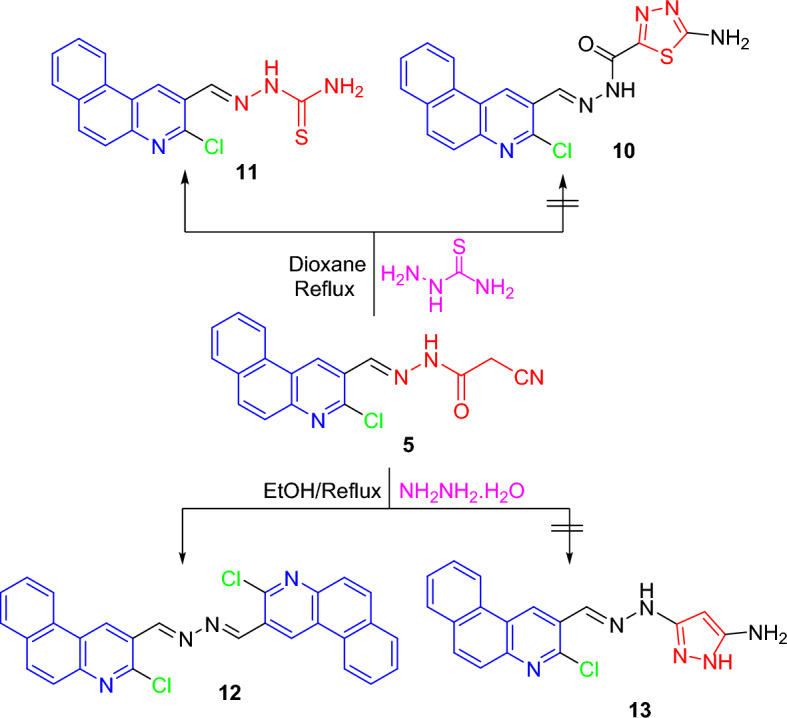
Reaction of cyanoethanohydrazone **5** with thiosemicarbazide and hydrazine.

Noteworthy, the benzoquinoline-cyanoethanohydrazone **5** was feasible to be a highly reactive substrate, as the active methylene group can contribute to condensation reactions, in addition to carbonyl and cyano moieties are appropriately situated to build diverse heterocyclic skeletons. Certainly, cyanoethanohydrazone **5** was utilized as synthons for various interesting benzo[*f*]quinolines via reaction with certain carbon electrophiles and nitrogen nucleophiles (cf. Schemes 2, 3). First, the pyridine candidate **7** was furnished upon conducting **5** *with 2-((1,3-diphenylpyrazol-4-yl)methylene)malononitrile* **(6)** under basic medium (Scheme 2). In IR of **7**, absorption bands appeared for NH, CN, and C=O. Further in its ^1^H NMR, singlet signals for imino proton (NH) in the downfield region and C3-H of pyridine moiety occurred.

Knoevenagel reaction of **5** with 4-methoxybenzaldehyde in dioxane and piperidine at an ambient temperature provided α,β-unsaturated nitrile **8**. In its IR, a lower absorption band for conjugated nitrile was indicated. Also, its ^1^H NMR lacked the methylene singlet, and offered its being as a mixture of *E*- and *Z*-isomers in a ratio of 80:20%, respectively.

Besides under similar conditions, cyclo-condensation of **5** with 2-hydroxybenzaldehyde constructed 2-iminochromene **9**. Its IR was devoid of nitrile absorption but offered absorption bands for NH and C=O groups. Further, its ^1^H NMR provided two NH singlet signals. Furthermore, the molecular ion peak at *m/z* 426.78 (100%) and isotopic peak (M + 2) at *m/z* 428.94 (35%) occurred in its mass chart (cf. Experimental).

The interaction of cyanoethanohydrazone **5** with thiosemicarbazide and hydrazine was inspected (Scheme 3). Consequently, treating **5** with thiosemicarbazide in 1,4-dioxane did not produce thiadiazole **10** but formed thiosemicarbazone **11**^13^. Likewise, hydrazinolysis of **5** with hydrazine in ethyl alcohol provided 1,2-bis-hydrazine candidate **12**^13^ instead of pyrazole product **13**.

### Density functional theory (DFT) simulation

DFT simulation was applied to optimize the molecular configurations of the produced substances, identify both electrophilic and nucleophilic centers, and infer the reactions path^2,26^. The molecular structures of produced substances are superior to be designed in a stable configuration. Hence, benzo[*f*]quinoline derivatives' geometry was progressively optimized, and their energy was incessantly diminished until the molecule's energy fluctuations were minimized. The electrophilic-attacking centers are depicted by the HOMO areas of maximal electron density, whereas the nucleophilic-attacking sites are implied by the LUMO regions. The high *E*_HOMO_ values are prospective to signify a molecule's strong propensity to offer electrons. The optimized, HOMO, and LUMO configurations of substrates **2–9** were portrayed applying ChemBio3D Ultra 14.0 and depicted in Supplementary Fig. 1.

To establish how 3-chlorobenzo[*f*]quinoline-2-carbaldehyde **(1)** reacted with certain reagents to produce substrates **2–9**, a DFT simulation has been operated to calculate quantum chemical properties including global hardness, softness, chemical potential, global electrophilicity index, nucleophilicity index, ionization potential, electron affinity, and electronegativity (cf. Table 1). Analytical and spectral data proved the assigned structures. Due to the low energy needed to remove an electron from the last occupied orbital, compounds with low energy gap values (Δ*E* = *E*_LUMO_ − *E*_HOMO_) will display potent inhibition efficiency^26,32,41^. Quantum chemical parameters calculations were in decent agreement with the antiproliferative efficacy (Table 1)^26^. The findings pointed out that the energy gap values (ΔE) track the order: **3 < 2 < **doxorubicin < **9 < 8 < 7 < 5 < 4**. Substrates having low ΔE values are commonly implied to as soft substances, which are more reactive toward radical surface interactions; being efficient in offering electrons clearly to the hole surface.

**Table 1:** Energy level distribution of frontier orbitals and global reactivity indices.

Compd	*E*	*E*_HOMO_ (eV)	*E*_LUMO_ (eV)	Δ*E* (eV)	*µ* (Debye)	*η *(eV)	*ς *(eV^−1^)	*μ*_*o*_ (eV)	*ω *(eV)	*n *(eV^−1^)	*Ip *(eV)	*EA *(eV)	*x *(eV)
**2**	37.118	− 7.555	− 5.916	1.639	− 2.692	0.819	1.221	− 6.735	27.69	0.036	7.555	5.916	6.735
**3**	24.339	− 7.543	− 5.938	**1.605**	− 0.099	0.802	**1.247**	− 6.740	28.32	0.035	7.543	5.938	6.740
	16.837	− 7.549	− 3.545	4.004	− 0.292	2.002	0.499	− 5.547	7.685	0.130	7.549	3.545	5.547
**5**	6.644	− 7.631	− 3.813	3.818	− 1.568	1.909	0.524	− 5.722	8.575	0.117	7.631	3.813	5.722
**7**	59.777	− 7.374	− 3.833	3.541	7.125	1.770	0.565	− 5.603	8.868	0.113	7.374	3.833	5.603
**8**	24.197	− 7.407	− 4.333	3.074	− 0.581	1.537	0.651	− 5.870	11.21	0.089	7.407	4.333	5.870
**9**	20.240	− 7.644	− 5.098	2.546	− 0.894	1.273	0.785	− 6.371	15.94	0.063	7.644	5.098	6.371
**Dox.**	67.785	− 9.189	− 7.149	2.040	3.954	1.020	0.980	− 8.169	32.72	0.030	9.189	7.149	8.169

*Dox.* Doxorubicin, *E* Minimized Energy (kcal/mol), *µ* Dipole/dipole, *η *Global Hardness, *ς *Global Softness, *μ*_*o*_ Chemical Potential, *ω *Global Electrophilicity Index, *n* Nucleophilicity Index, *Ip* Ionization Potential, *EA* Electron Affinity, *x* Electronegativity.

Chemical softness values decrease in the order of **3, 2,** doxorubicin**, 9, 8, 7, 5**, and **4**, respectively, while the hardness values rise in the same order. The scavenging ability toward positive hole, tumor, radical, and oxygen removable was not only dependent on *E*_HOMO_ values but also, the electron distributions, number of heteroatoms, surface area, and lipophilicity should be measured^41–44^. The dipole moment (Debye) and softness (ϭ, eV^−1^) for most potent substances holding hydrophobic groups were agreed to an outstanding correlation between oxidation inhibition efficiencies. Correspondingly, substances of higher binding energy are of higher potency due to effective interaction between these substances and the receptors’ active sites.

**Figure 2:**
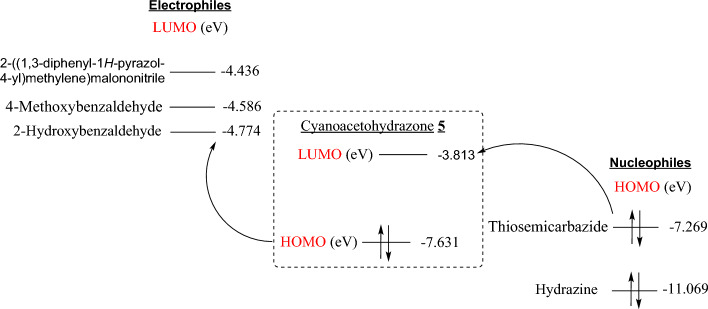
The *E*_LUMO_ of electrophiles and *E*_HOMO_ of nucleophiles toward *E*_HOMO_ and *E*_LUMO_ of cyanoethanohydrazone substrate **5**.

Otherwise, for reactions of carbon-centered electrophiles like 2-((1,3-diphenylpyrazol-4-yl)methylene)malononitrile, 4-methoxybenzaldehyde, and 2-hydroxybenzaldehyde, the LUMO of electrophilic centers (energies of − 4.436 eV, − 4.586 eV, and − 4.774 eV, respectively) reacted with HOMO (energy of − 7.631 eV) of the nucleophilic site of substrate **5** to form new substances **7–9** (cf. Fig. 2). Also, for reactions of thiosemicarbazide and hydrazine, HOMO of nucleophilic centers (energies of − 7.269 eV and − 11.069 eV, respectively) reacted with LUMO of the electrophilic site of substrate **5** (energy of − 3.813 eV) to form substances **11** and **12** (cf. Fig. 2).

### In vitro antiproliferative activity

The effects of substances **2–9** on cell viability were screened against colon cancer (HCT116) and breast cancer (MCF7) cell lines with different concentrations (from 1.56 to 100 µM) for each substance (cf. Supplementary Table 1 and Supplementary Figs. 2 and 3) using doxorubicin as a reference and MTT assay^45^. The findings displayed that the tested substances exhibited varying effects on the viability of cancer cell lines. The most potent substances against the two cell lines were pyrazolone **2** and cyanoethanohydrazone **5**.

The IC_50_ (half maximal inhibitory concentration) values were presented in Table 2. The results unveiled variable inhibitory activities by the examined compounds. A very strong effect was depicted by pyrazolone **2** against the two cell lines (IC_50_ = 7.39 ± 0.5 and 9.24 ± 0.7 µM, respectively). Also, a strong efficacy against the two cell lines was illustrated by substrate **5** (IC_50_ = 13.46 ± 1.1 and 16.43 ± 1.3 µM, respectively). A moderate effect was shown by compounds **3** and **7**. While, weak activity was disclosed by substances **4**, **8**, and **9**.

**Table 2:** IC_50_ values of the examined substances against HCT116 and MCF7 cell lines.

Compds	In vitro Cytotoxicity IC_50_ (µM)^a^	
	HCT116	MCF7
**Doxorubicin**	5.23 ± 0.3	4.17 ± 0.2
**2**	7.39 ± 0.5	9.24 ± 0.7
**3**	35.62 ± 2.2	28.86 ± 1.9
**4**	76.79 ± 3.9	64.16 ± 3.6
**5**	13.46 ± 1.1	16.43 ± 1.3
**7**	41.91 ± 2.4	37.43 ± 2.1
**8**	63.78 ± 3.7	58.06 ± 3.2
**9**	91.00 ± 4.8	69.99 ± 3.7

^a^IC_50_ (µM): 1–10 (very strong), 11–20 (strong), 21–50 (moderate), 51–100 (weak), and > 100 (non-cytotoxic). Data were displayed as mean ± SEM (*n* = 3, three independent repeats).

**Figure 3:**
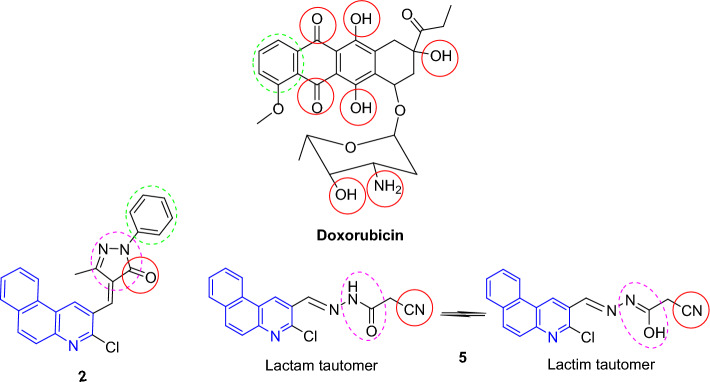
SAR of the potent compounds.

These experimental antitumor potentials may be ascribed to the reactive oxygen and nitrogen species represented in amide, lactam, and lactim tautomers^46^. Perhaps, the *N*-phenylpyrazolone scaffold (in compound **2**) might enhance the lipophilicity, potentially leading to improved binding affinity of the drug to hydrophobic regions of target proteins compared to other compounds^46,47^. Moreover, this was also in agreement with the theoretical data which showed the small energy gab (ΔE = E_LUMO_ − E_HOMO_) in pyrazolone **2** (cf. Table 1). That signifies it is more polarized (soft molecule) and reactive than hard ones because it easily offers electrons to an acceptor, so it was more bioactive substrate. The low energy gap values may be due to the groups entering into or extending the conjugation in these molecules in addition to tautomeric structures. The extended conjugation led to higher affinity to generate a face-to-edge aromatic interaction with receptor^5^. In turn, the inclusion of cyanoethanohydrazone scaffold (in compound **5**) might indorse favorable interactions, like H-bond formation through oxygen and nitrogen with specific receptors and target proteins enhancing the drug potency (cf. Fig. 3)^48,49^. Also, the electron-withdrawing group (like CN) was reported to exhibit a significant effect of improving the ability to inhibit cancer cell proliferation through binding interactions with receptor active sites via van-der Waals and H-bonding^50,51^.

**Figure 4:**
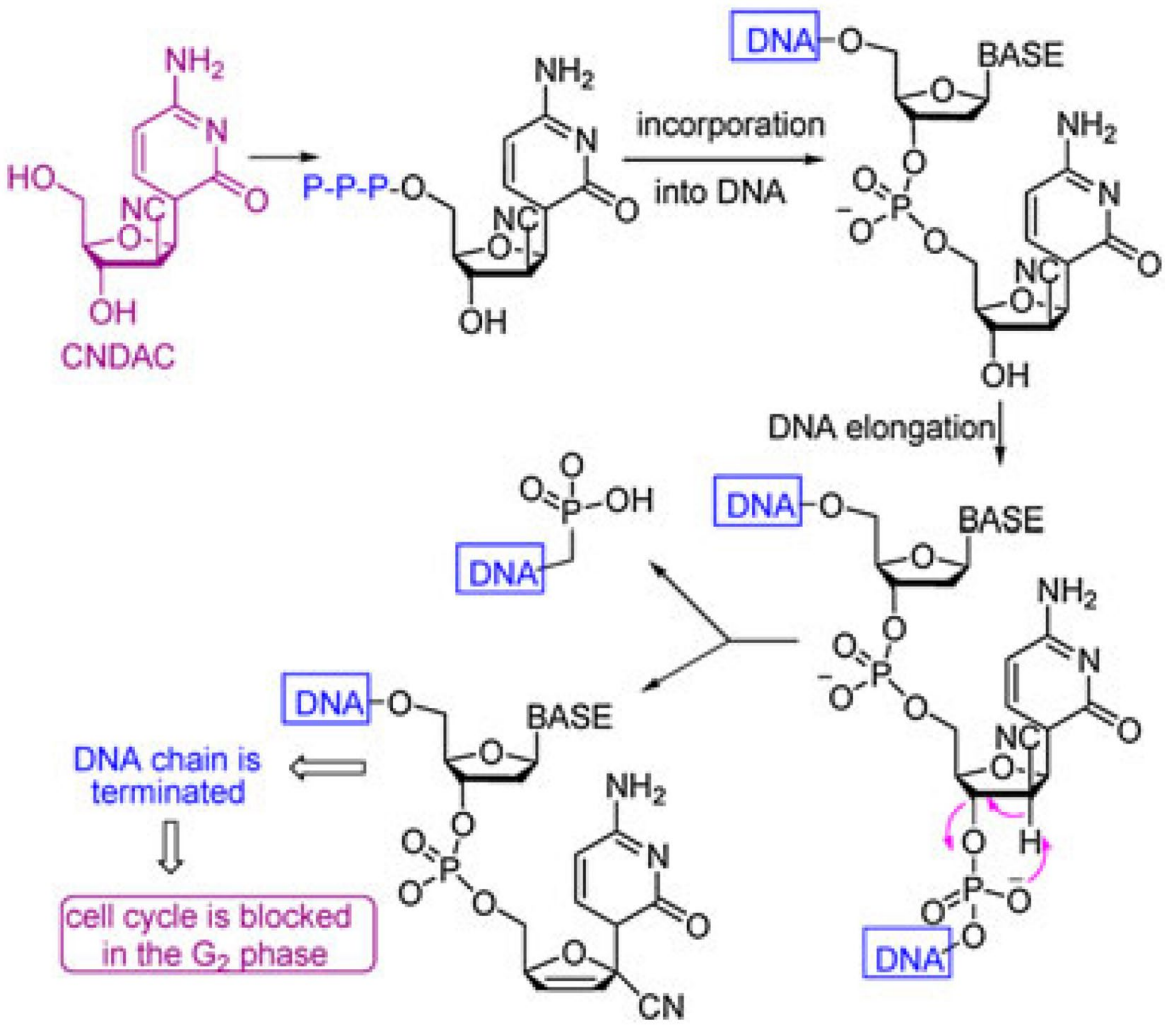
Pathway of the antitumor action of CNDAC.

**Figure 5:**
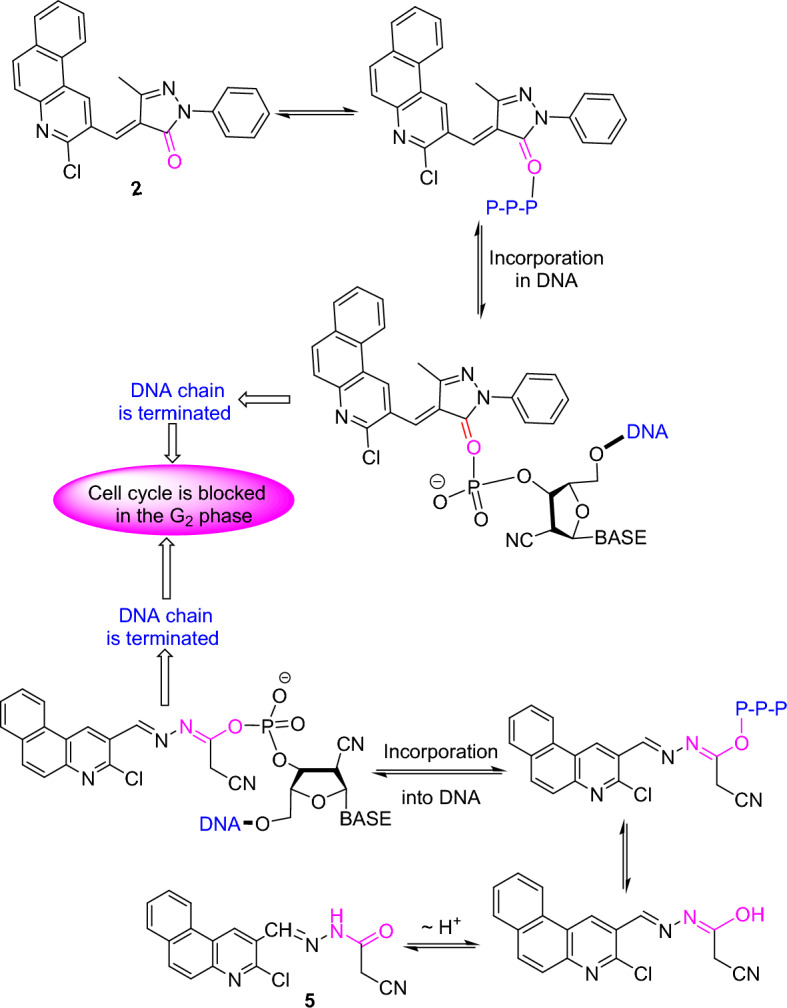
A proposed pathway for the antitumor action of compounds **2** and **5** compared to CNDAC.

Apparently, the antitumor action of the nucleoside analog, CNDAC (2′-C-cyano-2′-deoxy-1-*β*-d-arabinopentofuranosyl-cytosine) was assessed in clinical trials^52^. Incorporation of CNDAC triphosphate into DNA and extension during replication led to single-strand breakdowns causing cells arrest in G2 phase (Fig. 4). Accordingly, a proposed mechanism of DNA interaction with substances **2** and **5** as compared with CNDAC can be offered in Fig. 5. Also, tautomerism played a worthy role in anticancer agents by raising the functionality and flexibility^53^.

### Molecular docking

A molecular docking approach was performed with the molecular operating environment (MOE 2014.0901) to establish the binding energies of produced compounds toward cyclin-dependent kinase-5 protein (PDB ID: 3IG7) (http://www.rcsb.org/pdb) and govern the interactions between the formed ligands and receptors to compare the produced complexes affinities toward target binding sites of protein^54,55^. The binding affinity was measured by binding energies (*S*-score, kcal/mol) and H-bonding. The negative result of the docking score points to the stability of complex. All complexes acquired were docked in the same section of the binding site of the native co-crystallized ligand (EFP) (Tables 3 and 4). Table 3 also summarizes the specific amino-acids contributing to binding interaction between substances **2** and **5** with relevant target proteins. It imparts comprehensive information concerning the binding amino acids and the types of bonds established, like H-acceptor, pi-H, pi-cation, and others, for each substance in connection with the relevant protein target.

**Table 3:** Binding amino-acids in two compounds and a reference ligand to CDK-5 protein.

Compds	*S*-score (kcal/mol)	RMSD (Å)	Amino acids involved in bonding	
			H-bonding (bond length, Å)	Hydrophobic interaction (bond length, Å)
**2**	− 6.6320	0.9477	–	VAL 18 (4.07) VAL 18 (4.33) GLN 131 (4.82) ALA 144 (4.37)
**5**	− 6.5696	1.4889	HIS 84 (3.30) LYS 89 (3.54) LYS 89 (3.14) LYS 20 (3.72)	–
Co-crystallized ligand (EFP)	− 8.8422	1.9624	LEU 83 (2.97) GLU 81 (3.21) LEU 83 (3.05) LYS 33 (2.80)	ILE 10 (4.32)

**Table 4:** 2D and 3D interactions of compounds **2** and **5** with CDK-5 protein binding pockets.

Compds	2D	3D
2		
5		
Co-crystallized ligand (EFP)		

Among Table 3, the ligands binding energies were closer to that of co-crystallized ligand (EFP). Substance **2** exhibited a binding energy of − 6.6320 kcal/mol with the smallest RMSD of 0.9477 Å affirming to tightly binding to key nucleobases and amino-acids (through arene-H interactions with GLN 131, VAL 18, and ALA 144) of CDK-5 protein revealing its potential manipulation as DNA intercalator and CDK-5 inhibitor. Substance **5** pointed to a binding energy of − 6.5696 kcal/mol with RMSD 1.4889 Å, which revealed a remarkably strong binding affinity through H-bonding with LYS 20, HIS 84, and LYS 89 of the CDK-5 enzyme.

The docking analysis outcomes of compounds **2** and **5** with the CDK-5 protein can be portrayed in graphical 2D and 3D representations (cf. Table 4). 2D depiction illustrates detailed comprehensions of molecular interactions between prepared ligands and protein. Otherwise, 3D visualization platforms the binding interactions between these ligands and the CDK-5 protein, point up H-bonding interactions highlighted in red. Accordingly, binding affinities and docking interaction results provide an experience for the future development of CDK-5 enzyme inhibitors.

### Modeling pharmacokinetics studies

To direct the choice of selecting substrates from a vast collection of prepared substances in the initial stages of drug discovery, pharmacological activities, and advance for an effective drug, ADME profiles comprising physicochemical properties, lipophilicity, and drug-likeness of the produced substrate have been prophesied by SwissADME free web tool^56,57^. Substrates **1, 2,** and** 5** were found to follow Lipinski’s rule of five, with a total polar surface area (TPSA) of 29.96, 45.56, and 78.14 Å^2^, in addition to good lipophilicity, stated by consensus Log P_o/w_ which are in 3.36, 4.92, and 2.97, respectively. They revealed a high GI absorption and showed a good bioavailability score (0.55), as displayed in Supplementary Table 2.

Their skin permeation (Log K_P_) parameters were − 5.03, − 4.56, and − 5.76 cm/s, respectively, hence enhancing the accessibility of bioactive molecules through the skin. Their cytochrome P450 isoenzymes (CYP1A2/CYP2C19/CYP2C9/CYP2D6/CYP3A4), acting a main part in biotransformation of drugs through *O*-type oxidation reactions, have been likewise estimated (cf. Supplementary Table 2). The substrates’ bioavailability was also estimated according to their pink area on radar chart (cf. Supplementary Figs. 4–11). Regarding the absorption properties, they illustrated gastrointestinal tract (GIT) absorption upon their existence in the BOILED-EGG white area chart as presented in Supplementary Fig. 12. The substances were completely comprised in the pink area and justified their good predicted oral bioavailability specifically compounds **1, 2, 3, 4,** and **5**.

## Conclusion

Certain benzoquinoline-based heterocycles such as pyrazolone, imidazoline, pyridone, and chromene derivatives, were prepared by treating a 3-chlorobenzo[*f*]quinoline-2-carbaldehyde with *N*-phenylpyrazolone and various nitrogen nucleophiles. The behavior of the cyanoethanohydrazone obtained was examined towards certain carbon-centered electrophiles and nitrogen nucleophiles. Molecular docking, DFT, and modeling pharmacokinetics approaches were performed to support the findings. The pyrazolone **2** and cyanoethanohydrazone **5** exhibited the most potency, which was demonstrated by their molecular docking towards the CDK-5 enzyme. The binding energies of substances **2** and **5** were closer to that of co-crystallized ligand (EFP), which implies the notably powerful binding affinity towards CDK-5 enzyme. These substances were completely involved in the pink area and justified their good predicted oral bioavailability. Thus, pyrazolone **2** would be considered a promising candidate for further optimization to develop new chemotherapeutic agents.

## Materials and methods

### General

Melting points (uncorrected) were measured in open capillary tubes on a MEL-TEMP II electrothermal melting point apparatus. The elemental analyses were performed on a Perkin-Elmer 2400 CHN elemental analyzer (Perkin-Elmer, Waltham, MA) at the Faculty of Science, Ain Shams University. The infrared spectra (ν, cm^−1^) were recorded using the potassium bromide wafer technique on Fourier Transform Infrared Thermo Electron Nicolet iS10 Spectrometer (Thermo Fisher Scientific Inc. Waltham, MA) at Chemistry Department, Faculty of Science, and Faculty of Pharmacy, Ain Shams University. The ^1^H NMR spectra (δ, ppm) were measured on BRUKER 400 MHz Spectrometer at Faculty of Pharmacy, Cairo University, with tetramethyl silane (TMS) as an internal standard, using DMSO-*d*_6_ as a solvent. Mass spectra were carried out on direct probe controller inlet part to single quadrupole mass analyzer in (Thermo Scientific GCMS) MODEL (ISQ LT) using Thermo X-CALIBUR software at the regional center for mycology and biotechnology (RCMB), Al-Azhar University, Cairo, Egypt. Thin-layer chromatography (TLC) was run using TLC aluminum sheets silica gel F254 (Merck, Whitehouse Station, NJ).

### 4-((3-Chlorobenzo[f]quinolin-2-yl)methylene)-5-methyl-2-phenyl-2,4-dihydro-3H-pyrazol-3-one (2)

A solution of the aldehyde **1** (0.01 mol) and 5-methyl-2-phenyl-2,4-dihydro-3*H*-pyrazol-3-one (0.01 mol) in absolute ethanol (15 mL) involving piperidine (0.1 mL) was warmed for 10 min. then stirred at room temperature for 4 h. The solid obtained was collected and recrystallized from ethyl alcohol to give beige crystals, mp. 222–224 °C, yield 80%. IR (ν, cm^−1^): 1686 (C=O). ^1^H NMR (DMSO, δ, ppm): 2.32 (s, 3H, CH_3_), 7.16 (t, 1H, Ar–H, *J* = 7.2 *Hz*), 7.41 (t, 1H, Ar–H, *J* = 7.5 *Hz*), 7.73–8.12 (m, 9H, Ar–H), 8.69 (s, 1H, CH =), 8.97 (s, 1H, C4-H benzoquinoline). EIMS (*m/z*, %): 399.68 (M + 2, 7), 397.08 (M^+.^, 21), 371.75 (36), 362.94 (21), 343.57 (33), 342.75 (100), 331.98 (41), 312.47 (40), 286.53 (27), 237.69 (57), 230.90 (65), 214.45 (47), 172.73 (49), 159.79 (44), 128.80 (23), 125.91 (63), 111.36 (31), 93.56 (32), 79.24 (56), 67.75 (29). Anal. Calcd. for C_24_H_16_ClN_3_O (397.86): C, 72.45; H, 4.05; N, 10.56; Found: C, 72.32; H, 3.99; N, 10.59%.

### 1-(4-(((3-Chlorobenzo[f]quinolin-2-yl)methylene)amino)phenyl)ethan-1-one (3)

A solution of the aldehyde **1** (0.01 mol) and 4-acetylaniline (0.01 mol) in absolute ethanol (20 mL) including glacial acetic acid (0.2 mL) was heated under reflux for 4 h. The solid obtained was collected and recrystallized from ethyl alcohol to give pale-yellow crystals, mp. 200–202 °C, yield 83%. IR (ν, cm^−1^): 1685 (C=O). ^1^H NMR (DMSO, δ, ppm): 2.61 (s, 3H, CH_3_), 7.46 (d, 2H, Ar–H, *J* = 6.9 *Hz*), 7.79–7.89 (m, 3H, Ar–H), 8.06–8.11 (m, 3H, Ar–H), 8.93 (s, 1H, C4-H benzoquinoline), 9.01 (d, 2H, Ar–H, *J* = 7.3 *Hz*), 10.42 (s, 1H, CH = N). EIMS (*m/z*, %): 360.76 (M + 2, 22), 359.46 (49), 358.17 (M^+.^, 73), 332.70 (38), 329.28 (33), 310.34 (30), 308.46 (100), 290.79 (46), 278.16 (77), 252.14 (79), 232.64 (87), 221.11 (46), 185.77 (75), 177.89 (61), 157.50 (45), 156.74 (93), 133.14 (45), 122.60 (99), 110.71 (23), 88.87 (39), 81.99 (46). Anal. Calcd. for C_22_H_15_ClN_2_O (358.83): C, 73.64; H, 4.21; N, 7.81; Found: C, 73.53; H, 4.15; N, 7.79%.

### 3-Chloro-2-(4,5-dihydro-1H-imidazol-2-yl)benzo[f]quinoline (4)

A solution of the aldehyde **1** (0.01 mol) and 1,2-diaminoethane (0.01 mol) in absolute ethanol (20 mL) including glacial acetic acid (0.2 mL) was heated under reflux for 3 h. The solid obtained was collected and recrystallized from ethyl alcohol to offer white crystals, mp. 278–280 °C, yield 72%. IR (ν, cm^−1^): 3150 (NH), 1640 (C=N). ^1^H NMR (δ, ppm): 2.99 (t, 2H, NHCH_2_, *J* = 6.2 *Hz*), 3.85 (t, 2H, = NCH_2_, *J* = 6.2 *Hz*), 7.79 (br.s, 1H, NH, exchangeable), 7.81–8.07 (m, 5H, Ar–H), 8.83 (d, 1H, Ar–H, *J* = 7.6 *Hz*), 9.04 (s, 1H, C4-H benzoquinoline). EIMS (*m/z*, %): 283.17 (M + 2, 16), 281.56 (M^+.^, 45), 261.90 (40), 254.10 (58), 246.51 (100), 231.36 (23), 218.40 (28), 195.14 (29), 184.61 (40), 148.66 (60), 131.50 (75), 127.43 (73), 108.42 (40), 97.86 (34), 87.52 (51), 81.82 (58), 72.56 (39), 67.24 (41). Anal. Calcd. for C_16_H_12_ClN_3_ (281.74): C, 68.21; H, 4.29; N, 14.91; Found: C, 68.13; H, 4.24; N, 14.93%.

### E/Z-N′-((3-Chlorobenzo[f]quinolin-2-yl)methylene)-2-cyanoacetohydrazide (5)

A solution of aldehyde **1** (0.01 mol) and 2-cyanoethanohydrazide (0.01 mol) in absolute ethanol (20 mL) containing glacial acetic acid (0.2 mL) was refluxed for 3 h. The solid obtained while heating was collected and recrystallized from ethyl alcohol to give yellow crystals, mp. > 300 °C, yield 79%. IR (ν, cm^−1^): 3255 (NH), 2265 (C≡N), 1700 (C=O), 1602 (C=N). ^1^H NMR (δ, ppm): (*anti*- and *syn*-isomers, 80.3:19.7%); 7.77–8.08 (m, 6H, Ar-H), 12.15 (*br*.s, 1H, NH, exchangeable); for *anti*-isomer: 4.37 (s, 2H, CH_2_), 8.46 (s, 1H, CH=N), 8.99 (s, 1H, C4-H benzoquinoline); for *syn*-isomer: 3.89 (s, 2H, CH_2_), 8.64 (s, 1H, CH = N), 8.91 (s, 1H, C4-H benzoquinoline). EIMS (*m/z*, %): 324.50 (M + 2, 20), 322.35 (M^+.^, 52), 319.35 (64), 278.81 (62), 261.11 (42), 254.75 (100), 218.28 (17), 200.65 (35), 188.65 (38), 164.30 (45), 160.79 (41), 100.42 (25), 81.66 (73). Anal. Calcd. for C_17_H_11_ClN_4_O (322.75): C, 63.26; H, 3.44; N, 17.36; Found: C, 63.15; H, 3.39; N, 17.39%.

### E/Z-1-(((3-Chlorobenzo[f]quinolin-2-yl)methylene)amino)-4-(1,3-diphenyl-1H-pyrazol-4-yl)-6-imino-2-oxo-1,2,3,6-tetrahydropyridine-3,5-dicarbonitrile (7)

A mixture of **5** (1 mmol) and *2-((1,3-diphenyl-1H-pyrazol-4-yl)methylene)malononitrile* **(6)** (1 mmol) in 1,4-dioxane (15 mL) including triethylamine (0.1 mL) was refluxed for 5 h. The solid obtained upon cooling was collected, and recrystallized from ethyl alcohol to give brown crystals, mp. 290–292 °C, yield 68%. IR (ν, cm^−1^): 3146 (NH), 2205 (C≡N), 1667 (C=O). ^1^H NMR (δ, ppm): 3.40 (s, 1H, CH-CN), 7.49–8.23 (m, 16H, Ar–H), 8.60 (s, 1H, CH=N), 9.00 (s, 1H, C4-H benzoquinoline), 9.25 (s, 1H, C5-H pyrazole), 12.20 (*br*.s, 1H, NH, exchangeable). EIMS (*m/z*, %): 619.92 (M + 2, 9), 617.34 (M^+.^, 26), 582.75 (100), 548.69 (62), 547.13 (72), 467.48 (77), 418.68 (44), 396.73 (61), 366.35 (59), 347.56 (54), 313.27 (76), 280.73 (58), 215.82 (62), 186.81 (48), 176.12 (62), 167.28 (83), 113.84 (47), 101.23 (61), 92.29 (29), 65.25 (62). Anal. Calcd. for C_36_H_21_ClN_8_O (617.07): C, 70.07; H, 3.43; N, 18.16; Found: C, 70.00; H, 3.39; N, 18.19%.

### E/Z-N'-((3-Chlorobenzo[f]quinolin-2-yl)methylene)-2-cyano-3-(4-methoxyphenyl)acrylohydrazide (8)

A solution of **5** (1 mmol) and 4-methoxybenzaldehyde (1 mmol) in 1,4-dioxane (15 mL) containing piperidine (0.1 mL) was stirred at room temperature for 5 h. The deposited solid was collected and recrystallized from ethyl alcohol to give yellow crystals, mp. 286–288 °C, yield 81%. IR (ν, cm^−1^): 3275 (NH), 2211 (C≡N), 1697 (C=O). ^1^H NMR (δ, ppm): (*E*- and *Z*-isomers, 80:20%); 7.18 (d, 2H, Ar–H, *J* = 6.7 *Hz*), 7.79–8.09 (m, 9H, Ar–H + CH =); for *E*-isomer: 3.88 (s, 3H, OCH_3_), 8.29 (s, 1H, CH=N), 8.99 (s, 1H, C4-H benzoquinoline), 12.18 (*br*.s, 1H, NH, exchangeable); for *Z*-isomer: 4.37 (s, 3H, OCH_3_), 8.48 (s, 1H, CH=N), 9.02 (s, 1H, C4-H benzoquinoline), 12.31 (*br*.s, 1H, NH, exchangeable). EIMS (*m/z*, %): 442.07 (M + 2, 6), 440.23 (M^+.^, 20), 366.98 (91), 318.98 (59), 233.22 (17), 203.62 (100), 155.03 (47), 110.16 (28), 98.47 (59), 92.70 (81), 73.96 (52), 65.03 (37), 59.43 (38). Anal. Calcd. for C_25_H_17_ClN_4_O_2_ (440.89): C, 68.11; H, 3.89; N, 12.71; Found: C, 68.02; H, 3.82; N, 12.68%.

### E/Z-N'-((3-Chlorobenzo[f]quinolin-2-yl)methylene)-2-imino-2H-chromene-3-carbohydrazide (9)

A solution of cyanoacetamide derivative **5** (1 mmol) and 2-hydroxybenzaldehyde (1 mmol) in 1,4-dioxane (15 mL) containing piperidine (0.1 mL) was refluxed for 4 h. The deposited solid was collected and recrystallized from ethyl alcohol to produce white crystals, mp. 248–250 °C, yield 73%. IR (ν, cm^−1^): 3301 (NH), 1692 (C=O), 1636 (C=N). ^1^H NMR (δ, ppm): 7.05 (d, 1H, Ar–H, *J* = 6.5 *Hz*), 7.27 (t, 1H, Ar–H, *J* = 6.7 *Hz*), 7.37–8.09 (m, 8H, Ar–H), 8.48 (s, 1H, C4-H chromene), 8.73 (s, 1H, CH=N), 9.00 (s, 1H, C4-H benzoquinoline), 9.22 (*br*.s, 1H, = NH, exchangeable), 13.87 (*br*.s, 1H, NH, exchangeable). EIMS (*m/z*, %): 428.94 (M + 2, 35), 426.78 (M^+.^, 100), 401.14 (63), 389.20 (52), 388.56 (47), 358.57 (57), 309.58 (37), 277.86 (57), 256.84 (63), 222.27 (43), 187.22 (50), 166.10 (68), 143.64 (47), 124.20 (39), 66.17 (31). Anal. Calcd. for C_24_H_15_ClN_4_O_2_ (426.86): C, 67.53; H, 3.54; N, 13.13; Found: C, 67.41; H, 3.47; N, 13.16%.

### Reaction of cyanoacetohydrazone 5 with thiosemicarbazide

A solution of **5** (1 mmol) and thiosemicarbazide (1 mmol) in 1,4-dioxane (15 mL) was refluxed for 6 h. The solid obtained after cooling was collected and recrystallized from an ethyl alcohol/dioxane mixture (2:1) to produce thiosemicarbazone derivative **11** as white crystals, mp. 283–285 °C [Lit.^13^ 283–285 °C].

### Reaction of cyanoacetohydrazone 5 with hydrazine hydrate

A solution of **5** (1 mmol) and hydrazine hydrate (1 mmol, 80%) in ethyl alcohol (20 mL) was refluxed for 4 h. The solid obtained after cooling was collected and recrystallized from ethanol to produce *1,2-bis((3-chlorobenzo[f]quinolin-2-yl)methylene)hydrazine* (**12)** as yellow crystals, mp. > 300 °C [Lit.^13^ mp. > 300 °C].

### Cytotoxicity activity

#### Cell lines

Mammary gland breast cancer (MCF7) and colorectal carcinoma colon cancer (HCT116) cell lines were used for screening. The cell lines were obtained from ATCC via a holding company for biological products and vaccines (VACSERA). Doxorubicin was used as a reference anticancer drug for comparison.

#### Chemical reagents

RPMI-1640 medium, MTT, and DMSO were provided by Sigma Co. (USA), and Fetal Bovine serum was obtained from GIBCO (UK).

#### MTT assay

The mentioned cell lines were used to explore the inhibitory effects of substances on cell growth using the MTT assay^45^. This colorimetric assay was based on the conversion of the yellow tetrazolium bromide (MTT) to a purple formazan derivative by mitochondrial succinate dehydrogenase in viable cells. The stock samples of the substances were diluted with RPMI-1640 medium to desired concentrations ranging from 1.56 to 100 μM. The final concentration of DMSO in each sample did not exceed 1% (v/v). Doxorubicin was used as a positive control and DMSO was employed as a negative control. Cell lines were cultured in RPMI-1640 medium with 10% fetal bovine serum. Antibiotics added were 100 units/mL penicillin and 100 µM streptomycin at 37 °C in a 5% CO_2_ incubator. The cell lines were seeded in a 96-well plastic plate at a density of 1.0 × 10^4^ cells/well at 37 °C for 48 h under 5% CO_2_. After incubation, the cells were treated with different concentrations of substances and incubated for 48 h. After that, 20 µL of MTT solution at 5 mg/mL was added and incubated for 4 h. DMSO in volume of 100 µL was added into each well to dissolve the purple formazan formed. The colorimetric assay was measured and recorded at absorbance of 570 nm using a plate reader (EXL 800, USA). The relative cell viability in percentage was calculated as (A_570_ of treated samples/A_570_ of untreated sample) × 100. The IC_50_ values were determined according to the equation of Boltzmann sigmoidal concentration–response curves utilizing the non-linear regression fitting model^58^.

### Statistical analysis

The bioassay was repeated in triplicate. The data obtained were presented as means ± standard error of the means (SEM) (*n* = 3) using SPSS 13.0 program (SPSS Inc., Chicago, IL). Differences between groups were considered statistically significant at *p* values < 0.05.

### Supplementary Information

Supplementary Information 1.

Supplementary Information 2.

## Data Availability

All data generated or analyzed during this study are included in this published article and its supplementary information files.
