# Supplementary material for: Synthesis and in silico studies of certain benzo[f]quinoline-based heterocycles as antitumor agents
Source: Sci Rep. 2024 Jul 5;14:15522. doi: 10.1038/s41598-024-64785-z (PMC11226639; doi:10.1038/s41598-024-64785-z)
Supplement: Supplementary file 1 — Supplementary Information 1. [file 41598_2024_64785_MOESM1_ESM.pdf]

Synthesis and *In Silico* Studies of Certain Benzo[*f*]quinoline-based Heterocycles as Antitumor Agents

Eman A. E. El-Helw [1], Mahmoud Asran [2], Mohammad E. Azab [1], Maher H. Helal [2], Abdullah Y. A. Alzahrani [3], Sayed K. Ramadan [1,*]

[1] Chemistry Department, Faculty of Science, Ain Shams University, Cairo 11566, Egypt

[2] Chemistry Department, Faculty of Science, Helwan University, Ain-Helwan, Cairo, Egypt

[3] Chemistry Department, Faculty of Science and Arts, King Khalid University, Mohail Assir, Abha, Saudi Arabia

**E*-mail: sayed.karam2008@sci.asu.edu.eg

Supporting information:

DFT simulation:

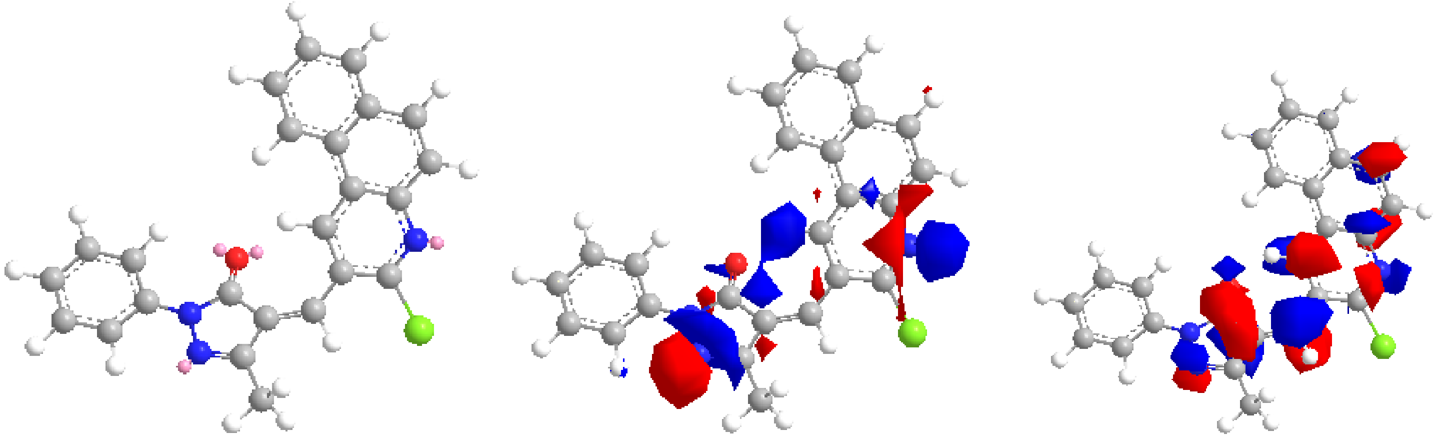

Compound 2

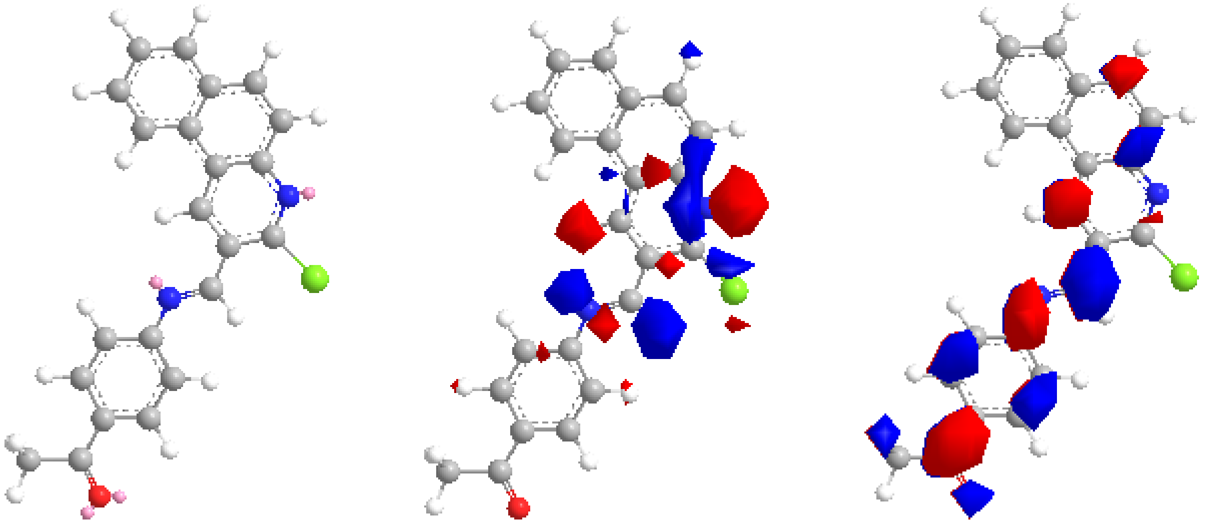

Compound 3

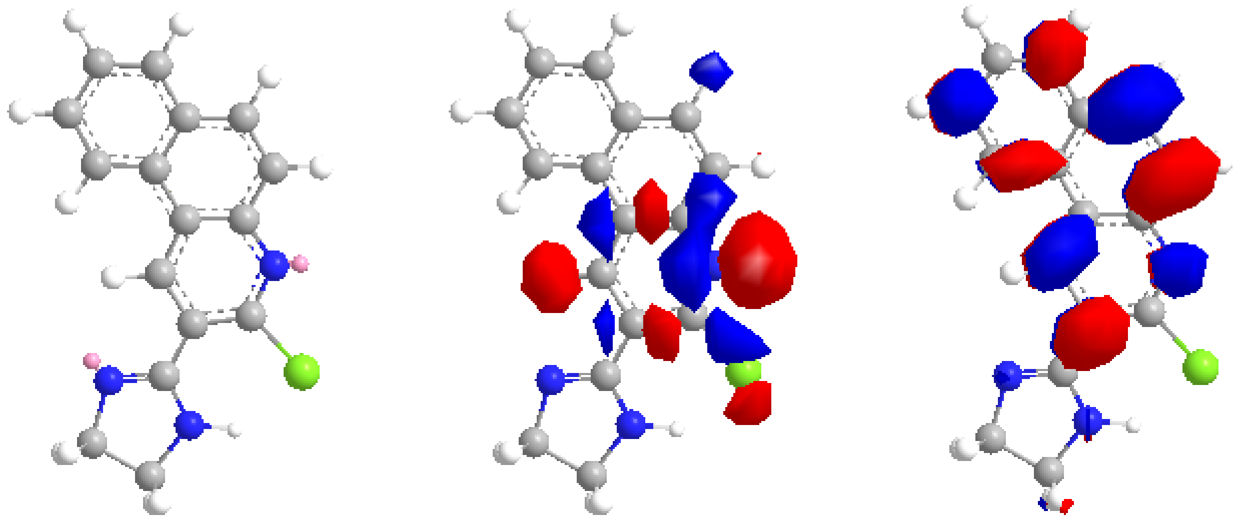

Compound 4

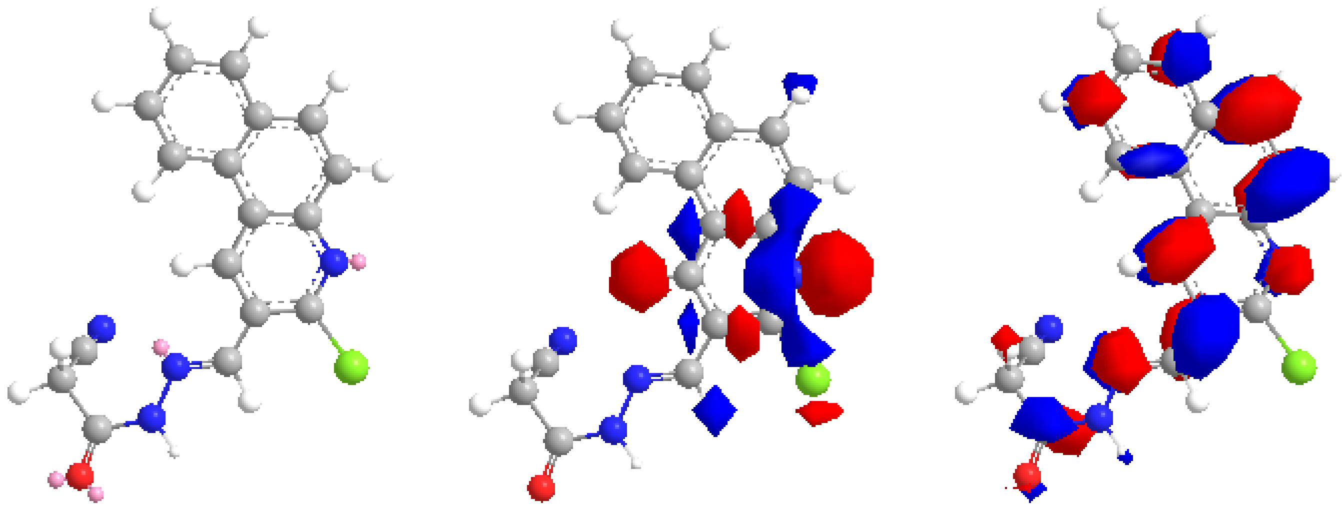

Compound 5

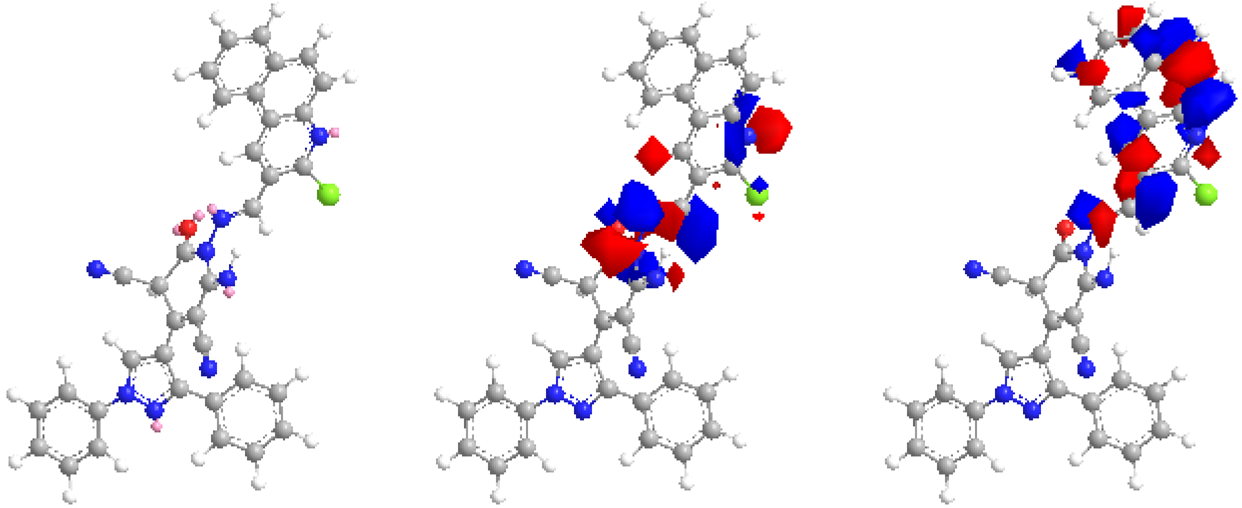

Compound 7

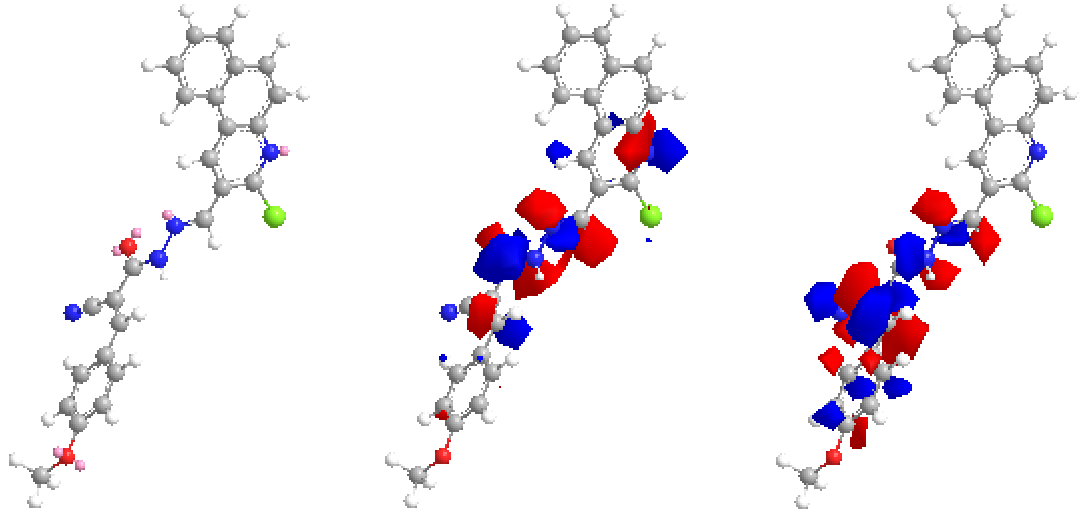

Compound 8

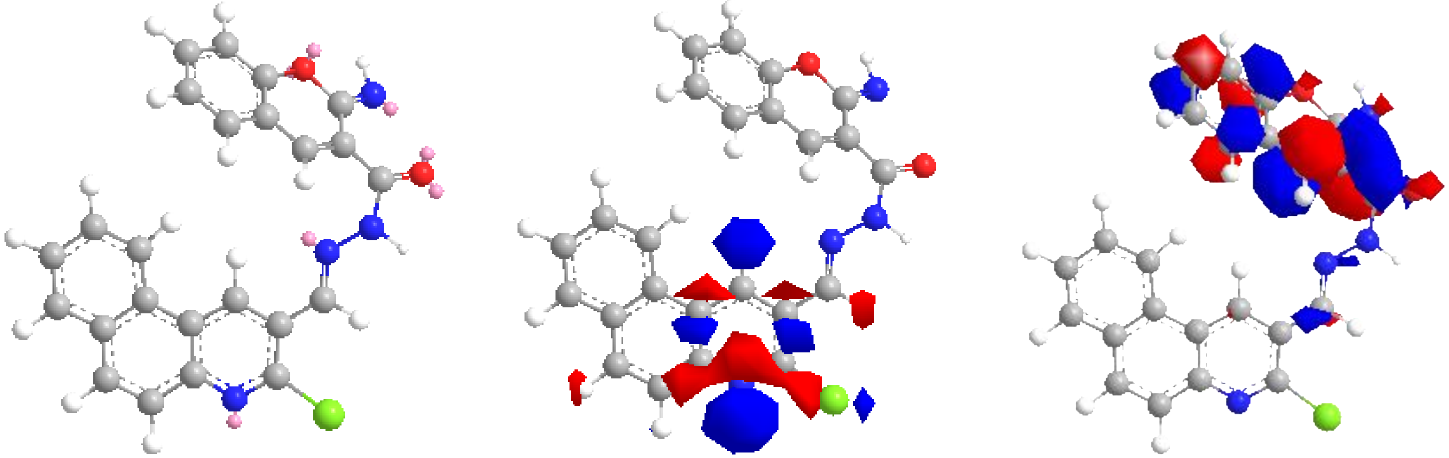

Compound 9

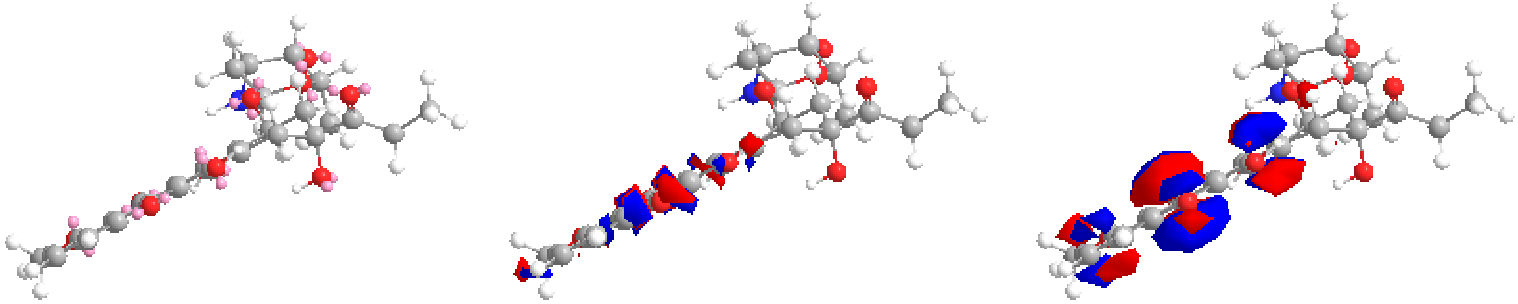

Doxorubicin

Supplementary Figure 1. Optimized structures (left), HOMO (middle), and LUMO (right) for compounds **2-13**.

Atom color index: Grey C, White H, Blue N, Red O, Yellow S, and Green Cl.

Supplementary Table 1. Average relative viability of cells (%).*

Conc. (μM)	HCT116	MCF7	Conc. (μM)	HCT116	MCF7
**Doxorubicin**			**Compound 7**		
**100**	7.1±1.1	6.2±1.2	**100**	34.5±0.2	28.2±1.4
**50**	13.9±1.4	10.9±1.4	**50**	44.9±1.1	43.5±0.9
**25**	18.7±1.3	14.3±1.5	**25**	59.2±0.8	60.2±1.1
**12.5**	31.4±1.5	26.9±1.1	**12.5**	70.3±0.5	65.3±0.7
**6.25**	47.9±1.7	41.5±1.3	**6.25**	91.4±0.4	95.5±0.3
**3.125**	60.5±1.9	58.4±1.1	**3.125**	100	100
**1.56**	73.8±1.6	69.1±1.9	**1.56**	100	100
**Compound 1**			**Compound 8**		
**100**	12.8±1.5	49.7±1.9	**100**	39.3±0.6	40.6±1.2
**50**	21.7±1.4	61.3±1.4	**50**	55.2±0.9	53.2±1.5
**25**	30.6±2.6	73.1±1.1	**25**	72.5±1.4	65.1±1.1
**12.5**	41.1±1.3	86.2±1.8	**12.5**	93.4±1.7	77.3±0.9
**6.25**	68.3±1.9	99.6±1.6	**6.25**	100	92.6±1.3
**3.125**	81.4±1.1	100	**3.125**	100	100
**1.56**	98.2±1.4	100	**1.56**	100	100
**Compound 2**			**Compound 9**		
**100**	8.3±0.7	7.8±0.9	**100**	49.1±1.3	44.8±1.2
**50**	17.2±1.3	19.4±1.1	**50**	65.8±1.4	56.2±0.8
**25**	24.1±1.6	22.5±0.8	**25**	82.9±0.9	70.2±1.4
**12.5**	32.4±1.2	34.2±1.2	**12.5**	97.3±0.6	81.4±0.5
**6.25**	55.7±1.1	58.1±1.8	**6.25**	100	96.6±1.1
**3.125**	66.2±1.9	85.3±1.4	**3.125**	100	100
**1.56**	88.1±1.3	97.6±0.7	**1.56**	100	100
**Compound 3**			**Compound 11**		
**100**	27.8±1.3	26.7±1.1	**100**	25.2±0.8	23.3±1.2
**50**	41.7±0.9	35.6±0.7	**50**	36.4±1.3	35.3±1.5
**25**	57.3±1.5	51.2±1.2	**25**	50.2±1.1	47.6±0.9

**12.5**	72.4±1.7	67.3±0.9	**12.5**	65.9±0.5	58.4±0.4
**6.25**	83.6±0.8	82.0±1.4	**6.25**	80.3±0.9	71.9±1.1
**3.125**	100	95.5±1.1	**3.125**	96.5±0.7	90.4±0.6
**1.56**	100	100	**1.56**	100	100
**Compound 4**			**Compound 12**		
**100**	47.2±1.2	43.8±1.3	**100**	54.4±0.7	53.3±0.3
**50**	56.3±1.5	55.3±1.4	**50**	71.9±0.4	72.9±0.4
**25**	76.4±0.8	62.7±1.1	**25**	84.6±0.6	84.6±0.7
**12.5**	88.9±1.2	85.2±1.5	**12.5**	99.7±0.3	98.8±0.2
**6.25**	100	96.3±1.3	**6.25**	100	100
**3.125**	100	100	**3.125**	100	100
**1.56**	100	100	**1.56**	100	100
**Compound 5**					
**100**	18.6±1.1	18.4±0.9			
**50**	25.2±0.9	27.1±0.6			
**25**	34.7±0.6	37.8±1.1			
**12.5**	40.5±0.5	52.4±1.3			
**6.25**	68.4±0.7	70.5±0.7			
**3.125**	85.3±0.3	86.2±0.8			
**1.56**	100	100			

* Data were displayed as mean ± SEM (n = 3) and $p < 0.05$.

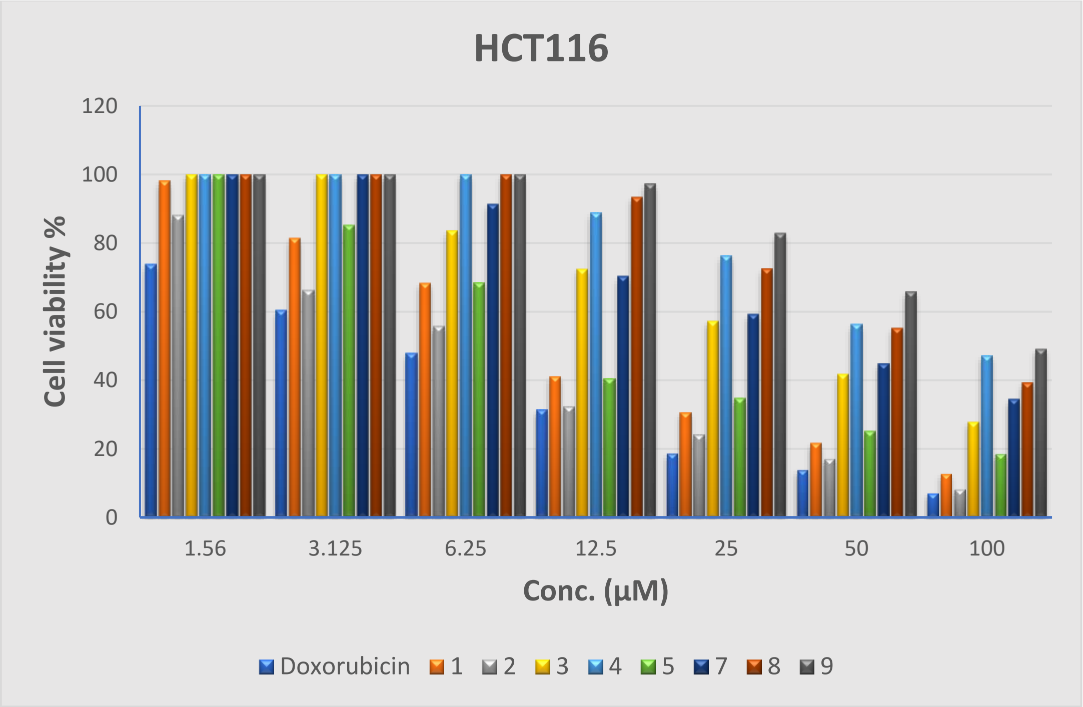

Supplementary Figure 2. Effect of the tested compounds on the cell viability of colon cancer cell line using different concentrations (1.56 to 100 µM) for each compound after treatment for 48 h compared to untreated cells.

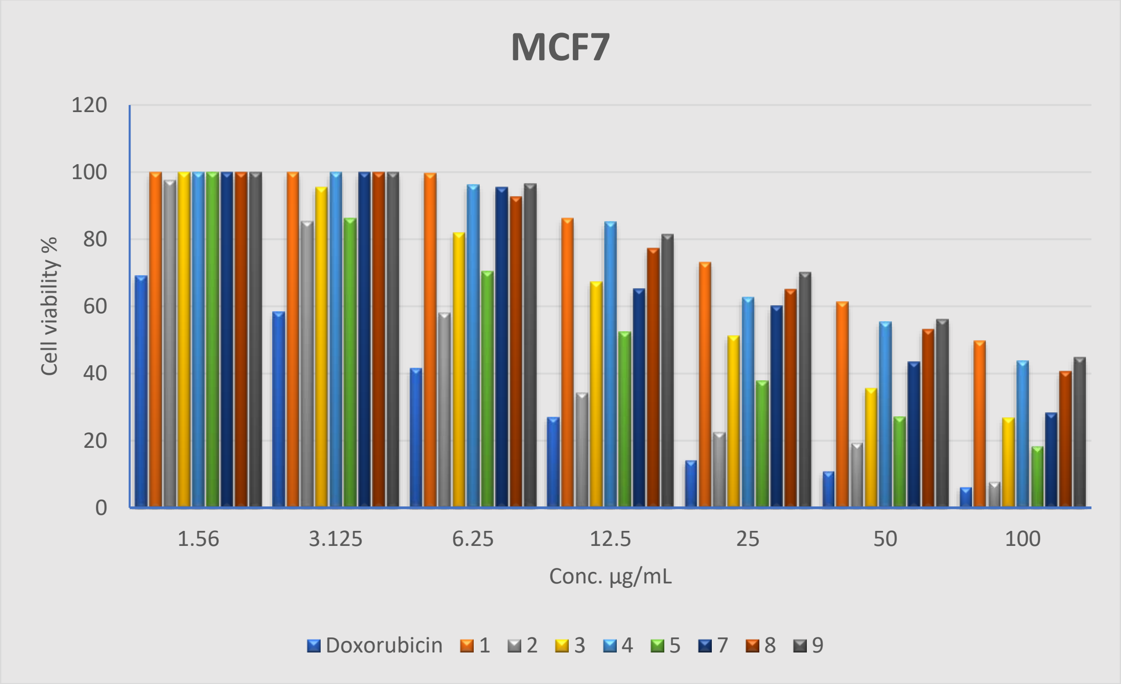

Supplementary Figure 3. Effect of the tested compounds on the cell viability of breast cancer cell line using different concentrations (1.56 to 100 µM) for each compound after treatment for 48 h compared to untreated cells.

Supplementary Table 2. ADME characteristics of compounds **1, 2,** and **5**.

**Entry**	**Compounds**		
	**1**	**2**	**5**
**Physicochemical Properties / Lipophilicity / Drug-likeness**			
Molecular weight (g/mol)	241.67	397.86	322.75
Num. heavy atoms	17	29	23
Num. arom. Heavy atoms	14	20	14
Fraction Csp3	0.00	0.04	0.06
Num. rotatable bonds	1	2	4
Num. H-bond acceptor	2	3	4
Num. H-bond donors	0	0	1
Molar Refractivity	69.65	125.72	90.08
TPSA ($Å^2$)	29.96	45.56	**78.14**
Consensus Log $P_{o/w}$	3.36	4.92	2.97
Lipinski's Rule	**Yes**	**Yes**	**Yes**
Bioavailability Score	**0.55**	**0.55**	**0.55**
**Pharmacokinetics**			
GI absorption	High	High	High
BBB permeant	Yes	Yes	Yes
P-gp substrate	No	No	No
CYP1A2 inhibitor	Yes	Yes	Yes
CYP2C19 inhibitor	Yes	Yes	Yes
CYP2C9 inhibitor	No	Yes	Yes
CYP2D6 inhibitor	No	No	No
CYP3A4 inhibitor	No	No	No
Log K_p (cm/s)	-5.03	-4.56	-5.76

(1)

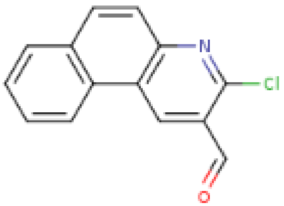

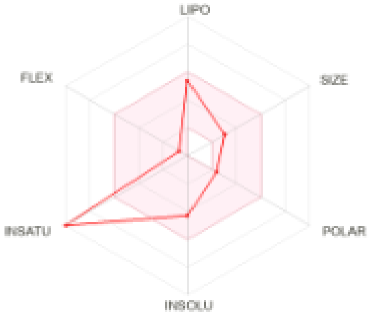

SMILES O=Cc1cc2c(nc1Cl)ccc1c2cccc1

Physicochemical Properties	
Formula	C14H8ClNO
Molecular weight	241.67 g/mol
Num. heavy atoms	17
Num. arom. heavy atoms	14
Fraction Csp3	0.00
Num. rotatable bonds	1
Num. H-bond acceptors	2
Num. H-bond donors	0
Molar Refractivity	69.65
TPSA	29.96 Å²

Lipophilicity	
Log $P_{o/w}$ (iLOGP)	2.25
Log $P_{o/w}$ (XLOGP3)	3.86
Log $P_{o/w}$ (WLOGP)	3.85
Log $P_{o/w}$ (MLOGP)	2.58
Log $P_{o/w}$ (SILICOS-IT)	4.26
Consensus Log $P_{o/w}$	3.36

Water Solubility	
Log *S* (ESOL)	-4.31
Solubility	1.17e-02 mg/ml ; 4.86e-05 mol/l
Class	Moderately soluble
Log *S* (Ali)	-4.19
Solubility	1.58e-02 mg/ml ; 6.52e-05 mol/l
Class	Moderately soluble
Log *S* (SILICOS-IT)	-5.95
Solubility	2.73e-04 mg/ml ; 1.13e-06 mol/l
Class	Moderately soluble

Pharmacokinetics	
GI absorption	High
BBB permeant	Yes
P-gp substrate	No
CYP1A2 inhibitor	Yes
CYP2C19 inhibitor	Yes
CYP2C9 inhibitor	No
CYP2D6 inhibitor	No
CYP3A4 inhibitor	No
Log K_p (skin permeation)	-5.03 cm/s

Druglikeness	
Lipinski	Yes; 0 violation
Ghose	Yes
Veber	Yes
Egan	Yes
Muegge	Yes
Bioavailability Score	0.55

Medicinal Chemistry	
PAINS	0 alert
Brenk	3 alerts: 2-halo_pyridine, aldehyde, polycyclic_aromatic_hydrocarbon_3
Leadlikeness	No; 2 violations: MW<250, XLOGP3>3.5
Synthetic accessibility	1.56

Supplementary Figure 4. ADME of compound **1**.

(2)

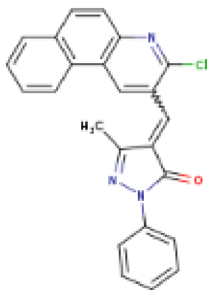

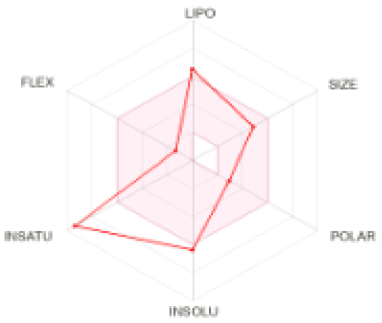

SMILES CC1=NN(C(=O)/C/1=C\c1cc2c(nc1Cl)ccc1c2cccc1)c1ccccc1

Physicochemical Properties	
Formula	C24H16ClN3O
Molecular weight	397.86 g/mol
Num. heavy atoms	29
Num. arom. heavy atoms	20
Fraction Csp3	0.04
Num. rotatable bonds	2
Num. H-bond acceptors	3
Num. H-bond donors	0
Molar Refractivity	125.72
TPSA	45.56 Å²

Lipophilicity	
Log $P_{o/w}$ (iLOGP)	3.64
Log $P_{o/w}$ (XLOGP3)	5.87
Log $P_{o/w}$ (WLOGP)	4.98
Log $P_{o/w}$ (MLOGP)	4.24
Log $P_{o/w}$ (SILICOS-IT)	5.88
Consensus Log $P_{o/w}$	4.92

Water Solubility	
Log *S* (ESOL)	-6.38
Solubility	1.65e-04 mg/ml ; 4.14e-07 mol/l
Class	Poorly soluble
Log *S* (Ali)	-6.60
Solubility	1.00e-04 mg/ml ; 2.52e-07 mol/l
Class	Poorly soluble
Log *S* (SILICOS-IT)	-8.94
Solubility	4.58e-07 mg/ml ; 1.15e-09 mol/l
Class	Poorly soluble

Pharmacokinetics	
GI absorption	High
BBB permeant	Yes
P-gp substrate	No
CYP1A2 inhibitor	Yes
CYP2C19 inhibitor	Yes
CYP2C9 inhibitor	Yes
CYP2D6 inhibitor	No
CYP3A4 inhibitor	No
Log K_p (skin permeation)	-4.56 cm/s

Druglikeness	
Lipinski	Yes; 1 violation: MLOGP>4.15
Ghose	Yes
Veber	Yes
Egan	Yes
Muegge	No; 1 violation: XLOGP3>5
Bioavailability Score	0.55

Medicinal Chemistry	
PAINS	2 alerts: ene_five_hetA1, ene_five_het_A
Brenk	3 alerts: 2-halo_pyridine, michael_acceptor_1, polycyclic_aromatic_hydrocarbon_3
Leadlikeness	No; 2 violations: MW>350, XLOGP3>3.5
Synthetic accessibility	3.49

Supplementary Figure 5. ADME of compound **2**.

(3)

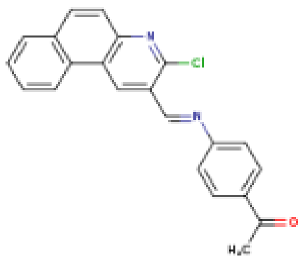

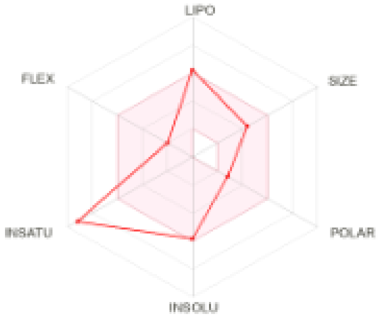

SMILES CC(=O)c1ccc(cc1)/N=C/c1cc2c(nc1Cl)ccc1c2cccc1

Physicochemical Properties	
Formula	C22H15ClN2O
Molecular weight	358.82 g/mol
Num. heavy atoms	26
Num. arom. heavy atoms	20
Fraction Csp3	0.05
Num. rotatable bonds	3
Num. H-bond acceptors	3
Num. H-bond donors	0
Molar Refractivity	108.15
TPSA	42.32 Å²

Lipophilicity	
Log $P_{o/w}$ (iLOGP)	3.40
Log $P_{o/w}$ (XLOGP3)	5.39
Log $P_{o/w}$ (WLOGP)	5.99
Log $P_{o/w}$ (MLOGP)	3.53
Log $P_{o/w}$ (SILICOS-IT)	6.43
Consensus Log $P_{o/w}$	4.95

Water Solubility	
Log *S* (ESOL)	-5.83
Solubility	5.29e-04 mg/ml ; 1.47e-06 mol/l
Class	Moderately soluble
Log *S* (Ali)	-6.03
Solubility	3.32e-04 mg/ml ; 9.27e-07 mol/l
Class	Poorly soluble
Log *S* (SILICOS-IT)	-8.89
Solubility	4.60e-07 mg/ml ; 1.28e-09 mol/l
Class	Poorly soluble

Pharmacokinetics	
GI absorption	High
BBB permeant	No
P-gp substrate	No
CYP1A2 inhibitor	Yes
CYP2C19 inhibitor	Yes
CYP2C9 inhibitor	Yes
CYP2D6 inhibitor	No
CYP3A4 inhibitor	Yes
Log K_p (skin permeation)	-4.66 cm/s

Druglikeness	
Lipinski	Yes; 0 violation
Ghose	No; 1 violation: WLOGP>5.6
Veber	Yes
Egan	No; 1 violation: WLOGP>5.88
Muegge	No; 1 violation: XLOGP3>5
Bioavailability Score	0.55

Medicinal Chemistry	
PAINS	0 alert
Brenk	3 alerts: 2-halo_pyridine, imine_1, polycyclic_aromatic_hydrocarbon_3
Leadlikeness	No; 2 violations: MW>350, XLOGP3>3.5
Synthetic accessibility	2.74

Supplementary Figure 6. ADME of compound **3**.

(4)

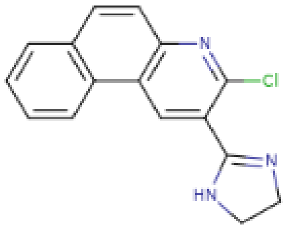

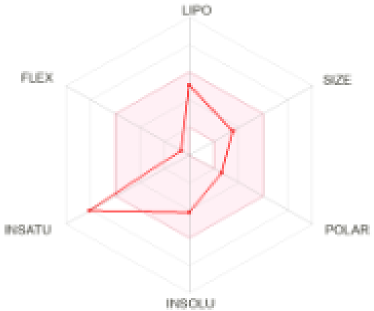

SMILES Clc1nc2ccc3c(c2cc1C1=NCCN1)cccc3

Physicochemical Properties	
Formula	C16H12ClN3
Molecular weight	281.74 g/mol
Num. heavy atoms	20
Num. arom. heavy atoms	14
Fraction Csp3	0.12
Num. rotatable bonds	1
Num. H-bond acceptors	2
Num. H-bond donors	1
Molar Refractivity	90.45
TPSA	37.28 Å²

Lipophilicity	
Log $P_{o/w}$ (iLOGP)	2.37
Log $P_{o/w}$ (XLOGP3)	3.36
Log $P_{o/w}$ (WLOGP)	2.63
Log $P_{o/w}$ (MLOGP)	3.27
Log $P_{o/w}$ (SILICOS-IT)	4.69
Consensus Log $P_{o/w}$	3.27

Water Solubility	
Log *S* (ESOL)	-4.16
Solubility	1.97e-02 mg/ml ; 6.99e-05 mol/l
Class	Moderately soluble
Log *S* (Ali)	-3.82
Solubility	4.26e-02 mg/ml ; 1.51e-04 mol/l
Class	Soluble
Log *S* (SILICOS-IT)	-6.90
Solubility	3.51e-05 mg/ml ; 1.25e-07 mol/l
Class	Poorly soluble

Pharmacokinetics	
GI absorption	High
BBB permeant	Yes
P-gp substrate	Yes
CYP1A2 inhibitor	Yes
CYP2C19 inhibitor	No
CYP2C9 inhibitor	No
CYP2D6 inhibitor	Yes
CYP3A4 inhibitor	Yes
Log K_p (skin permeation)	-5.63 cm/s

Druglikeness	
Lipinski	Yes; 0 violation
Ghose	Yes
Veber	Yes
Egan	Yes
Muegge	Yes
Bioavailability Score	0.55

Medicinal Chemistry	
PAINS	0 alert
Brenk	2 alerts: 2-halo_pyridine, polycyclic_aromatic_hydrocarbon_3
Leadlikeness	Yes
Synthetic accessibility	2.66

Supplementary Figure 7. ADME of compound **4**.

(5)

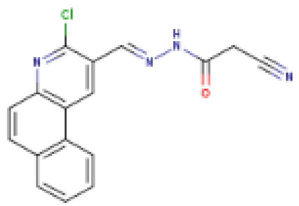

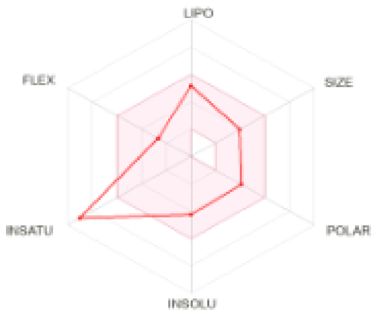

SMILES N#CCC(=O)N/N=C/c1cc2c(nc1Cl)ccc1c2cccc1

Physicochemical Properties	
Formula	C17H11ClN4O
Molecular weight	322.75 g/mol
Num. heavy atoms	23
Num. arom. heavy atoms	14
Fraction Csp3	0.06
Num. rotatable bonds	4
Num. H-bond acceptors	4
Num. H-bond donors	1
Molar Refractivity	90.08
TPSA	78.14 Å²

Lipophilicity	
Log $P_{o/w}$ (iLOGP)	2.16
Log $P_{o/w}$ (XLOGP3)	3.54
Log $P_{o/w}$ (WLOGP)	3.41
Log $P_{o/w}$ (MLOGP)	1.82
Log $P_{o/w}$ (SILICOS-IT)	3.94
Consensus Log $P_{o/w}$	2.97

Water Solubility	
Log *S* (ESOL)	-4.26
Solubility	1.78e-02 mg/ml ; 5.52e-05 mol/l
Class	Moderately soluble
Log *S* (Ali)	-4.87
Solubility	4.40e-03 mg/ml ; 1.36e-05 mol/l
Class	Moderately soluble
Log *S* (SILICOS-IT)	-6.55
Solubility	9.01e-05 mg/ml ; 2.79e-07 mol/l
Class	Poorly soluble

Pharmacokinetics	
GI absorption	High
BBB permeant	Yes
P-gp substrate	No
CYP1A2 inhibitor	Yes
CYP2C19 inhibitor	Yes
CYP2C9 inhibitor	Yes
CYP2D6 inhibitor	No
CYP3A4 inhibitor	No
Log K_p (skin permeation)	-5.76 cm/s

Druglikeness	
Lipinski	Yes; 0 violation
Ghose	Yes
Veber	Yes
Egan	Yes
Muegge	Yes
Bioavailability Score	0.55

Medicinal Chemistry	
PAINS	0 alert
Brenk	3 alerts: 2-halo_pyridine, imine_1, polycyclic_aromatic_hydrocarbon_3
Leadlikeness	No; 1 violation: XLOGP3>3.5
Synthetic accessibility	2.55

Supplementary Figure 8. ADME of compound **5**.

(7)

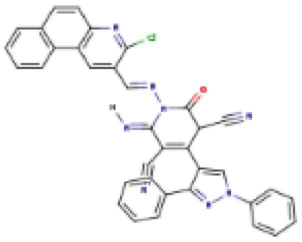

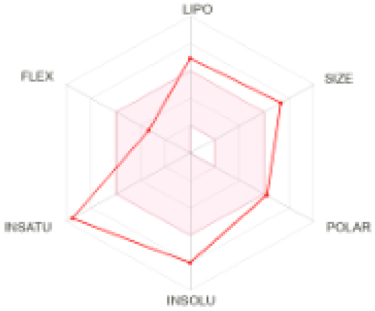

SMILES N#CC1C(=O)N(/N=C/c2cc3c(nc2Cl)ccc2c3cccc2)C(=N)C(=C1c1cn(nc1c1ccccc1)c1ccccc1)C#N

Physicochemical Properties	
Formula	C36H21ClN8O
Molecular weight	617.06 g/mol
Num. heavy atoms	46
Num. arom. heavy atoms	31
Fraction Csp3	0.03
Num. rotatable bonds	5
Num. H-bond acceptors	7
Num. H-bond donors	1
Molar Refractivity	181.91
TPSA	134.81 Å²

Lipophilicity	
Log $P_{o/w}$ (iLOGP)	3.74
Log $P_{o/w}$ (XLOGP3)	6.63
Log $P_{o/w}$ (WLOGP)	6.78
Log $P_{o/w}$ (MLOGP)	3.18
Log $P_{o/w}$ (SILICOS-IT)	6.23
Consensus Log $P_{o/w}$	5.31

Water Solubility	
Log *S* (ESOL)	-8.01
Solubility	6.01e-06 mg/ml ; 9.74e-09 mol/l
Class	Poorly soluble
Log *S* (Ali)	-9.26
Solubility	3.37e-07 mg/ml ; 5.47e-10 mol/l
Class	Poorly soluble
Log *S* (SILICOS-IT)	-11.88
Solubility	8.10e-10 mg/ml ; 1.31e-12 mol/l
Class	Insoluble

Pharmacokinetics	
GI absorption	Low
BBB permeant	No
P-gp substrate	No
CYP1A2 inhibitor	Yes
CYP2C19 inhibitor	No
CYP2C9 inhibitor	No
CYP2D6 inhibitor	No
CYP3A4 inhibitor	No
Log K_p (skin permeation)	-5.36 cm/s

Druglikeness	
Lipinski	Yes; 1 violation: MW>500
Ghose	No; 3 violations: MW>480, WLOGP>5.6, MR>130
Veber	Yes
Egan	No; 2 violations: WLOGP>5.88, TPSA>131.6
Muegge	No; 2 violations: MW>600, XLOGP3>5
Bioavailability Score	0.55

Medicinal Chemistry	
PAINS	1 alert: ene_cyano_C
Brenk	3 alerts: 2-halo_pyridine, imine_1, polycyclic_aromatic_hydrocarbon_3
Leadlikeness	No; 2 violations: MW>350, XLOGP3>3.5
Synthetic accessibility	4.93

Supplementary Figure 9. ADME of compound **7**.

(8)

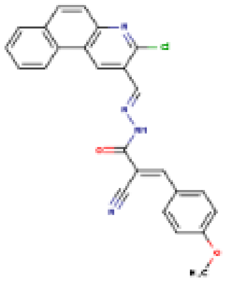

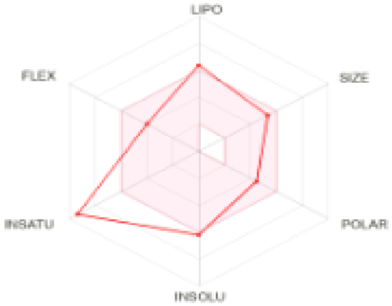

SMILES COc1ccc(cc1)/C=C(/C(=O)N/N=C/c1cc2c(nc1Cl)ccc1c2cccc1)\C#N

Physicochemical Properties	
Formula	C25H17ClN4O2
Molecular weight	440.88 g/mol
Num. heavy atoms	32
Num. arom. heavy atoms	20
Fraction Csp3	0.04
Num. rotatable bonds	6
Num. H-bond acceptors	5
Num. H-bond donors	1
Molar Refractivity	126.19
TPSA	87.37 Å²

Lipophilicity	
Log $P_{o/w}$ (iLOGP)	3.09
Log $P_{o/w}$ (XLOGP3)	5.64
Log $P_{o/w}$ (WLOGP)	5.00
Log $P_{o/w}$ (MLOGP)	2.74
Log $P_{o/w}$ (SILICOS-IT)	5.56
Consensus Log $P_{o/w}$	4.40

Water Solubility	
Log *S* (ESOL)	-6.19
Solubility	2.83e-04 mg/ml ; 6.41e-07 mol/l
Class	Poorly soluble
Log *S* (Ali)	-7.24
Solubility	2.55e-05 mg/ml ; 5.77e-08 mol/l
Class	Poorly soluble
Log *S* (SILICOS-IT)	-8.79
Solubility	7.13e-07 mg/ml ; 1.62e-09 mol/l
Class	Poorly soluble

Pharmacokinetics	
GI absorption	High
BBB permeant	No
P-gp substrate	No
CYP1A2 inhibitor	No
CYP2C19 inhibitor	Yes
CYP2C9 inhibitor	Yes
CYP2D6 inhibitor	No
CYP3A4 inhibitor	Yes
Log K_p (skin permeation)	-4.98 cm/s

Druglikeness	
Lipinski	Yes; 0 violation
Ghose	Yes
Veber	Yes
Egan	Yes
Muegge	No; 1 violation: XLOGP3>5
Bioavailability Score	0.55

Medicinal Chemistry	
PAINS	0 alert
Brenk	5 alerts: 2-halo_pyridine, conjugated_nitrile_group, imine_1, michael_acceptor_1, polycyclic_aromatic_hydrocarbon_3
Leadlikeness	No; 2 violations: MW>350, XLOGP3>3.5
Synthetic accessibility	3.32

Supplementary Figure 10. ADME of compound **8**.

(9)

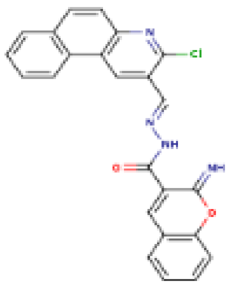

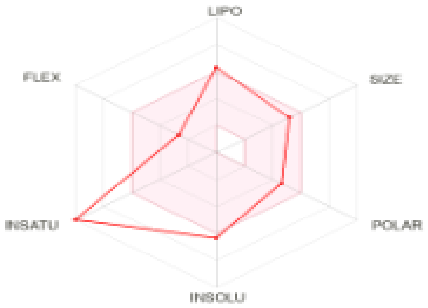

SMILES Clc1nc2ccc3c(c2cc1/C=N/NC(=O)c1cc2ccccc2oc1=N)cccc3

Physicochemical Properties	
Formula	C24H15ClN4O2
Molecular weight	426.85 g/mol
Num. heavy atoms	31
Num. arom. heavy atoms	24
Fraction Csp3	0.00
Num. rotatable bonds	4
Num. H-bond acceptors	5
Num. H-bond donors	2
Molar Refractivity	121.72
TPSA	91.34 Å²

Lipophilicity	
Log $P_{o/w}$ (iLOGP)	3.06
Log $P_{o/w}$ (XLOGP3)	5.60
Log $P_{o/w}$ (WLOGP)	5.03
Log $P_{o/w}$ (MLOGP)	3.51
Log $P_{o/w}$ (SILICOS-IT)	5.72
Consensus Log $P_{o/w}$	4.59

Water Solubility	
Log *S* (ESOL)	-6.32
Solubility	2.03e-04 mg/ml ; 4.75e-07 mol/l
Class	Poorly soluble
Log *S* (Ali)	-7.28
Solubility	2.24e-05 mg/ml ; 5.24e-08 mol/l
Class	Poorly soluble
Log *S* (SILICOS-IT)	-9.86
Solubility	5.91e-08 mg/ml ; 1.38e-10 mol/l
Class	Poorly soluble

Pharmacokinetics	
GI absorption	High
BBB permeant	No
P-gp substrate	No
CYP1A2 inhibitor	No
CYP2C19 inhibitor	Yes
CYP2C9 inhibitor	No
CYP2D6 inhibitor	No
CYP3A4 inhibitor	No
Log K_p (skin permeation)	-4.93 cm/s

Druglikeness	
Lipinski	Yes; 0 violation
Ghose	Yes
Veber	Yes
Egan	Yes
Muegge	No; 1 violation: XLOGP3>5
Bioavailability Score	0.55

Medicinal Chemistry	
PAINS	0 alert
Brenk	3 alerts: 2-halo_pyridine, imine_1, polycyclic_aromatic_hydrocarbon_3
Leadlikeness	No; 2 violations: MW>350, XLOGP3>3.5
Synthetic accessibility	3.57

Supplementary Figure 11. ADME of compound **9**.

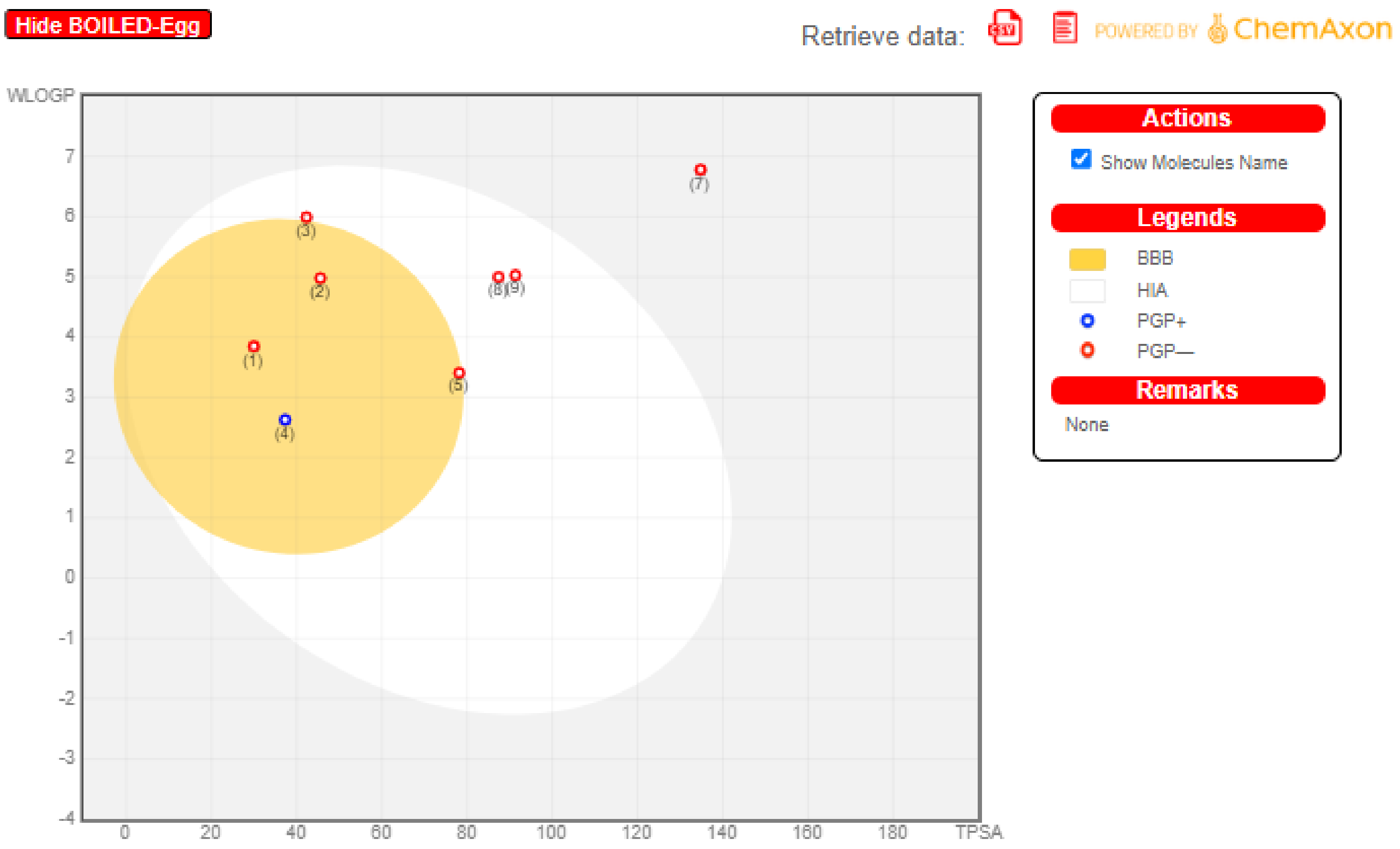

Supplementary Figure 12. BOILED-EGG chart of the synthesized substrates **1-9**.

Spectral data

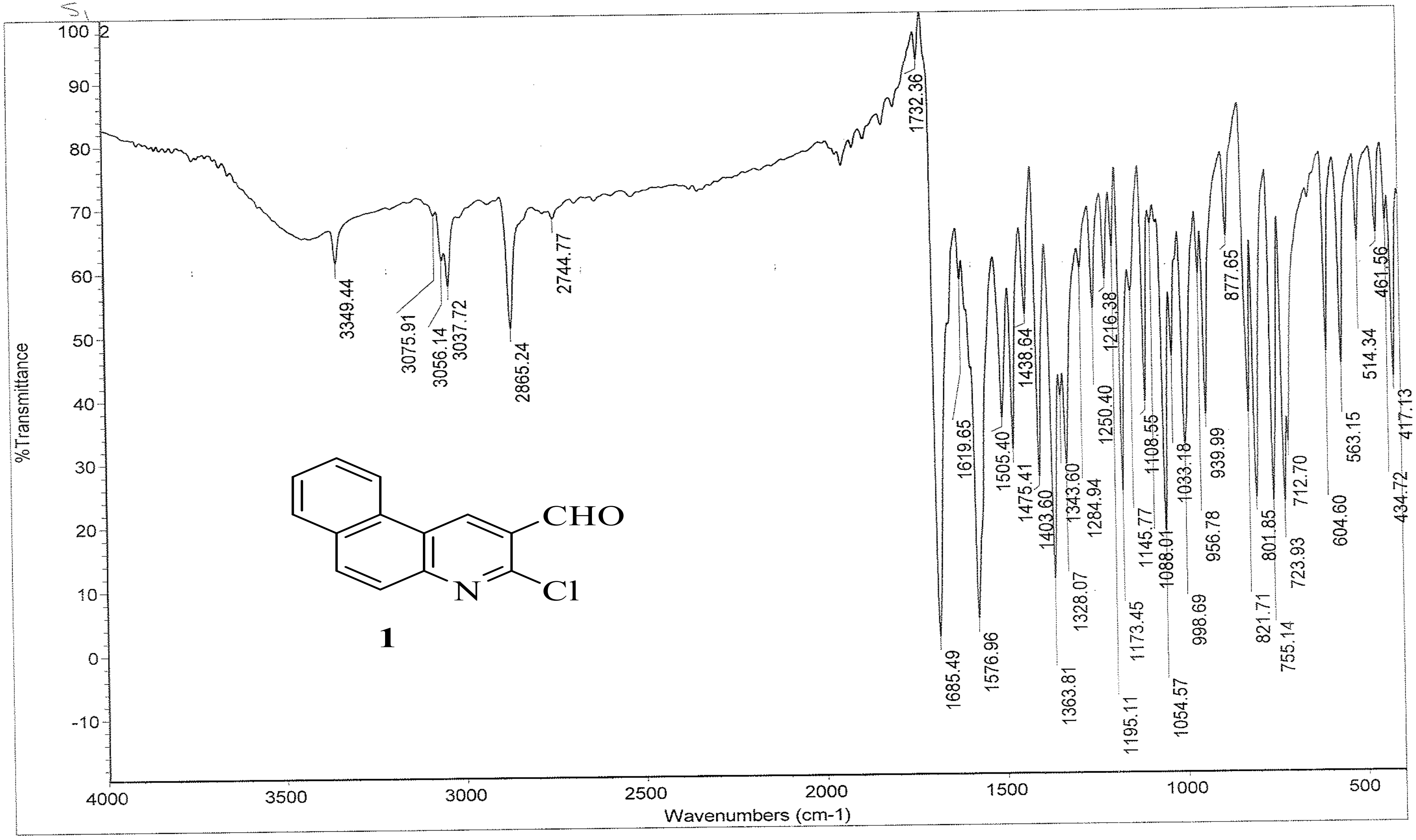

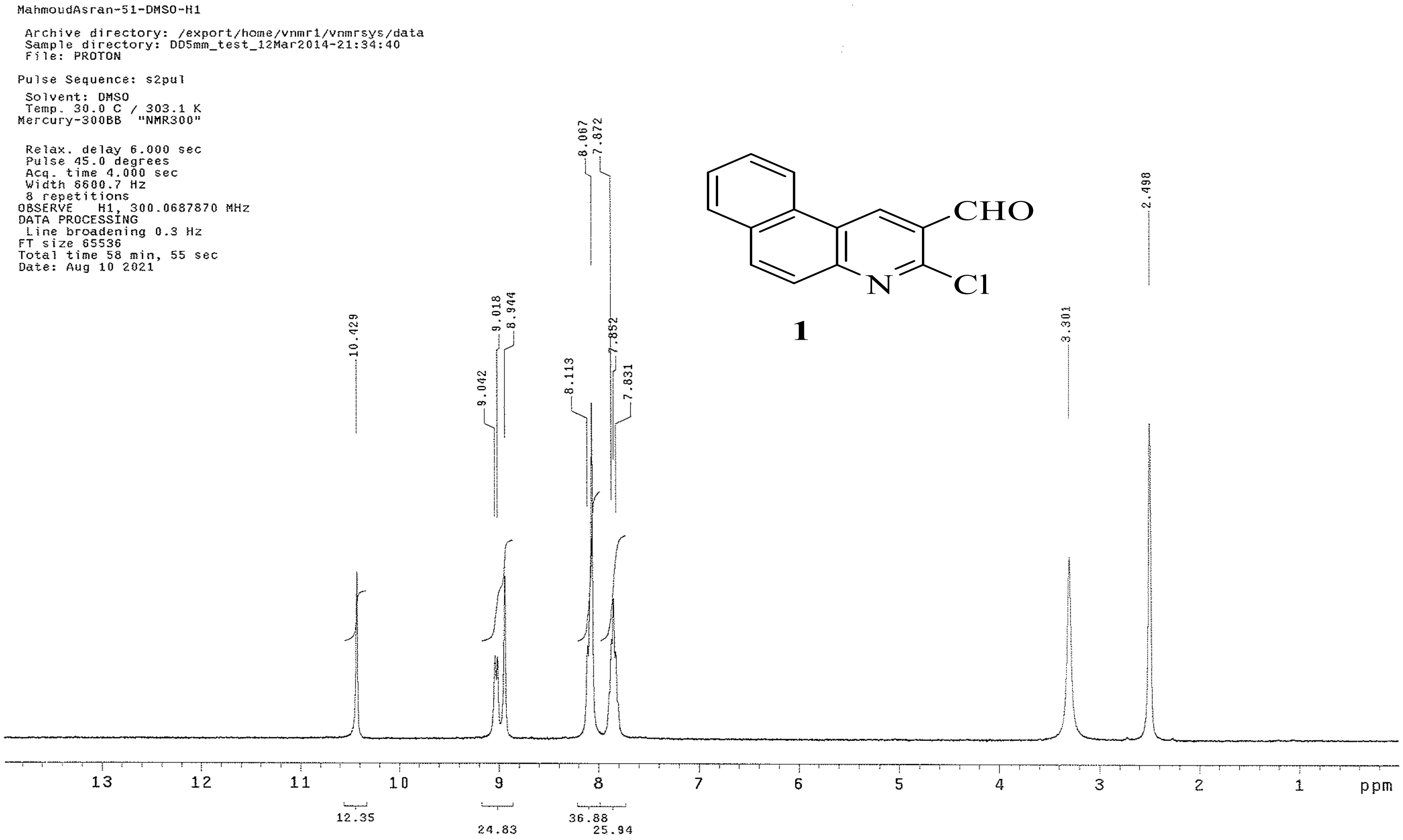
MahmoudAsran-51-DMSO-H1
Archive directory: /export/home/vnmr1/vnmrsys/data
Sample directory: DD5mm_test_12Mar2014-21:34:40
File: PROTON
Pulse Sequence: s2pul
Solvent: DMSO
Temp. 30.0 C / 303.1 K
Mercury-300BB "NMR300"
Relax. delay 6.000 sec
Pulse 45.0 degrees
Acq. time 4.000 sec
Width 6600.7 Hz
8 repetitions
OBSERVE H1, 300.0687870 MHz
DATA PROCESSING
Line broadening 0.3 Hz
FT size 65536
Total time 58 min, 55 sec
Date: Aug 10 2021
CHO
N
Cl
1
10.429
9.042
9.018
8.944
8.113
8.067
7.872
7.852
7.831
3.301
2.498
13
12
11
10
9
8
7
6
5
4
3
2
1
ppm
12.35
24.83
36.88
25.94

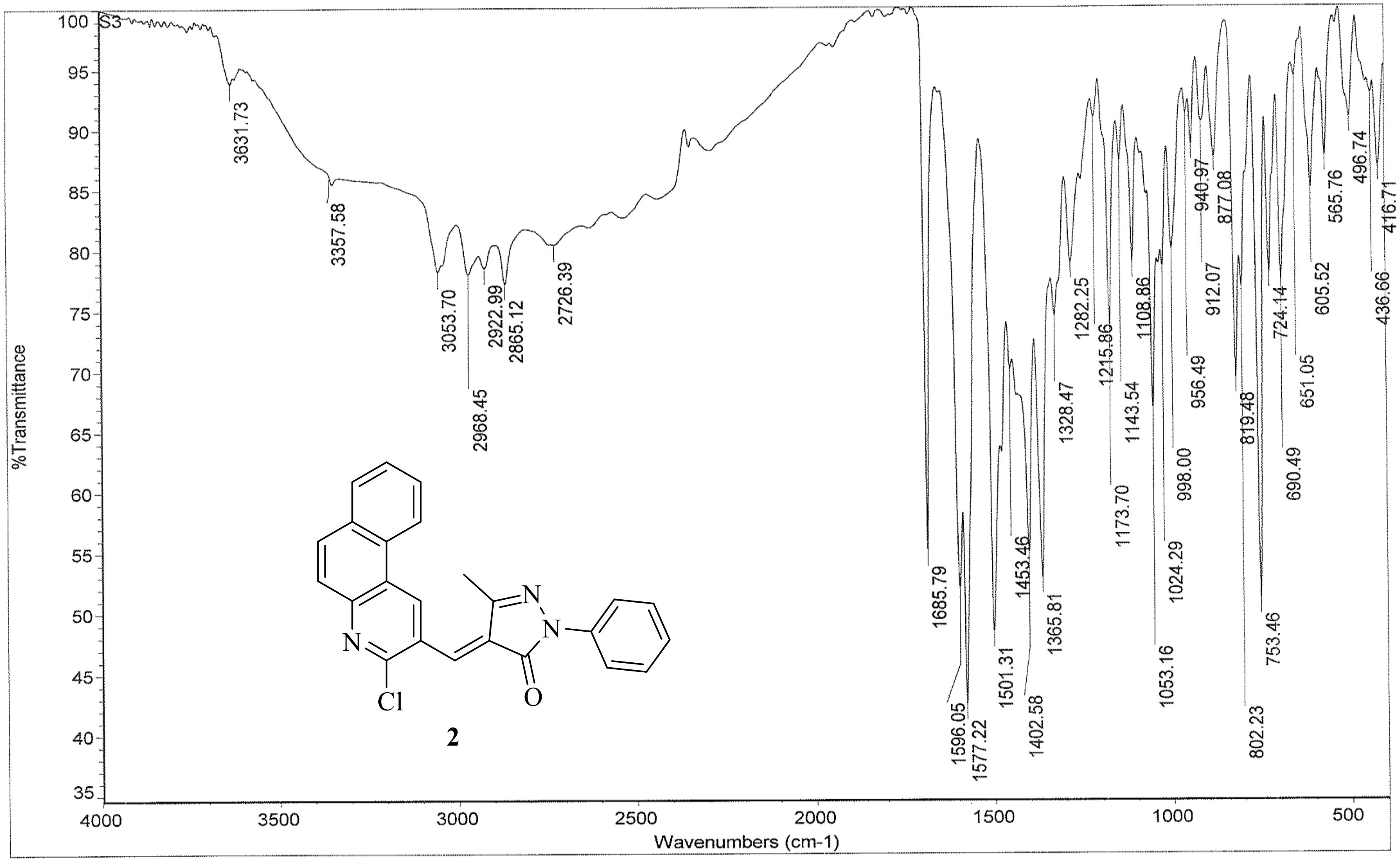
S3
%Transmittance
Wavenumbers (cm-1)
3631.73
3357.58
3053.70
2968.45
2922.99
2865.12
2726.39
1685.79
1596.05
1577.22
1501.31
1453.46
1402.58
1365.81
1328.47
1282.25
1215.86
1173.70
1143.54
1108.86
1053.16
1024.29
998.00
956.49
940.97
912.07
877.08
819.48
802.23
753.46
724.14
690.49
651.05
605.52
565.76
496.74
436.66
416.71
N
Cl
N
N
O
2

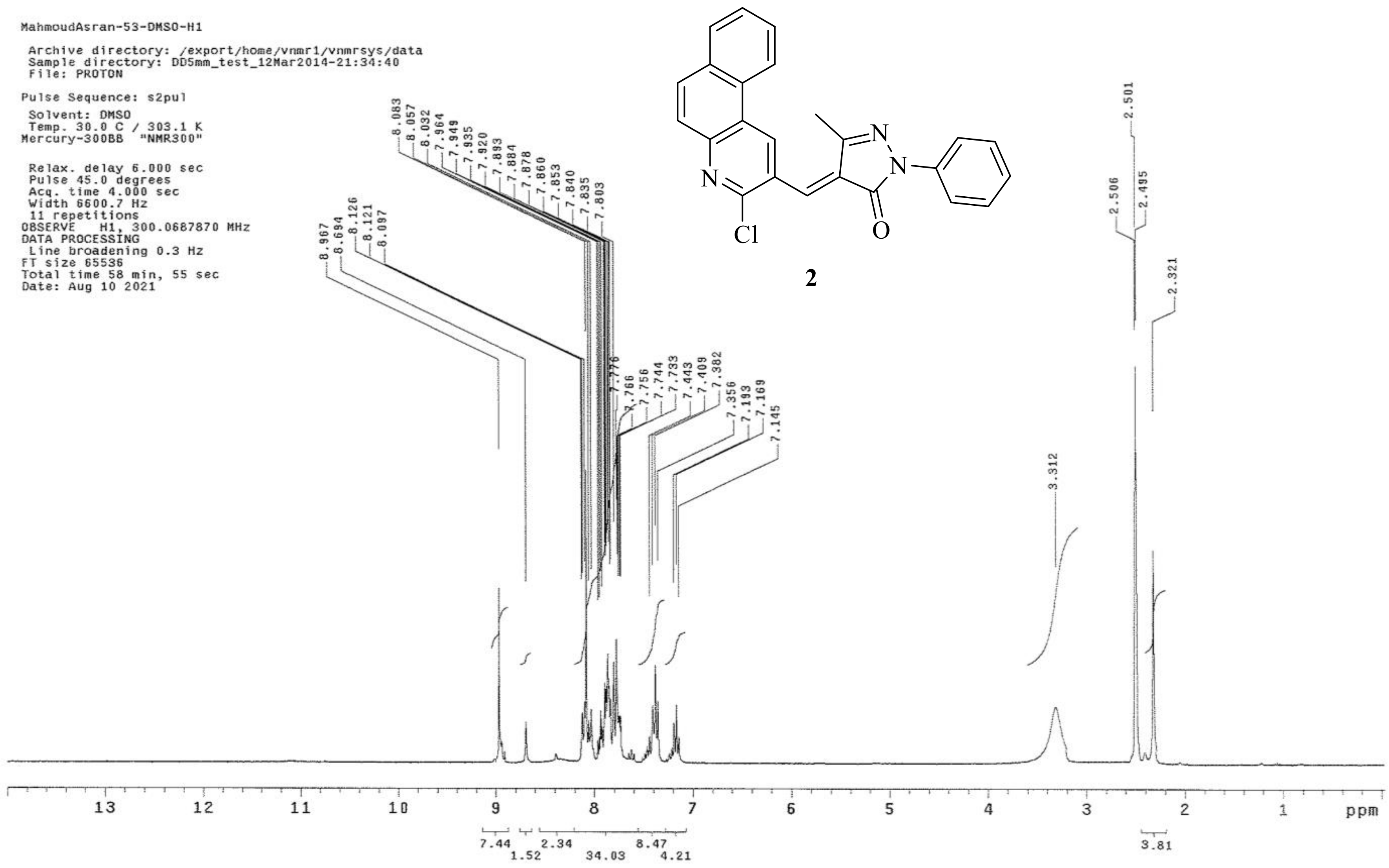
MahmoudAsran-53-DMSO-H1
Archive directory: /export/home/vnmr1/vnmrsys/data
Sample directory: DD5mm_test_12Mar2014-21:34:40
File: PROTON
Pulse Sequence: s2pul
Solvent: DMSO
Temp. 30.0 C / 303.1 K
Mercury-300BB "NMR300"
Relax. delay 6.000 sec
Pulse 45.0 degrees
Acq. time 4.000 sec
Width 6600.7 Hz
11 repetitions
OBSERVE H1, 300.0687870 MHz
DATA PROCESSING
Line broadening 0.3 Hz
FT size 65536
Total time 58 min, 55 sec
Date: Aug 10 2021
N
Cl
N
N
O
2
8.967 8.694 8.126 8.121 8.097
8.083 8.057 8.032 7.964 7.949 7.935 7.920 7.893 7.884 7.878 7.860 7.853 7.840 7.835 7.803
7.776 7.766 7.756 7.744 7.733 7.443 7.409 7.382 7.356 7.193 7.169 7.145
3.312
2.506 2.501 2.495 2.321
13 12 11 10 9 8 7 6 5 4 3 2 1 ppm
7.44 1.52 2.34 34.03 8.47 4.21 3.81

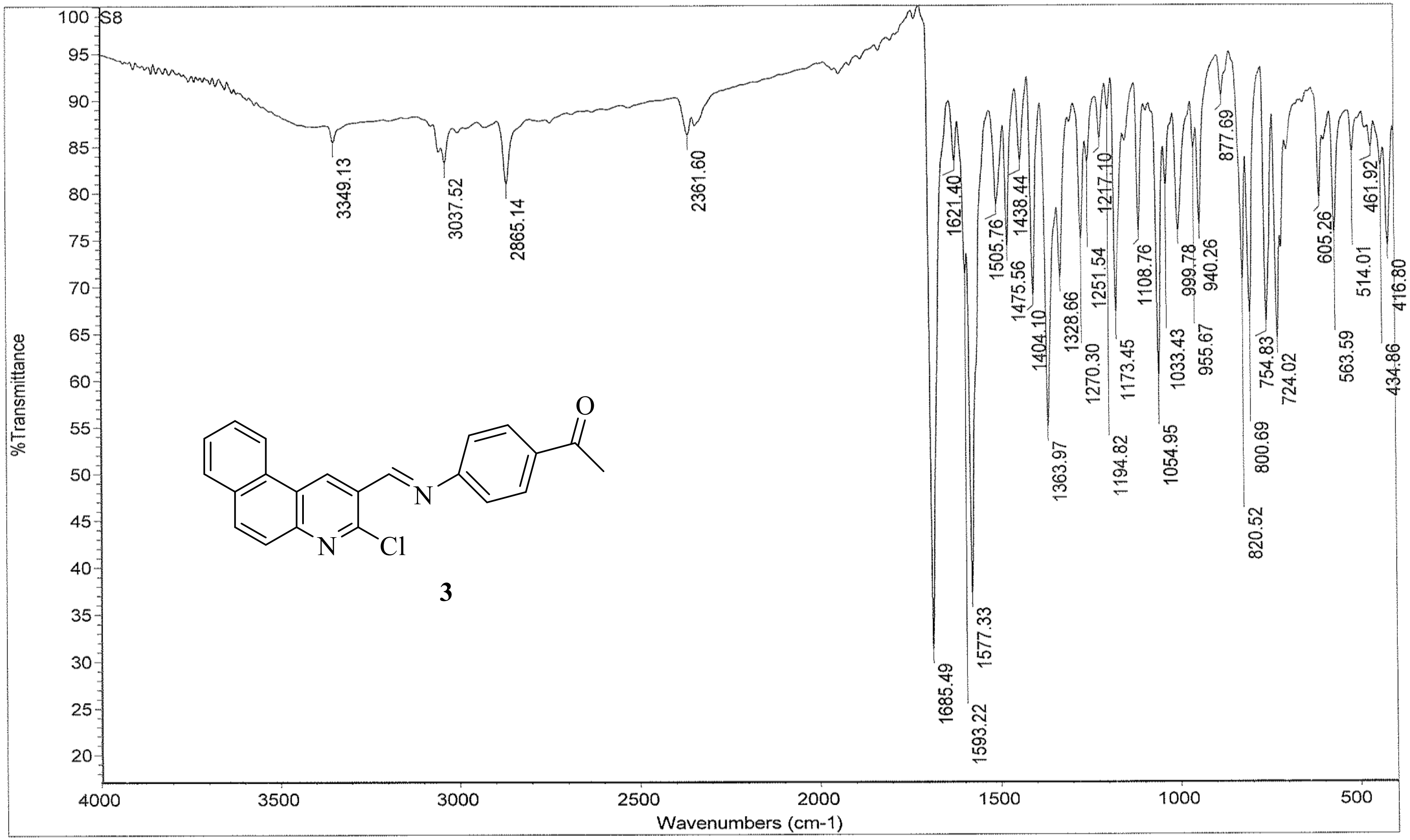
S8
%Transmittance
Wavenumbers (cm-1)
3349.13
3037.52
2865.14
2361.60
1685.49
1621.40
1593.22
1577.33
1505.76
1475.56
1438.44
1404.10
1363.97
1328.66
1270.30
1251.54
1217.10
1194.82
1173.45
1108.76
1054.95
1033.43
999.78
955.67
940.26
877.69
820.52
800.69
754.83
724.02
605.26
563.59
514.01
461.92
434.86
416.80
O
N
N
Cl
3

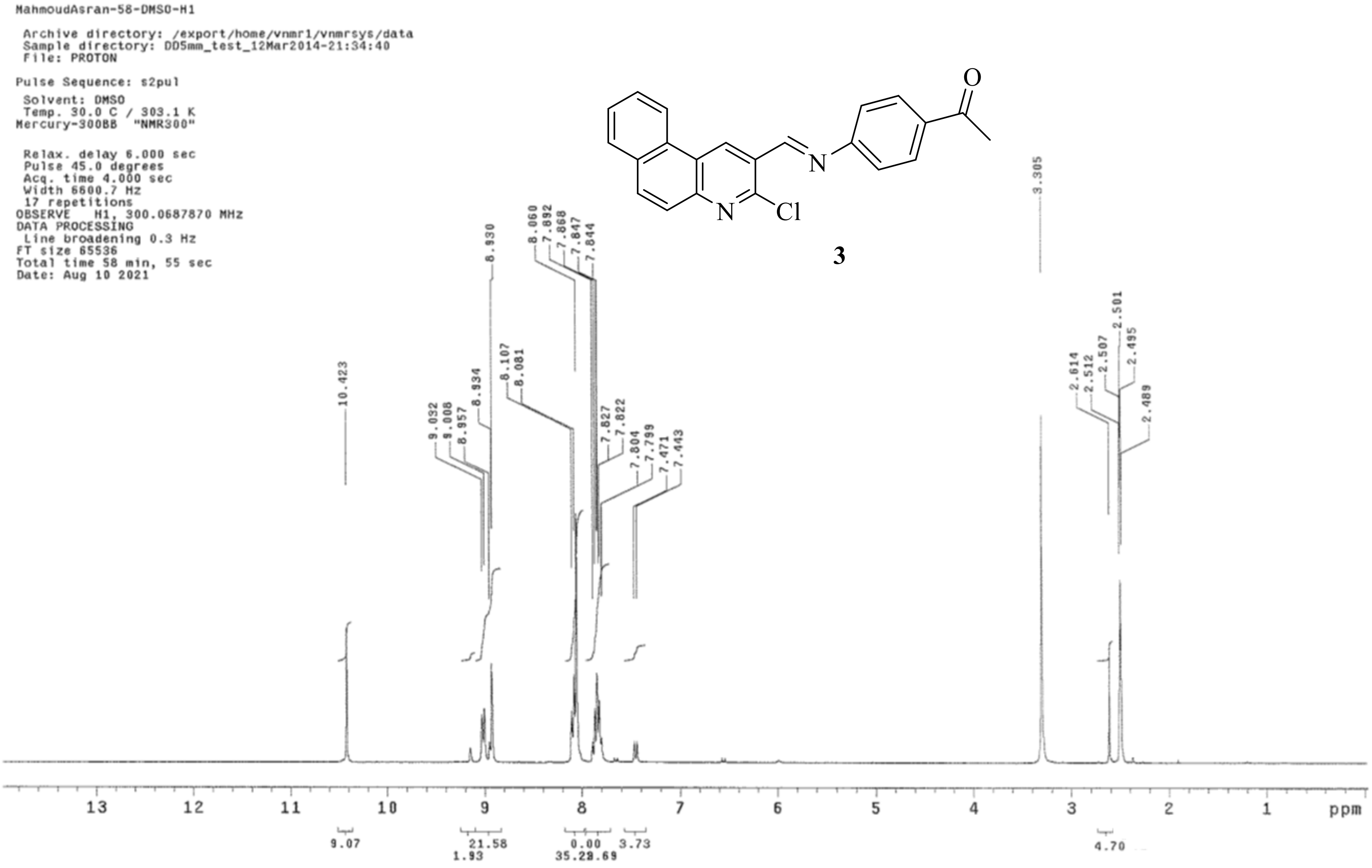
MahmoudAsran-58-DMSO-H1
Archive directory: /export/home/vnmr1/vnmrsys/data
Sample directory: DD5mm_test_12Mar2014-21:34:40
File: PROTON
Pulse Sequence: s2pul
Solvent: DMSO
Temp. 30.0 C / 303.1 K
Mercury-300BB "NMR300"
Relax. delay 6.000 sec
Pulse 45.0 degrees
Acq. time 4.000 sec
Width 6600.7 Hz
17 repetitions
OBSERVE H1, 300.0687870 MHz
DATA PROCESSING
Line broadening 0.3 Hz
FT size 65536
Total time 58 min, 55 sec
Date: Aug 10 2021
O
N
N
Cl
3
ppm

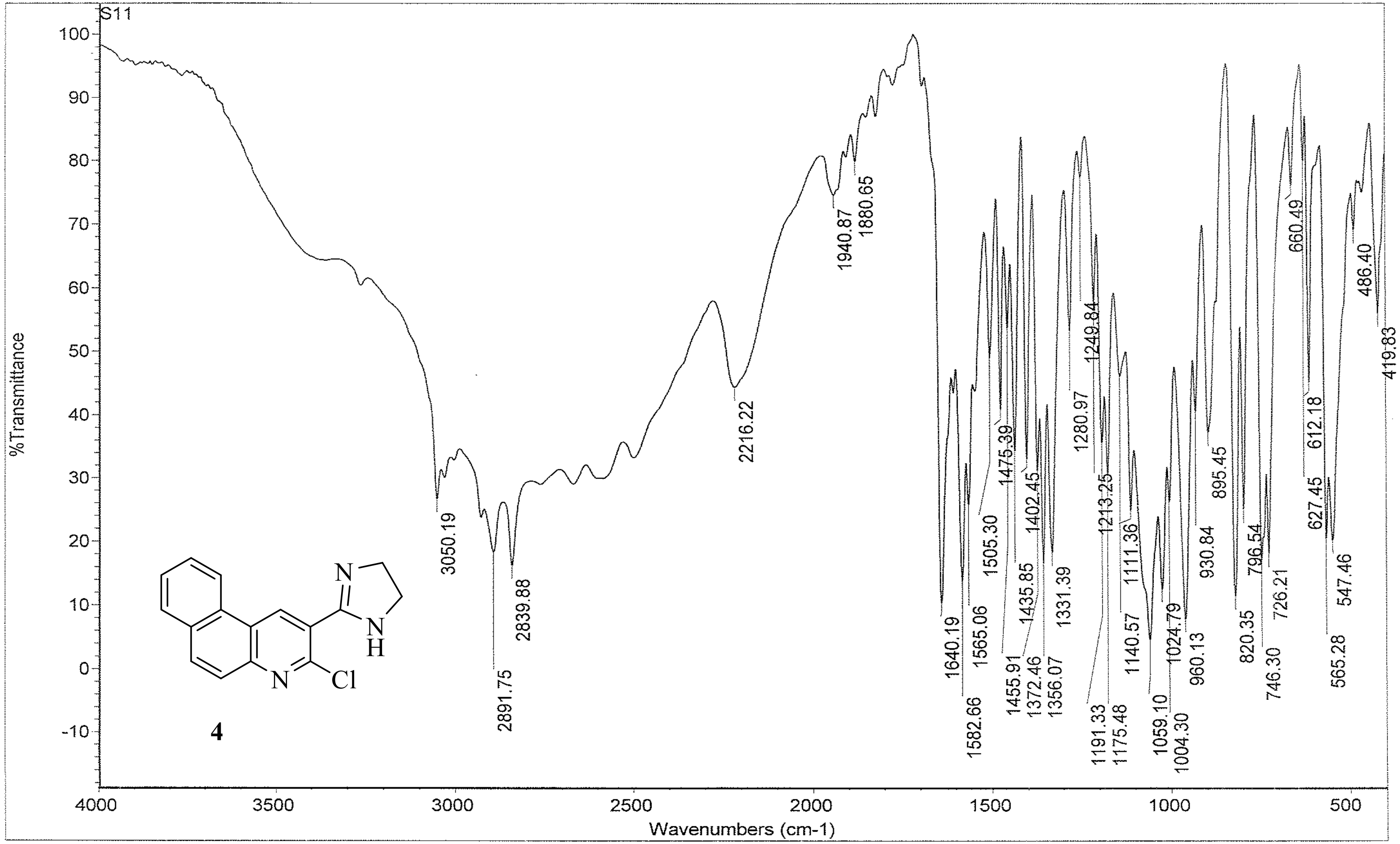
S11
%Transmittance
Wavenumbers (cm-1)
3050.19
2891.75
2839.88
2216.22
1940.87
1880.65
1640.19
1582.66
1565.06
1505.30
1475.39
1455.91
1435.85
1402.45
1372.46
1356.07
1331.39
1280.97
1249.84
1213.25
1191.33
1175.48
1140.57
1111.36
1059.10
1024.79
1004.30
960.13
930.84
895.45
820.35
796.54
746.30
726.21
660.49
627.45
612.18
565.28
547.46
486.40
419.83
N
N
H
N
Cl
4

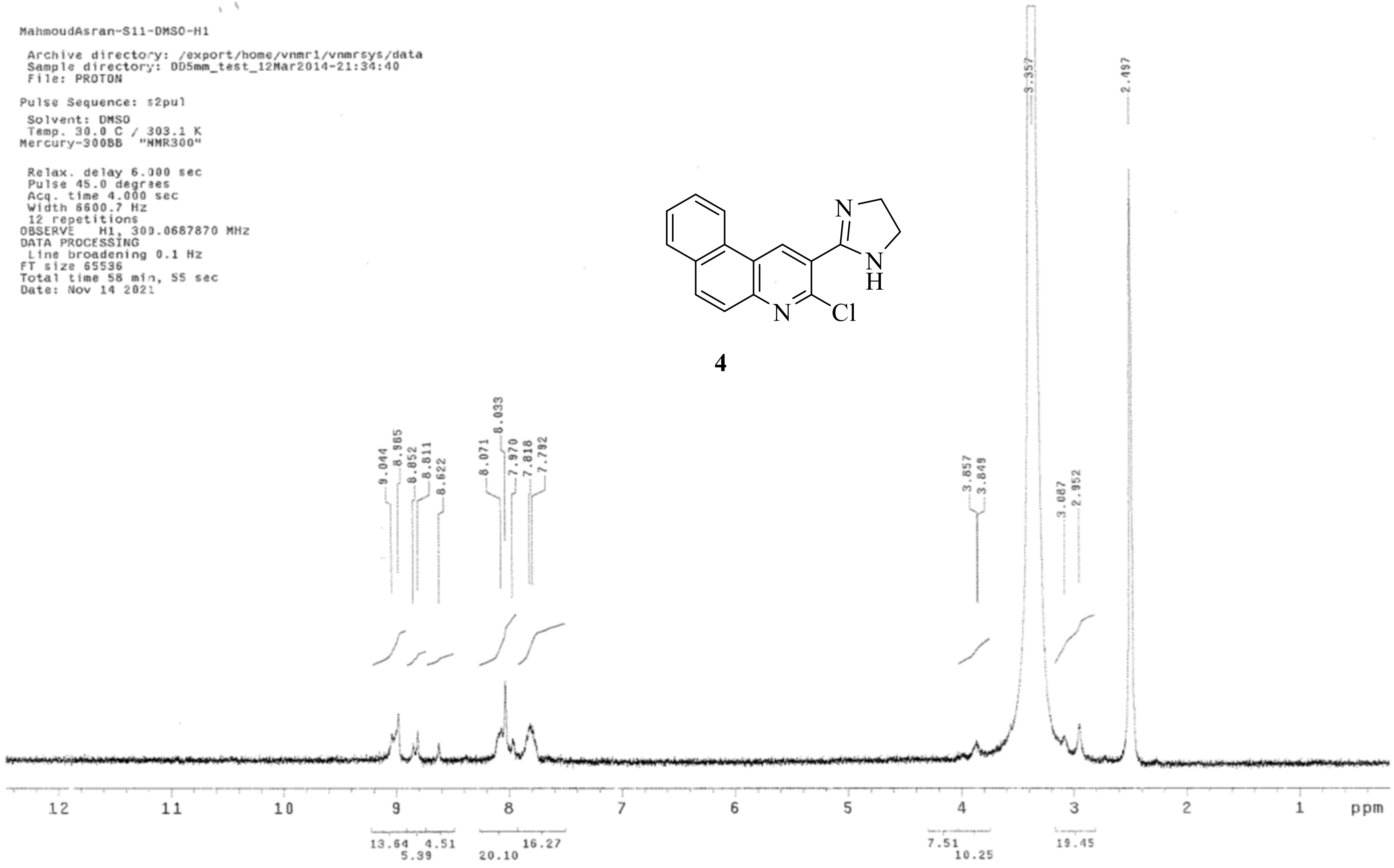
MahmoudAsran-S11-DMSO-H1
Archive directory: /export/home/vnmr1/vnmrsys/data
Sample directory: DD5mm_test_12Mar2014-21:34:40
File: PROTON
Pulse Sequence: s2pul
Solvent: DMSO
Temp. 30.0 C / 303.1 K
Mercury-300BB "NMR300"
Relax. delay 6.000 sec
Pulse 45.0 degrees
Acq. time 4.000 sec
Width 6600.7 Hz
12 repetitions
OBSERVE H1, 300.0687870 MHz
DATA PROCESSING
Line broadening 0.1 Hz
FT size 65536
Total time 58 min, 55 sec
Date: Nov 14 2021
N
N
H
N
Cl
4
9.044
8.985
8.852
8.811
8.622
8.071
8.033
7.970
7.818
7.792
3.857
3.849
3.357
3.087
2.952
2.497
12
11
10
9
8
7
6
5
4
3
2
1
ppm
13.64
4.51
5.39
20.10
16.27
7.51
10.25
19.45

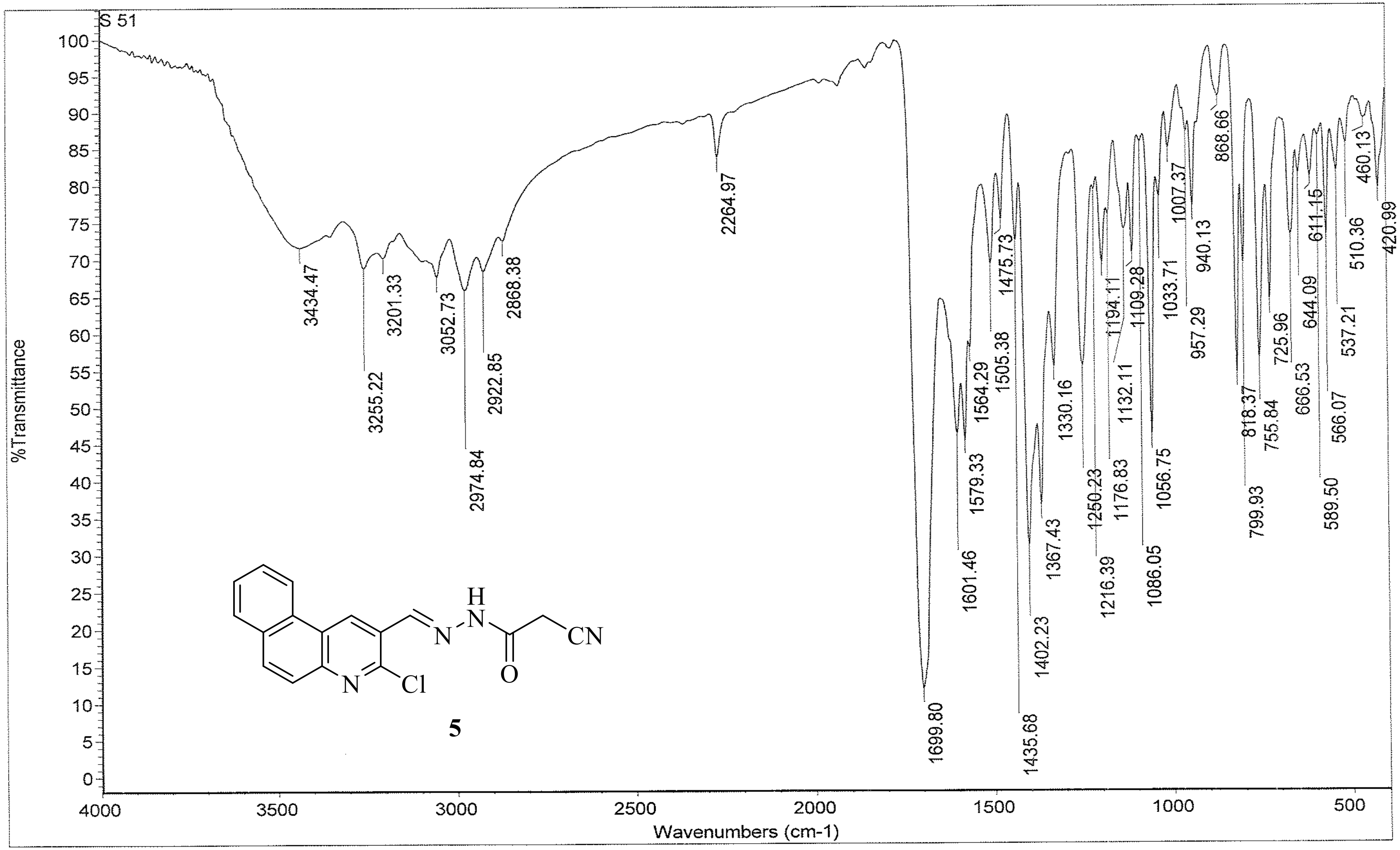
S 51
%Transmittance
Wavenumbers (cm-1)
4000
3500
3000
2500
2000
1500
1000
500
100
95
90
85
80
75
70
65
60
55
50
45
40
35
30
25
20
15
10
5
0
3434.47
3255.22
3201.33
3052.73
2974.84
2922.85
2868.38
2264.97
1699.80
1601.46
1579.33
1564.29
1505.38
1475.73
1435.68
1402.23
1367.43
1330.16
1250.23
1216.39
1194.11
1176.83
1132.11
1109.28
1086.05
1056.75
1033.71
1007.37
957.29
940.13
868.66
818.37
799.93
755.84
725.96
666.53
644.09
611.15
589.50
566.07
537.21
510.36
460.13
420.99
H
N
N
CN
O
N
Cl
5

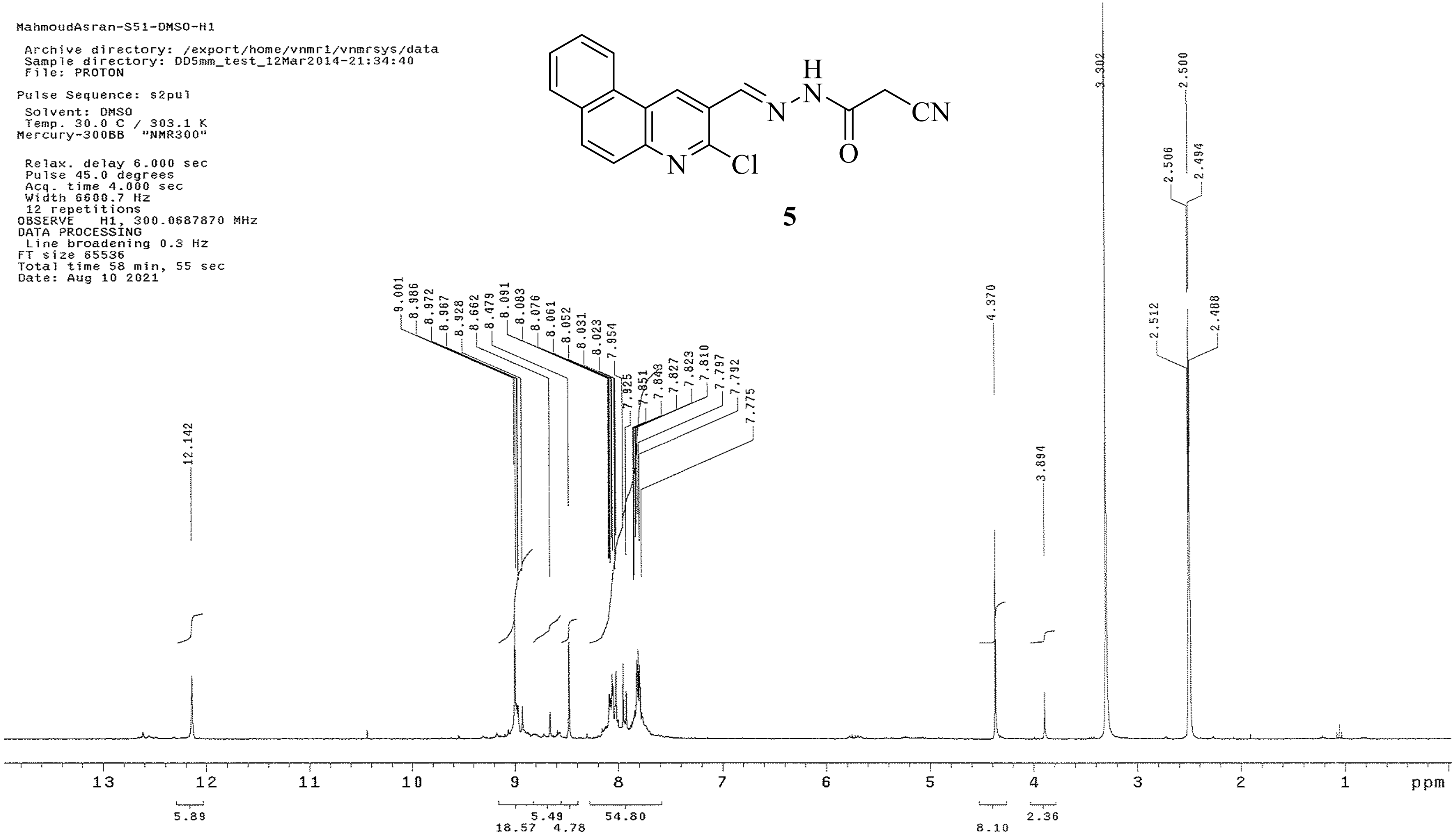
MahmoudAsran-S51-DMSO-H1
Archive directory: /export/home/vnmr1/vnmrsys/data
Sample directory: DD5mm_test_12Mar2014-21:34:40
File: PROTON
Pulse Sequence: s2pul
Solvent: DMSO
Temp. 30.0 C / 303.1 K
Mercury-300BB "NMR300"
Relax. delay 6.000 sec
Pulse 45.0 degrees
Acq. time 4.000 sec
Width 6600.7 Hz
12 repetitions
OBSERVE H1, 300.0687870 MHz
DATA PROCESSING
Line broadening 0.3 Hz
FT size 65536
Total time 58 min, 55 sec
Date: Aug 10 2021
H
N
N
CN
O
N
Cl
12.142
9.001
8.986
8.972
8.967
8.928
8.662
8.479
8.091
8.083
8.076
8.061
8.052
8.031
8.023
7.954
7.925
7.851
7.827
7.823
7.810
7.797
7.792
7.775
4.370
3.894
3.302
2.506
2.500
2.494
2.512
2.488
13
12
11
10
9
8
7
6
5
4
3
2
1
ppm
5.89
5.49
18.57
4.78
54.80
8.10
2.36

5

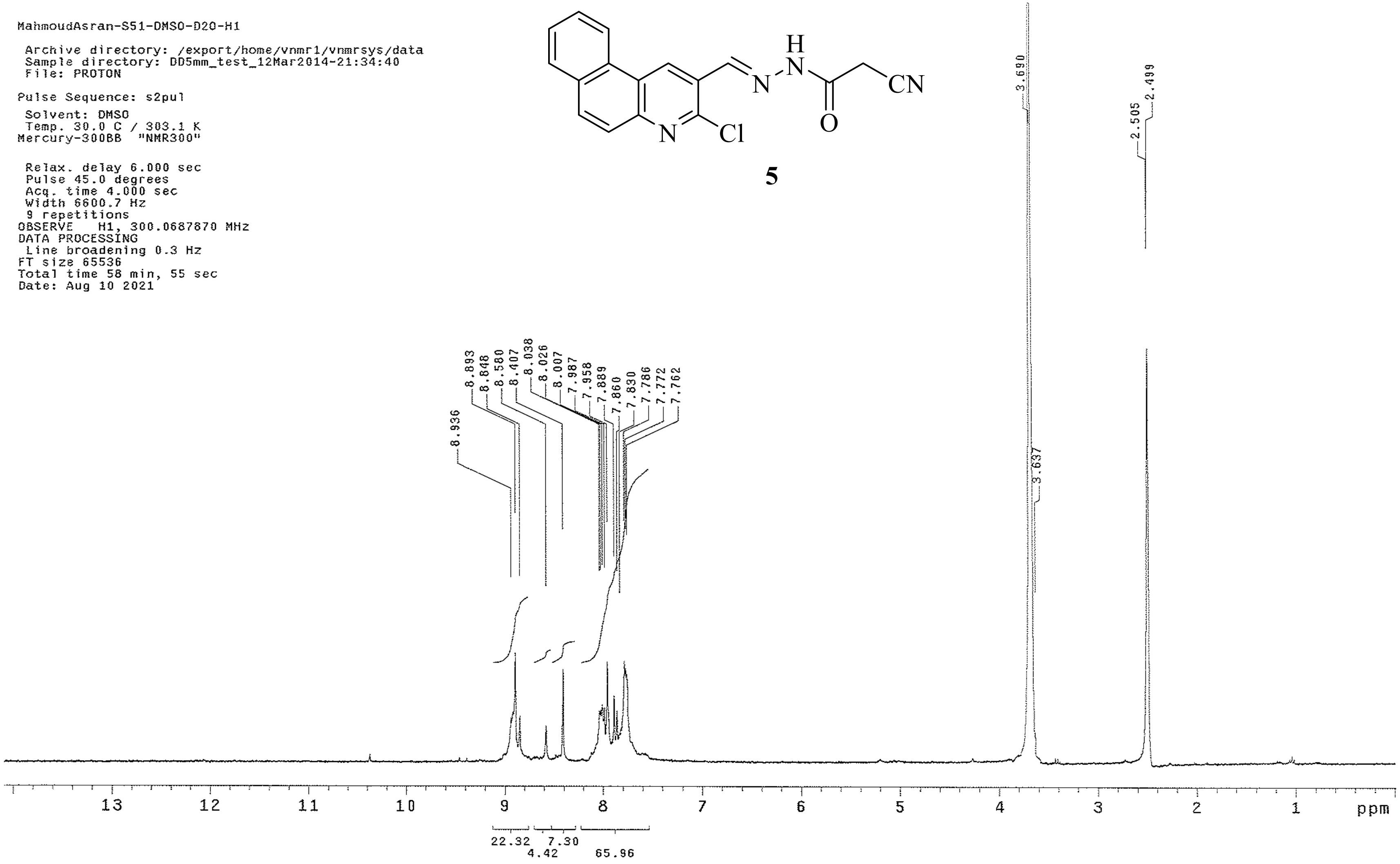
MahmoudAsran-S51-DMSO-D20-H1
Archive directory: /export/home/vnmr1/vnmrsys/data
Sample directory: DD5mm_test_12Mar2014-21:34:40
File: PROTON
Pulse Sequence: s2pul
Solvent: DMSO
Temp. 30.0 C / 303.1 K
Mercury-300BB "NMR300"
Relax. delay 6.000 sec
Pulse 45.0 degrees
Acq. time 4.000 sec
Width 6600.7 Hz
9 repetitions
OBSERVE H1, 300.0687870 MHz
DATA PROCESSING
Line broadening 0.3 Hz
FT size 65536
Total time 58 min, 55 sec
Date: Aug 10 2021
8.936
8.893
8.848
8.580
8.407
8.038
8.026
8.007
7.987
7.958
7.889
7.860
7.830
7.786
7.772
7.762
3.690
3.637
2.505
2.499
13
12
11
10
9
8
7
6
5
4
3
2
1
ppm
22.32
7.30
4.42
65.96
H
N
N
O
CN
N
Cl

5

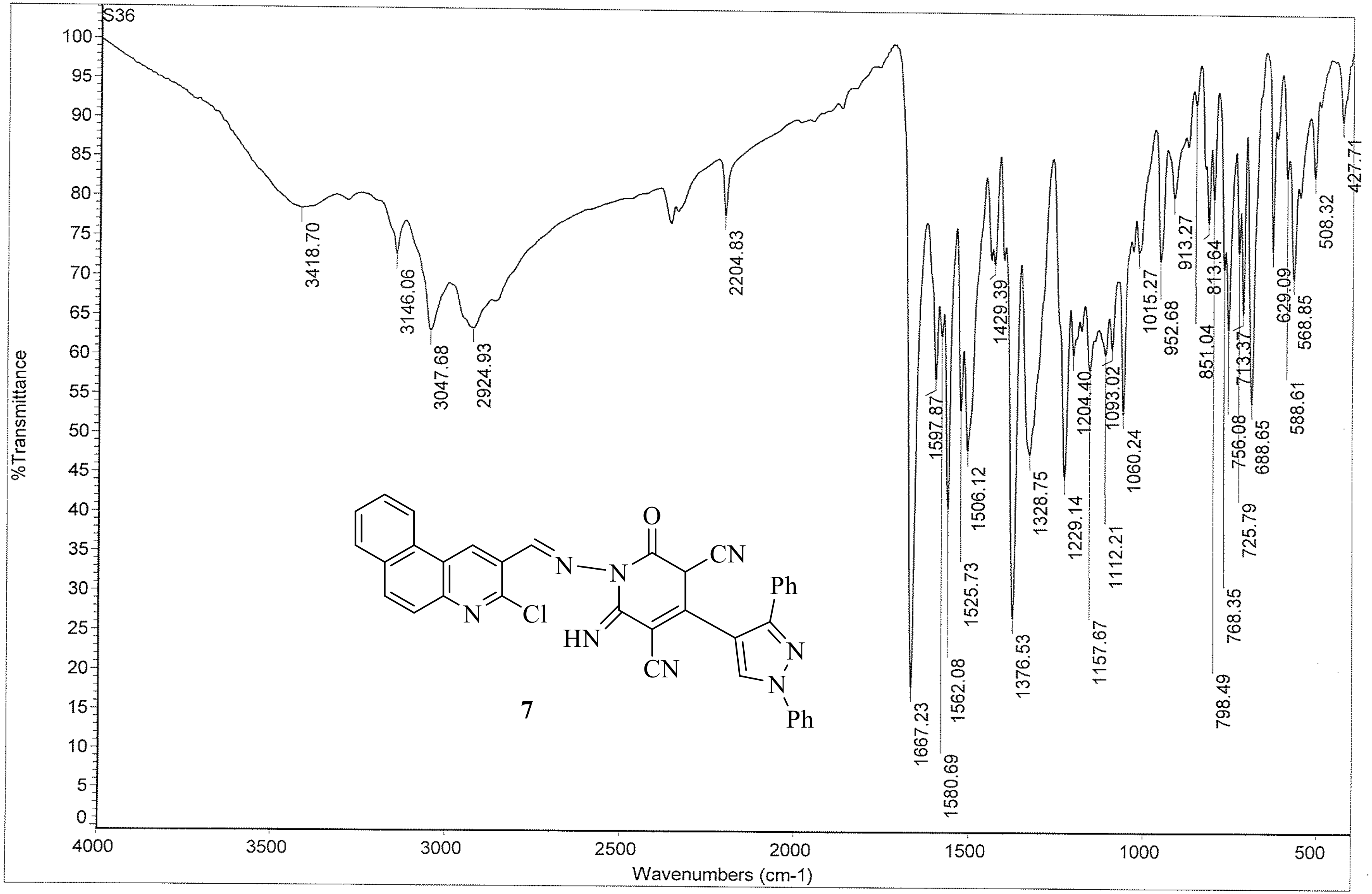
S36
%Transmittance
Wavenumbers (cm-1)
3418.70
3146.06
3047.68
2924.93
2204.83
1667.23
1597.87
1580.69
1562.08
1525.73
1506.12
1429.39
1376.53
1328.75
1229.14
1204.40
1157.67
1112.21
1093.02
1060.24
1015.27
952.68
913.27
851.04
813.64
798.49
768.35
756.08
725.79
713.37
688.65
629.09
588.61
568.85
508.32
427.71
O
CN
N
N
Ph
N
Cl
HN
CN
N
N
Ph
7

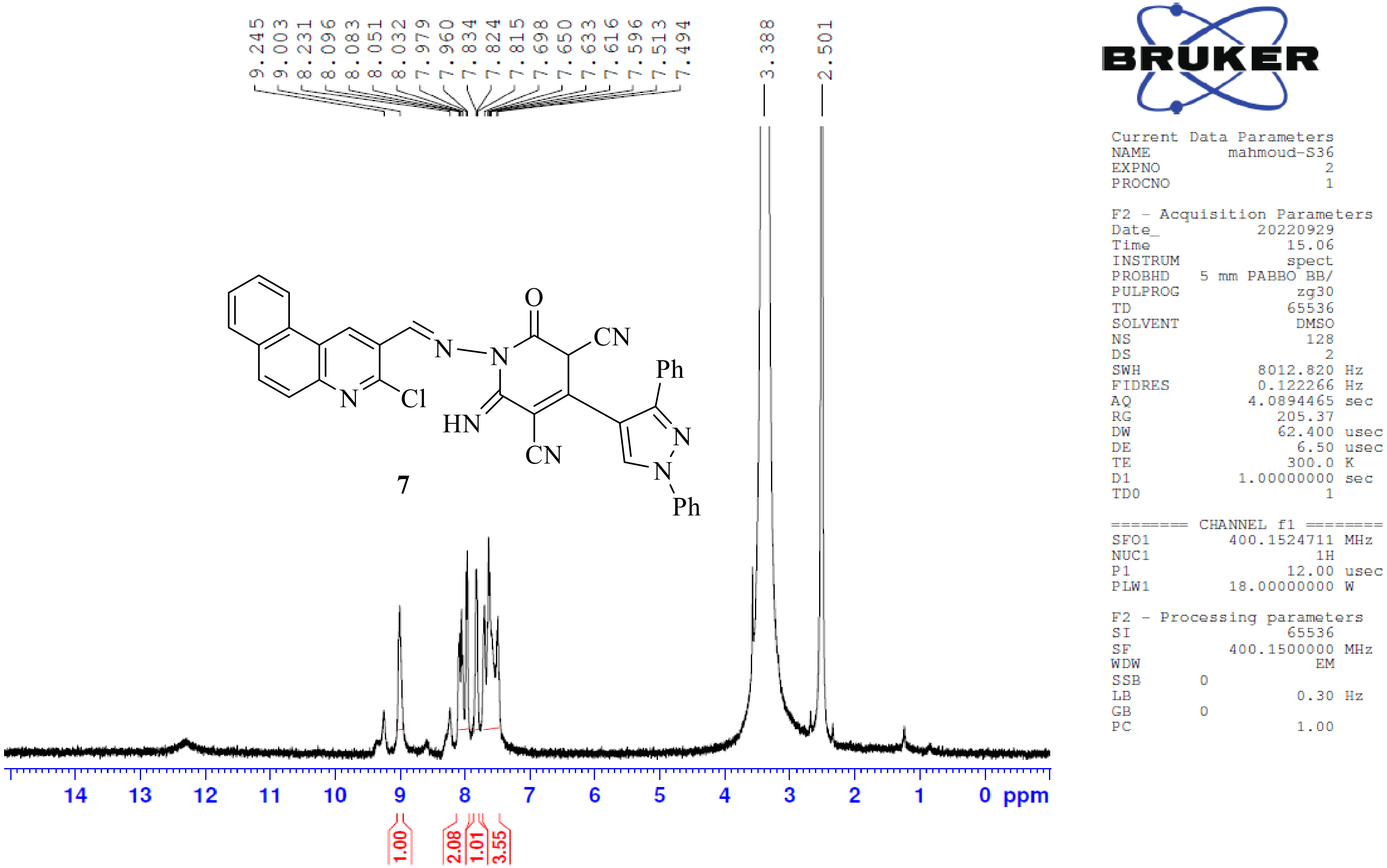
9.245
9.003
8.231
8.096
8.083
8.051
8.032
7.979
7.960
7.834
7.824
7.815
7.698
7.650
7.633
7.616
7.596
7.513
7.494
3.388
2.501
O
CN
N
N
N
Cl
HN
CN
Ph
N
N
Ph
7
14 13 12 11 10 9 8 7 6 5 4 3 2 1 0 ppm
1.00
2.08
1.01
3.55
Current Data Parameters
NAME mahmoud-S36
EXPNO 2
PROCNO 1
F2 - Acquisition Parameters
Date_ 20220929
Time 15.06
INSTRUM spect
PROBHD 5 mm PABBO BB/
PULPROG zg30
TD 65536
SOLVENT DMSO
NS 128
DS 2
SWH 8012.820 Hz
FIDRES 0.122266 Hz
AQ 4.0894465 sec
RG 205.37
DW 62.400 usec
DE 6.50 usec
TE 300.0 K
D1 1.00000000 sec
TD0 1
======== CHANNEL f1 ========
SFO1 400.1524711 MHz
NUC1 1H
P1 12.00 usec
PLW1 18.00000000 W
F2 - Processing parameters
SI 65536
SF 400.1500000 MHz
WDW EM
SSB 0
LB 0.30 Hz
GB 0
PC 1.00

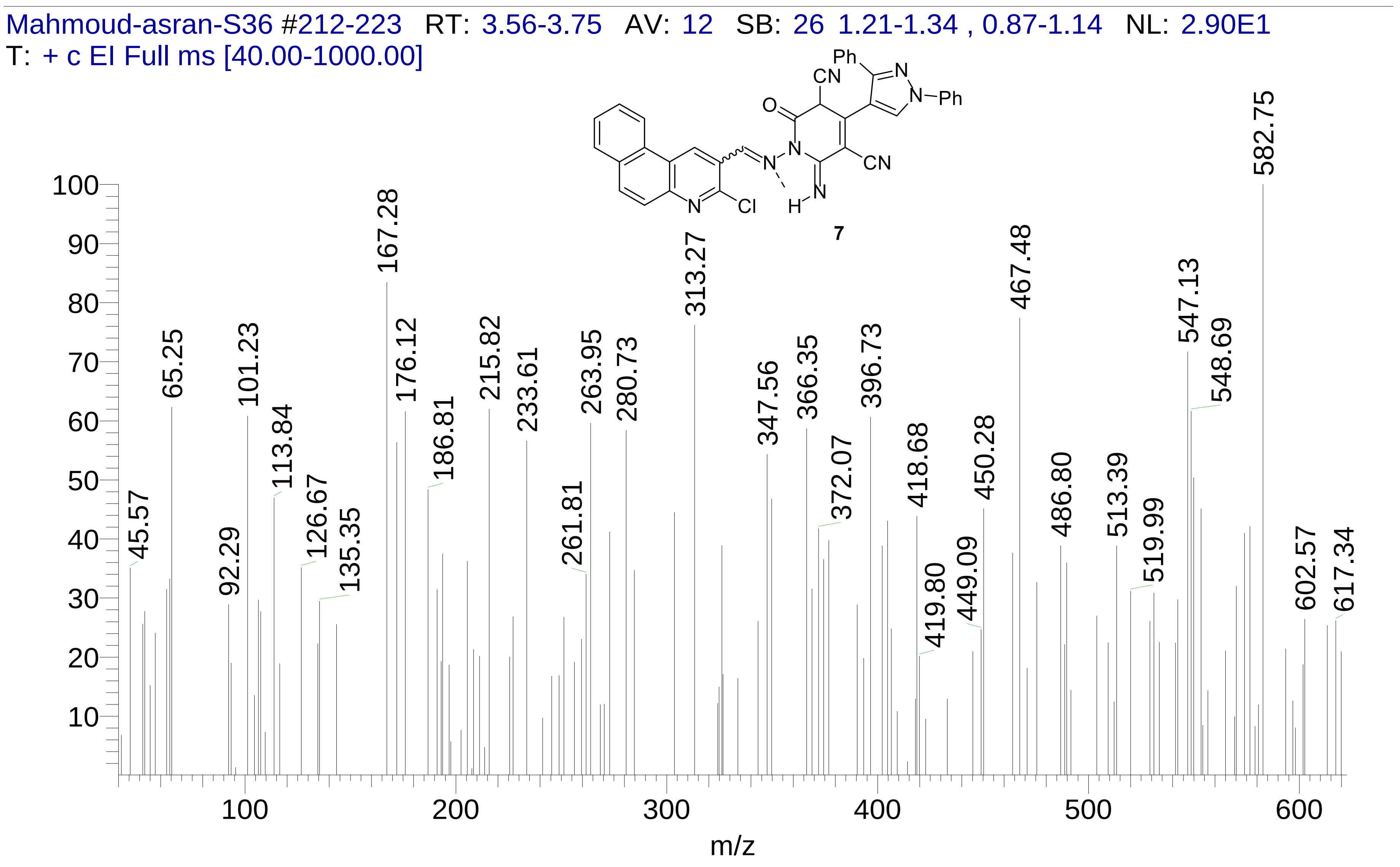
Mahmoud-asran-S36 #212-223 RT: 3.56-3.75 AV: 12 SB: 26 1.21-1.34 , 0.87-1.14 NL: 2.90E1
T: + c EI Full ms [40.00-1000.00]
Ph
CN
N
N
Ph
O
N
N
CN
N
Cl
H
N
7
45.57
65.25
92.29
101.23
113.84
126.67
135.35
167.28
176.12
186.81
215.82
233.61
261.81
263.95
280.73
313.27
347.56
366.35
372.07
396.73
418.68
419.80
449.09
450.28
467.48
486.80
513.39
519.99
547.13
548.69
582.75
602.57
617.34
m/z

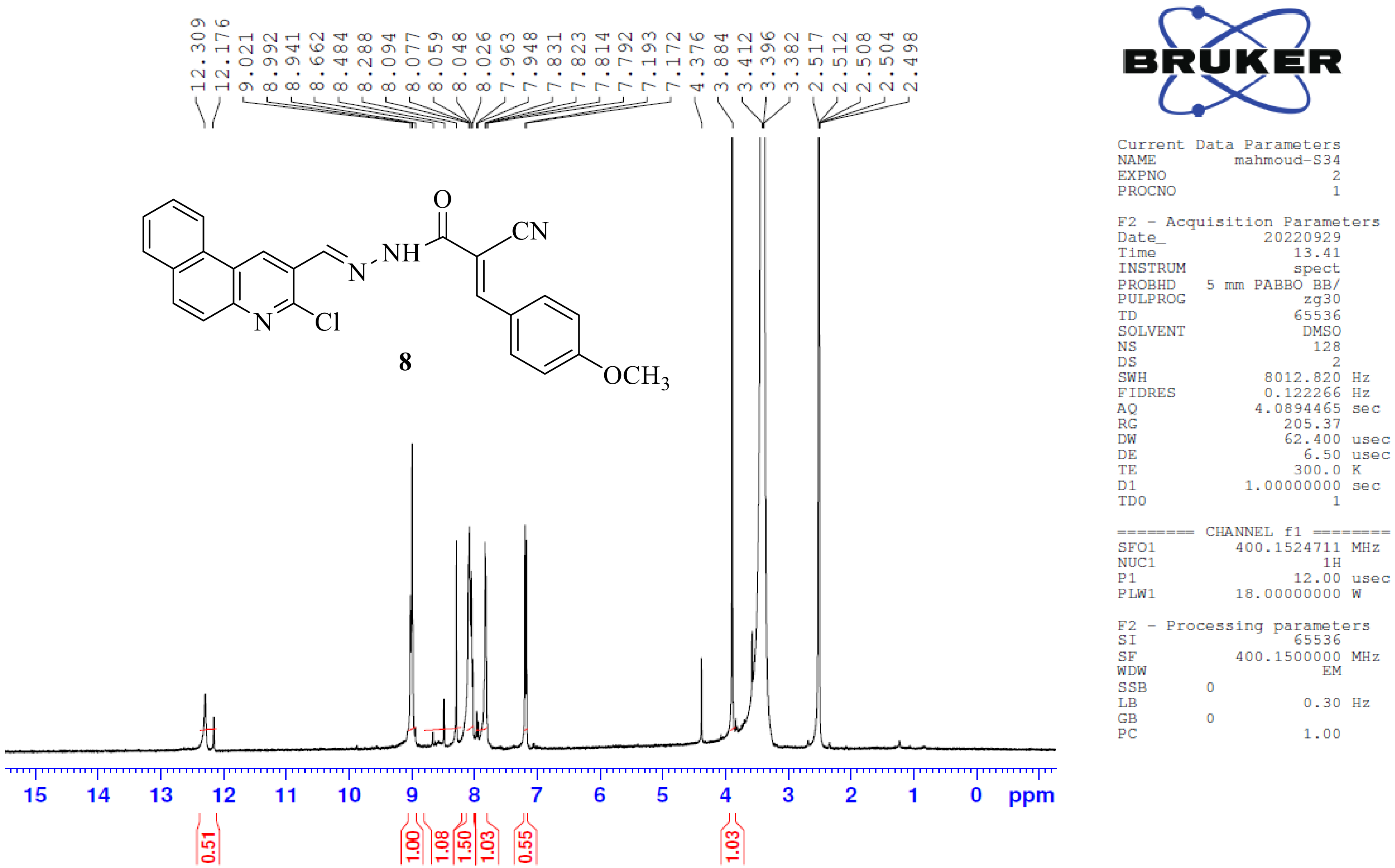
12.309
12.176
9.021
8.992
8.941
8.662
8.484
8.288
8.094
8.077
8.059
8.048
8.026
7.963
7.948
7.831
7.823
7.814
7.792
7.193
7.172
4.376
3.884
3.412
3.396
3.382
2.517
2.512
2.508
2.504
2.498
O
CN
NH
N
N
Cl
8
OCH3
15 14 13 12 11 10 9 8 7 6 5 4 3 2 1 0 ppm
0.51
1.00
1.08
1.50
1.03
0.55
1.03
BRUKER
Current Data Parameters
NAME mahmoud-S34
EXPNO 2
PROCNO 1
F2 - Acquisition Parameters
Date_ 20220929
Time 13.41
INSTRUM spect
PROBHD 5 mm PABBO BB/
PULPROG zg30
TD 65536
SOLVENT DMSO
NS 128
DS 2
SWH 8012.820 Hz
FIDRES 0.122266 Hz
AQ 4.0894465 sec
RG 205.37
DW 62.400 usec
DE 6.50 usec
TE 300.0 K
D1 1.00000000 sec
TD0 1
======== CHANNEL f1 ========
SFO1 400.1524711 MHz
NUC1 1H
P1 12.00 usec
PLW1 18.00000000 W
F2 - Processing parameters
SI 65536
SF 400.1500000 MHz
WDW EM
SSB 0
LB 0.30 Hz
GB 0
PC 1.00

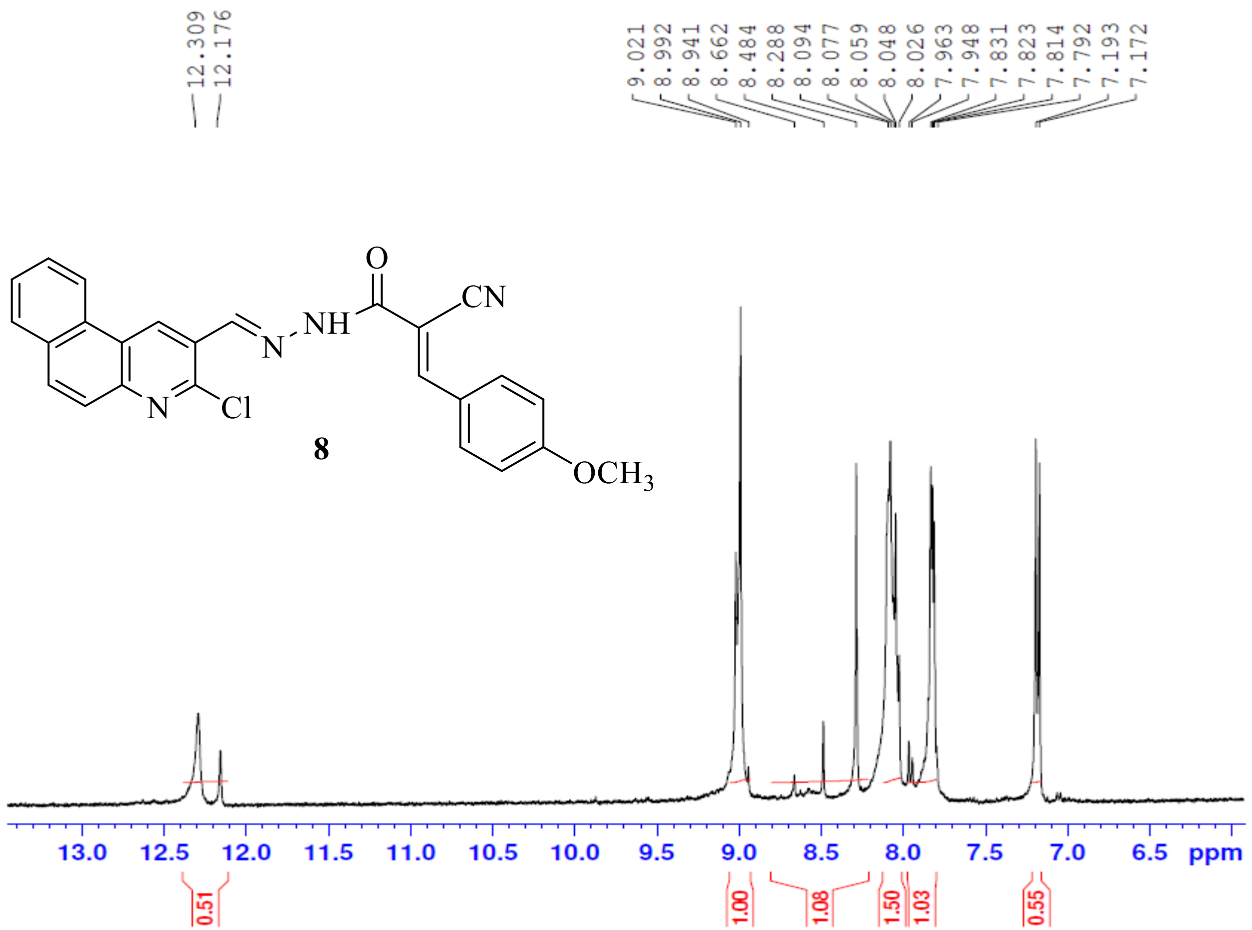


```
Current Data Parameters
NAME          mahmoud-S34
EXPNO                   2
PROCNO                  1

F2 - Acquisition Parameters
Date_            20220929
Time                13.41
INSTRUM             spect
PROBHD   5 mm PABBO BB/
PULPROG              zg30
TD                  65536
SOLVENT              DMSO
NS                    128
DS                      2
SWH              8012.820 Hz
FIDRES           0.122266 Hz
AQ              4.0894465 sec
RG                 205.37
DW                 62.400 usec
DE                   6.50 usec
TE                  300.0 K
D1             1.00000000 sec
TD0                     1

======== CHANNEL f1 ========
SFO1          400.1524711 MHz
NUC1                   1H
P1                  12.00 usec
PLW1          18.00000000 W

F2 - Processing parameters
SI                  65536
SF            400.1500000 MHz
WDW                    EM
SSB      0
LB                   0.30 Hz
GB       0
PC                   1.00
```

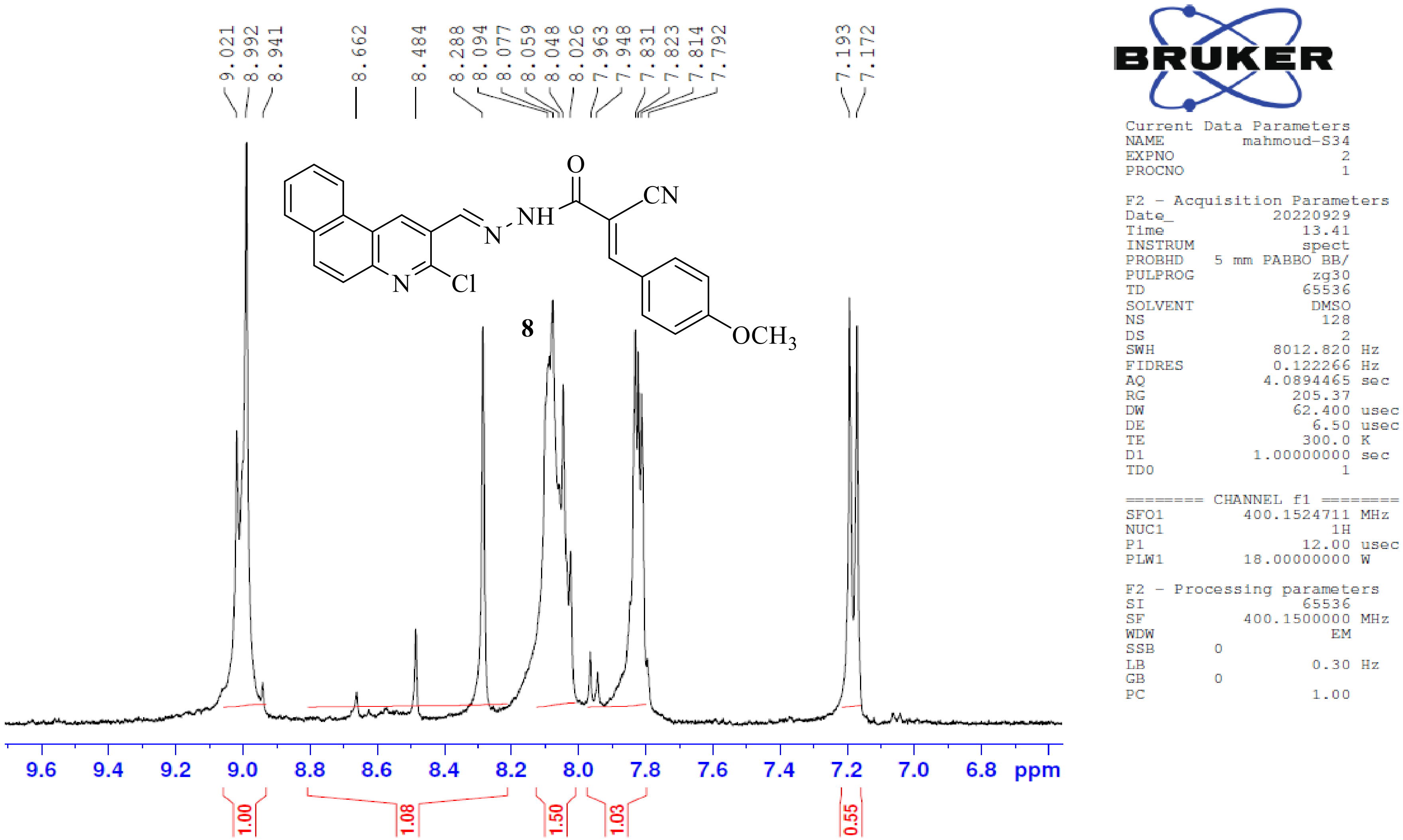
9.021
8.992
8.941
8.662
8.484
8.288
8.094
8.077
8.059
8.048
8.026
7.963
7.948
7.831
7.823
7.814
7.792
7.193
7.172
O
CN
N
NH
N
Cl
OCH3
8
9.6 9.4 9.2 9.0 8.8 8.6 8.4 8.2 8.0 7.8 7.6 7.4 7.2 7.0 6.8 ppm
1.00
1.08
1.50
1.03
0.55
BRUKER
Current Data Parameters
NAME mahmoud-S34
EXPNO 2
PROCNO 1
F2 - Acquisition Parameters
Date_ 20220929
Time 13.41
INSTRUM spect
PROBHD 5 mm PABBO BB/
PULPROG zg30
TD 65536
SOLVENT DMSO
NS 128
DS 2
SWH 8012.820 Hz
FIDRES 0.122266 Hz
AQ 4.0894465 sec
RG 205.37
DW 62.400 usec
DE 6.50 usec
TE 300.0 K
D1 1.00000000 sec
TD0 1
======== CHANNEL f1 ========
SFO1 400.1524711 MHz
NUC1 1H
P1 12.00 usec
PLW1 18.00000000 W
F2 - Processing parameters
SI 65536
SF 400.1500000 MHz
WDW EM
SSB 0
LB 0.30 Hz
GB 0
PC 1.00

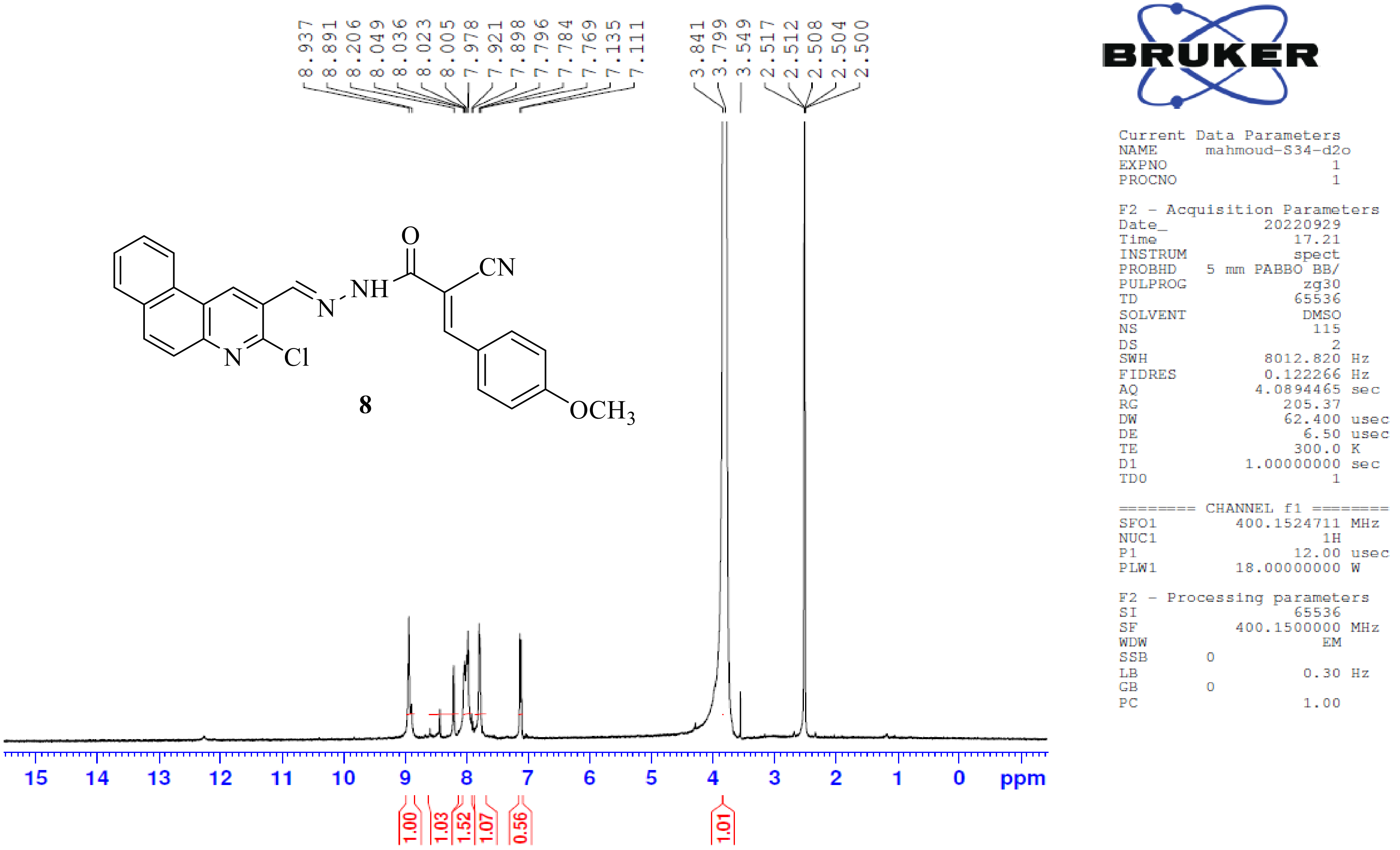
8.937
8.891
8.206
8.049
8.036
8.023
8.005
7.978
7.921
7.898
7.796
7.784
7.769
7.135
7.111
3.841
3.799
3.549
2.517
2.512
2.508
2.504
2.500
O
NH
CN
N
N
Cl
8
OCH_3
BRUKER
Current Data Parameters
NAME mahmoud-S34-d2o
EXPNO 1
PROCNO 1
F2 - Acquisition Parameters
Date_ 20220929
Time 17.21
INSTRUM spect
PROBHD 5 mm PABBO BB/
PULPROG zg30
TD 65536
SOLVENT DMSO
NS 115
DS 2
SWH 8012.820 Hz
FIDRES 0.122266 Hz
AQ 4.0894465 sec
RG 205.37
DW 62.400 usec
DE 6.50 usec
TE 300.0 K
D1 1.00000000 sec
TD0 1
======== CHANNEL f1 ========
SFO1 400.1524711 MHz
NUC1 1H
P1 12.00 usec
PLW1 18.00000000 W
F2 - Processing parameters
SI 65536
SF 400.1500000 MHz
WDW EM
SSB 0
LB 0.30 Hz
GB 0
PC 1.00
15 14 13 12 11 10 9 8 7 6 5 4 3 2 1 0 ppm
1.00
1.03
1.52
1.07
0.56
1.01

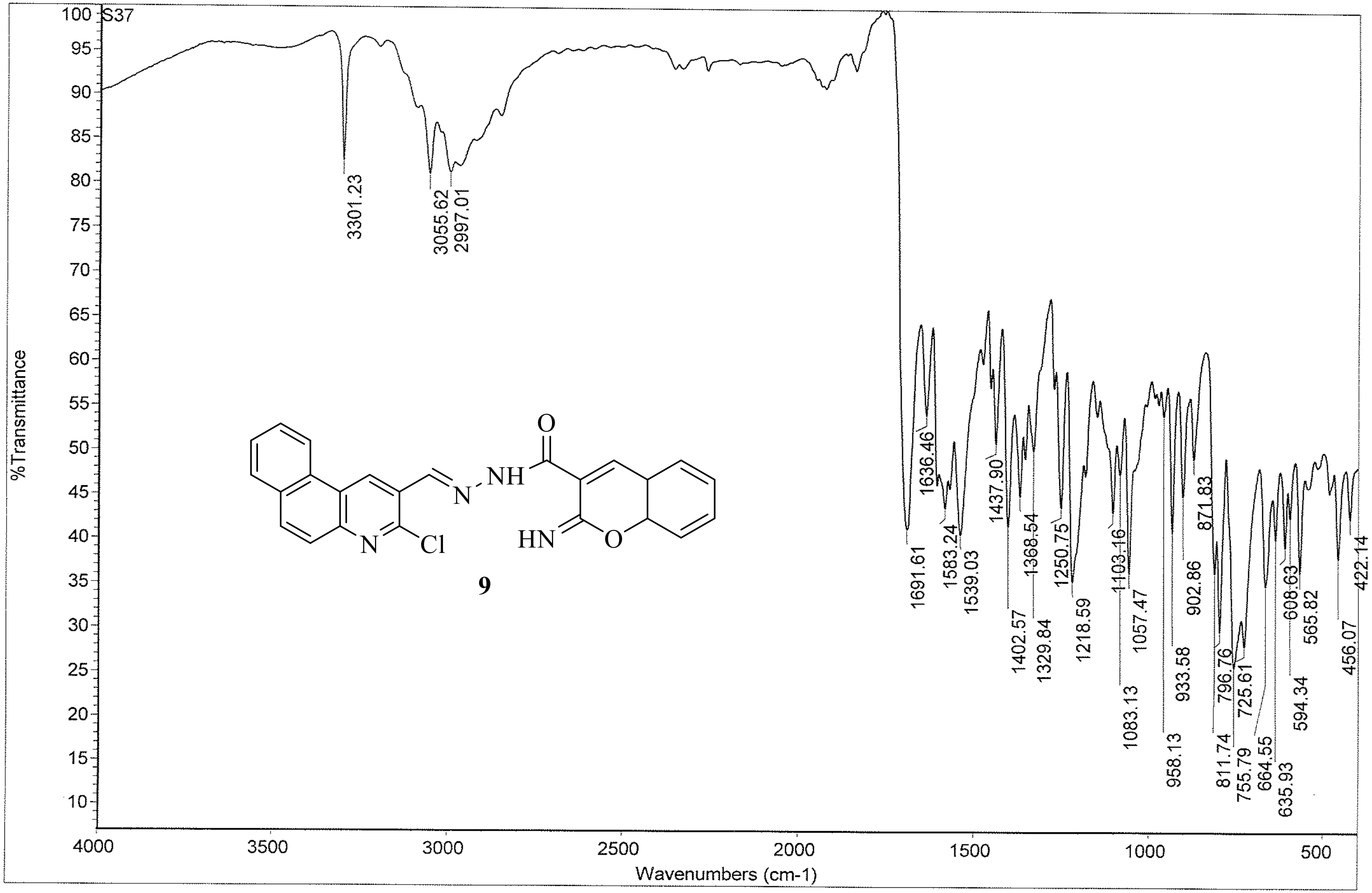
S37
%Transmittance
Wavenumbers (cm-1)
4000
3500
3000
2500
2000
1500
1000
500
100
95
90
85
80
75
70
65
60
55
50
45
40
35
30
25
20
15
10
3301.23
3055.62
2997.01
1691.61
1636.46
1583.24
1539.03
1437.90
1402.57
1368.54
1329.84
1250.75
1218.59
1103.16
1083.13
1057.47
958.13
933.58
902.86
871.83
811.74
796.76
755.79
725.61
664.55
635.93
608.63
594.34
565.82
456.07
422.14
O
NH
N
N
Cl
HN
O
9

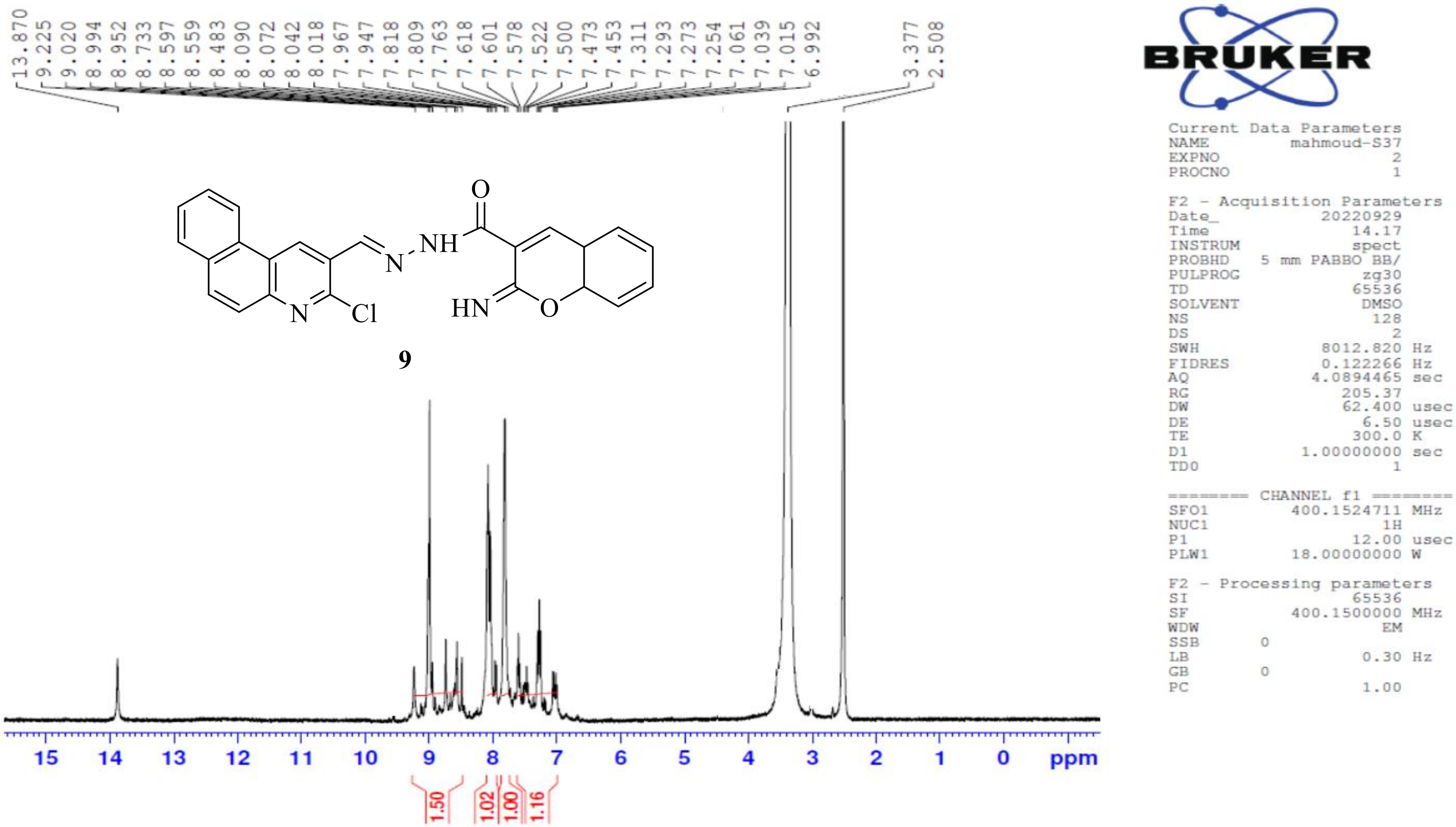
13.870
9.225
9.020
8.994
8.952
8.733
8.597
8.559
8.483
8.090
8.072
8.042
8.018
7.967
7.947
7.818
7.809
7.763
7.618
7.601
7.578
7.522
7.500
7.473
7.453
7.311
7.293
7.273
7.254
7.061
7.039
7.015
6.992
3.377
2.508
O
NH
N
N
Cl
HN
O
9
15 14 13 12 11 10 9 8 7 6 5 4 3 2 1 0 ppm
1.50
1.02
1.00
1.16
BRUKER
Current Data Parameters
NAME mahmoud-S37
EXPNO 2
PROCNO 1
F2 - Acquisition Parameters
Date_ 20220929
Time 14.17
INSTRUM spect
PROBHD 5 mm PABBO BB/
PULPROG zg30
TD 65536
SOLVENT DMSO
NS 128
DS 2
SWH 8012.820 Hz
FIDRES 0.122266 Hz
AQ 4.0894465 sec
RG 205.37
DW 62.400 usec
DE 6.50 usec
TE 300.0 K
D1 1.00000000 sec
TD0 1
======== CHANNEL f1 ========
SFO1 400.1524711 MHz
NUC1 1H
P1 12.00 usec
PLW1 18.00000000 W
F2 - Processing parameters
SI 65536
SF 400.1500000 MHz
WDW EM
SSB 0
LB 0.30 Hz
GB 0
PC 1.00

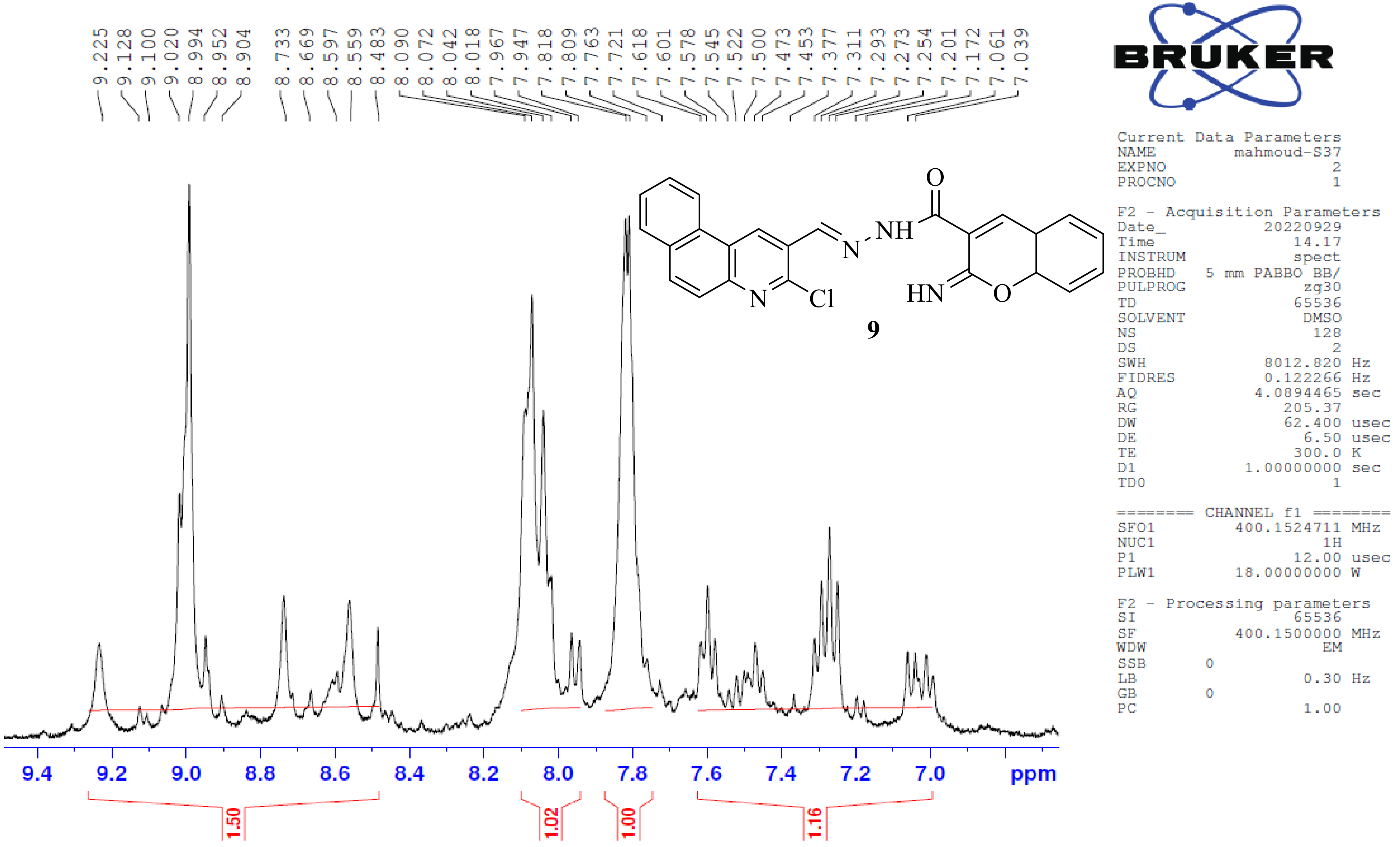
9.225
9.128
9.100
9.020
8.994
8.952
8.904
8.733
8.669
8.597
8.559
8.483
8.090
8.072
8.042
8.018
7.967
7.947
7.818
7.809
7.763
7.721
7.618
7.601
7.578
7.545
7.522
7.500
7.473
7.453
7.377
7.311
7.293
7.273
7.254
7.201
7.172
7.061
7.039
O
NH
N
HN
O
N
Cl
9
BRUKER
Current Data Parameters
NAME mahmoud-S37
EXPNO 2
PROCNO 1
F2 - Acquisition Parameters
Date_ 20220929
Time 14.17
INSTRUM spect
PROBHD 5 mm PABBO BB/
PULPROG zg30
TD 65536
SOLVENT DMSO
NS 128
DS 2
SWH 8012.820 Hz
FIDRES 0.122266 Hz
AQ 4.0894465 sec
RG 205.37
DW 62.400 usec
DE 6.50 usec
TE 300.0 K
D1 1.00000000 sec
TD0 1
======== CHANNEL f1 ========
SFO1 400.1524711 MHz
NUC1 1H
P1 12.00 usec
PLW1 18.00000000 W
F2 - Processing parameters
SI 65536
SF 400.1500000 MHz
WDW EM
SSB 0
LB 0.30 Hz
GB 0
PC 1.00
9.4
9.2
9.0
8.8
8.6
8.4
8.2
8.0
7.8
7.6
7.4
7.2
7.0
ppm
1.50
1.02
1.00
1.16

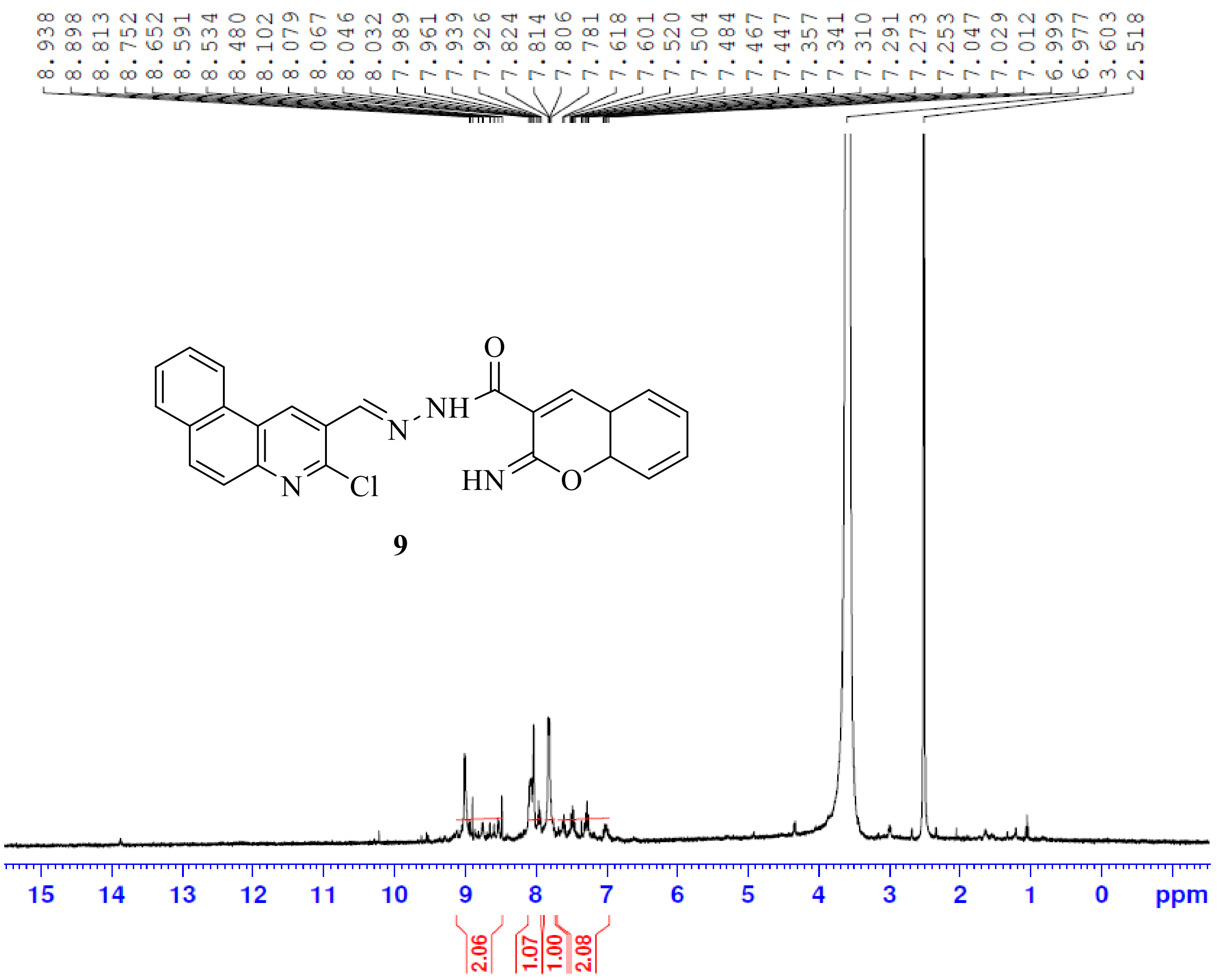

Current Data Parameters
NAME mahmoud-S37-d20
EXPNO 3
PROCNO 1

F2 - Acquisition Parameters
Date_ 20221009
Time 14.21
INSTRUM spect
PROBHD 5 mm PABBO BB/
PULPROG zg30
TD 65536
SOLVENT DMSO
NS 128
DS 2
SWH 8012.820 Hz
FIDRES 0.122266 Hz
AQ 4.0894465 sec
RG 205.37
DW 62.400 usec
DE 6.50 usec
TE 300.0 K
D1 1.00000000 sec
TD0 1

======== CHANNEL f1 ========
SFO1 400.1524711 MHz
NUC1 1H
P1 12.00 usec
PLW1 18.00000000 W

F2 - Processing parameters
SI 65536
SF 400.1500000 MHz
WDW EM
SSB 0
LB 0.30 Hz
GB 0
PC 1.00

Mahmoud-asran-S37 #164-166 RT: 2.76-2.79 AV: 3 SB: 26 1.21-1.34 , 0.87-1.14 NL: 6.85E1
T: + c EI Full ms [40.00-1000.00]

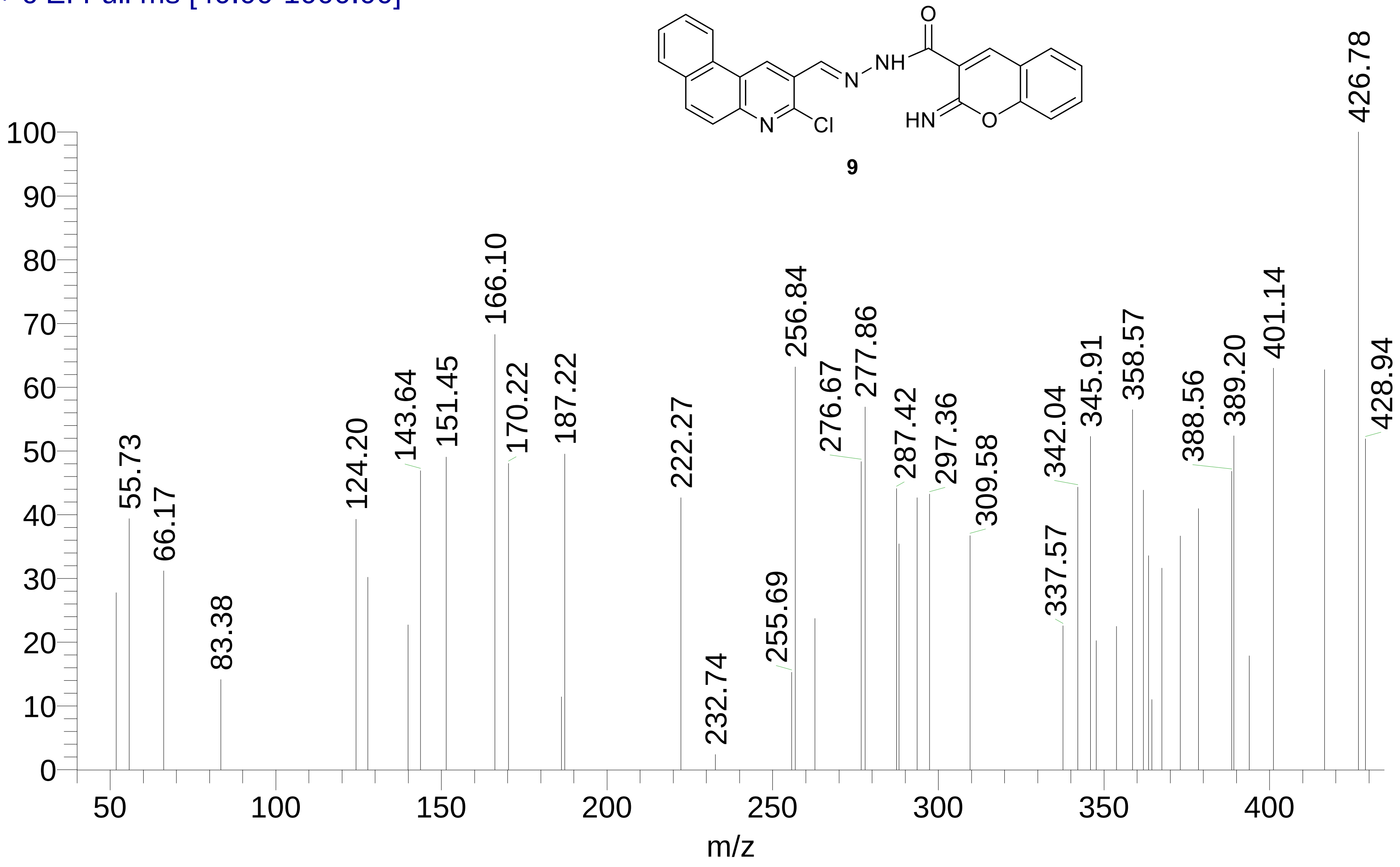

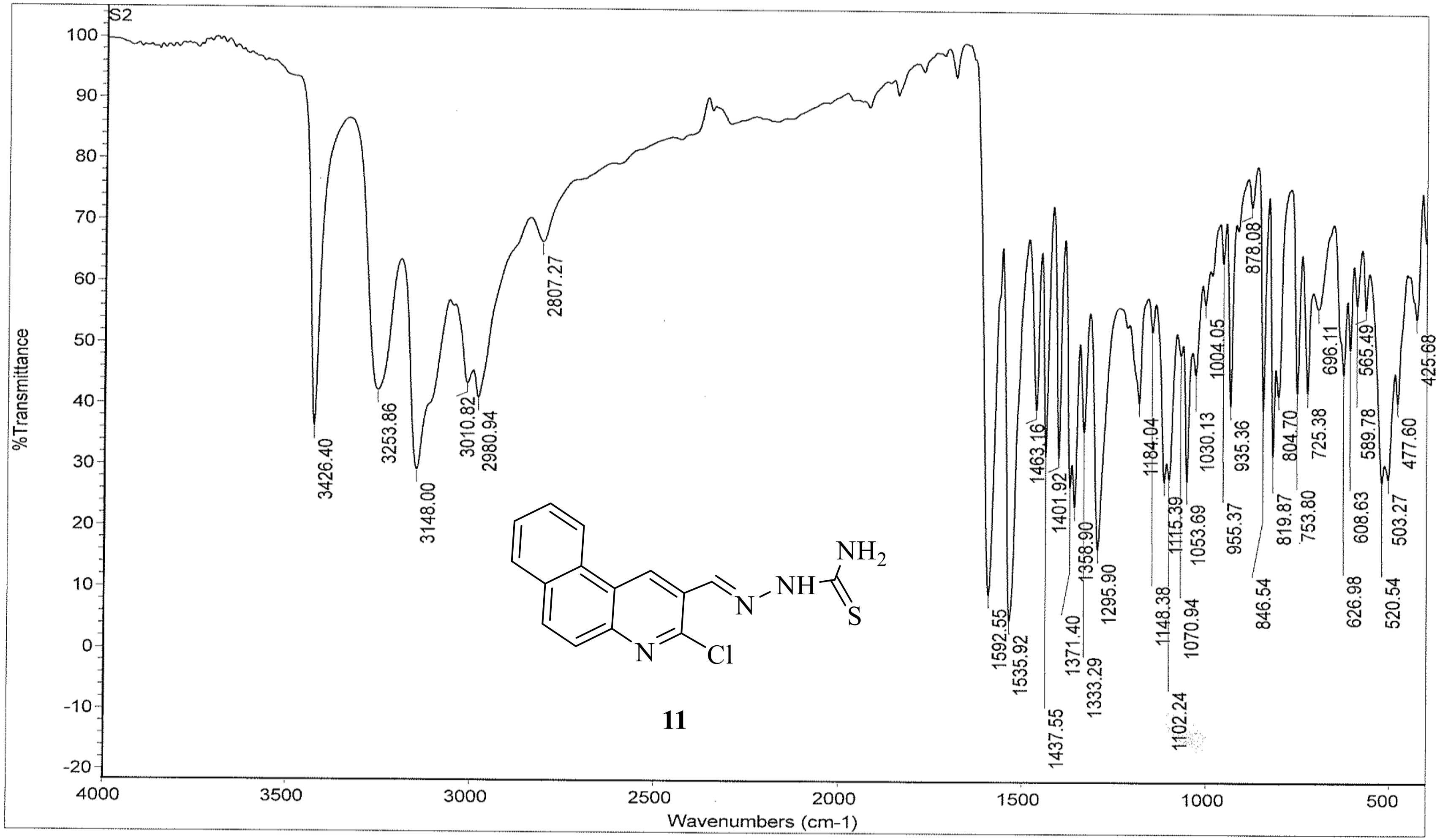
S2
%Transmittance
Wavenumbers (cm-1)
3426.40
3253.86
3148.00
3010.82
2980.94
2807.27
1592.55
1535.92
1463.16
1437.55
1401.92
1371.40
1358.90
1333.29
1295.90
1184.04
1148.38
1115.39
1102.24
1070.94
1053.69
1030.13
1004.05
955.37
935.36
878.08
846.54
819.87
804.70
753.80
725.38
696.11
626.98
608.63
589.78
565.49
520.54
503.27
477.60
425.68
NH2
NH
N
S
N
Cl
11

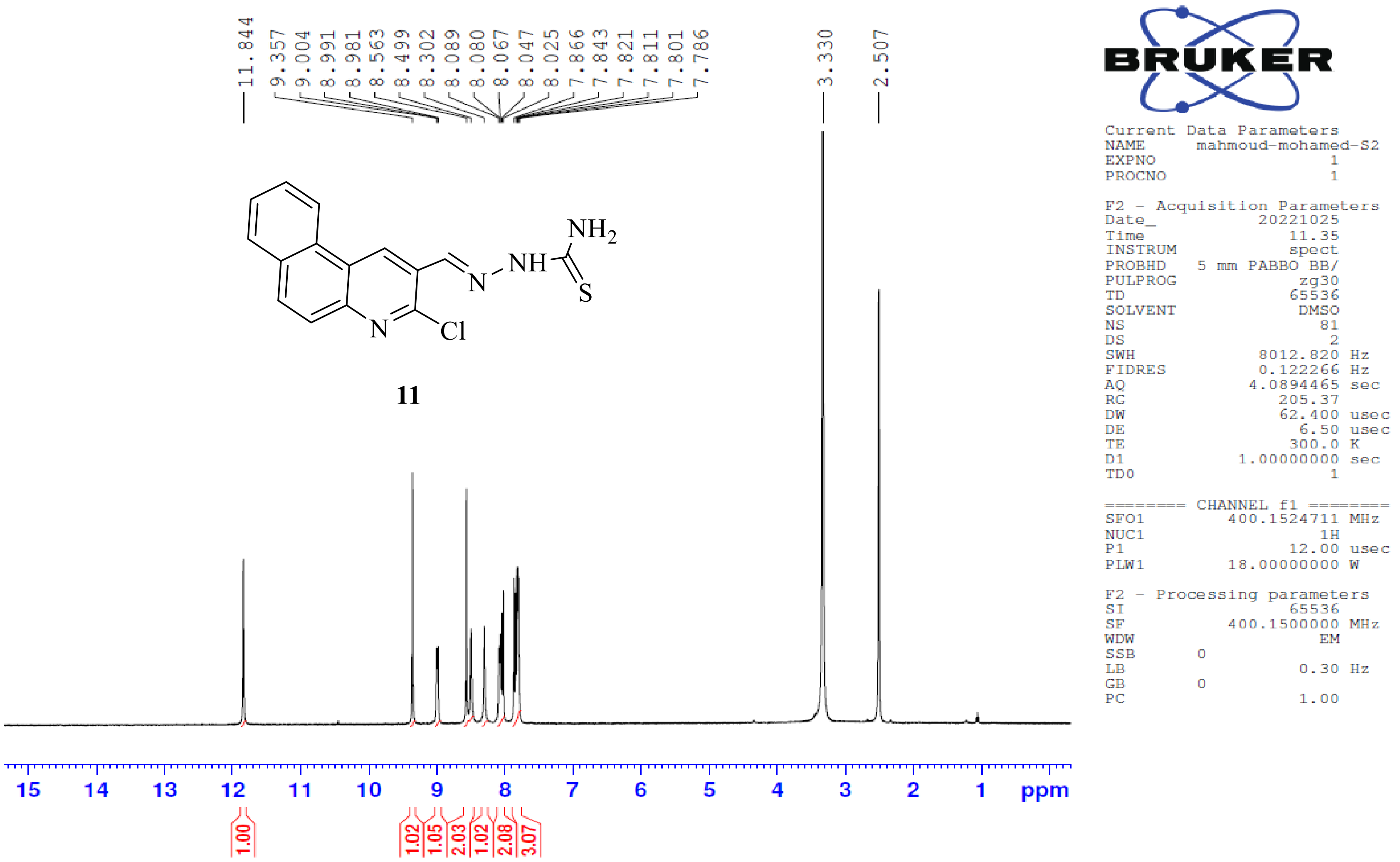
11.844
9.357
9.004
8.991
8.981
8.563
8.499
8.302
8.089
8.080
8.067
8.047
8.025
7.866
7.843
7.821
7.811
7.801
7.786
3.330
2.507
NH2
NH
N
S
N
Cl
11
15 14 13 12 11 10 9 8 7 6 5 4 3 2 1 ppm
1.00
1.02
1.05
2.03
1.02
2.08
3.07
Current Data Parameters
NAME mahmoud-mohamed-S2
EXPNO 1
PROCNO 1
F2 - Acquisition Parameters
Date_ 20221025
Time 11.35
INSTRUM spect
PROBHD 5 mm PABBO BB/
PULPROG zg30
TD 65536
SOLVENT DMSO
NS 81
DS 2
SWH 8012.820 Hz
FIDRES 0.122266 Hz
AQ 4.0894465 sec
RG 205.37
DW 62.400 usec
DE 6.50 usec
TE 300.0 K
D1 1.00000000 sec
TD0 1
======== CHANNEL f1 ========
SFO1 400.1524711 MHz
NUC1 1H
P1 12.00 usec
PLW1 18.00000000 W
F2 - Processing parameters
SI 65536
SF 400.1500000 MHz
WDW EM
SSB 0
LB 0.30 Hz
GB 0
PC 1.00

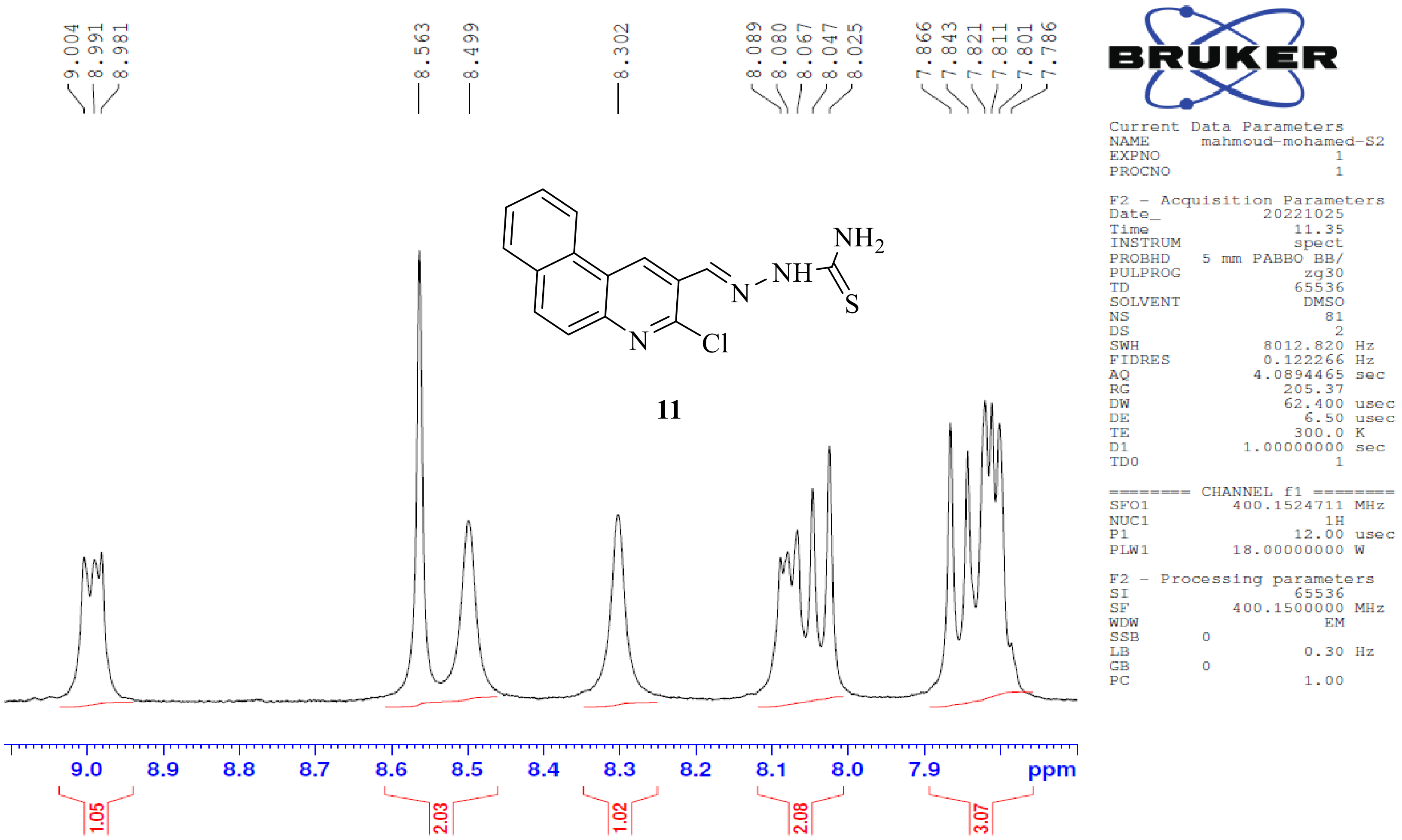
9.004
8.991
8.981
8.563
8.499
8.302
8.089
8.080
8.067
8.047
8.025
7.866
7.843
7.821
7.811
7.801
7.786
NH2
NH
N
S
N
Cl
11
9.0
8.9
8.8
8.7
8.6
8.5
8.4
8.3
8.2
8.1
8.0
7.9
ppm
1.05
2.03
1.02
2.08
3.07
BRUKER
Current Data Parameters
NAME mahmoud-mohamed-S2
EXPNO 1
PROCNO 1
F2 - Acquisition Parameters
Date_ 20221025
Time 11.35
INSTRUM spect
PROBHD 5 mm PABBO BB/
PULPROG zg30
TD 65536
SOLVENT DMSO
NS 81
DS 2
SWH 8012.820 Hz
FIDRES 0.122266 Hz
AQ 4.0894465 sec
RG 205.37
DW 62.400 usec
DE 6.50 usec
TE 300.0 K
D1 1.00000000 sec
TD0 1
======== CHANNEL f1 ========
SFO1 400.1524711 MHz
NUC1 1H
P1 12.00 usec
PLW1 18.00000000 W
F2 - Processing parameters
SI 65536
SF 400.1500000 MHz
WDW EM
SSB 0
LB 0.30 Hz
GB 0
PC 1.00

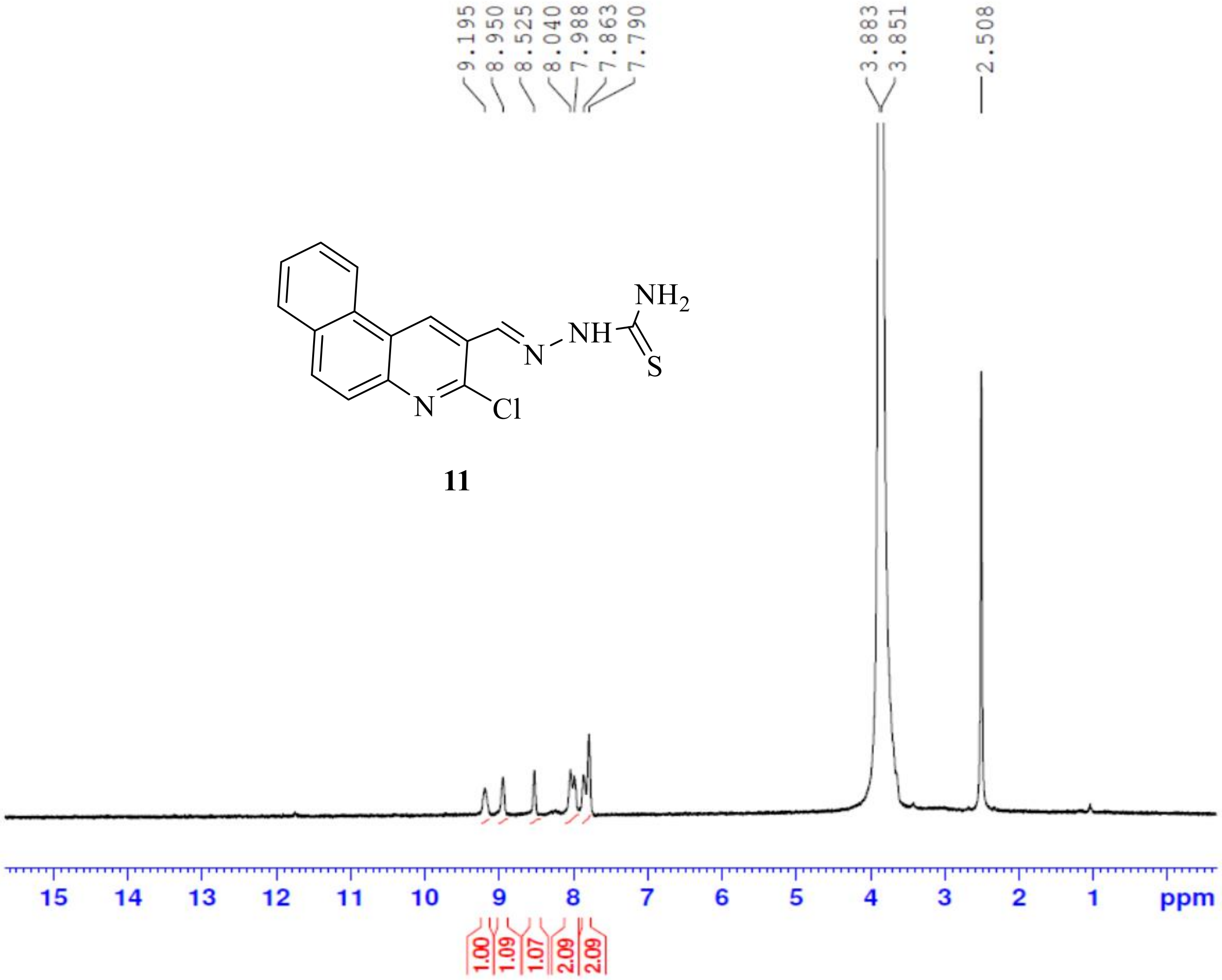

11

Current Data Parameters
NAME mahmoud-mohamed-S2-d2o
EXPNO 1
PROCNO 1

F2 - Acquisition Parameters
Date_ 20221026
Time 10.47
INSTRUM spect
PROBHD 5 mm PABBO BB/
PULPROG zg30
TD 65536
SOLVENT DMSO
NS 80
DS 2
SWH 8012.820 Hz
FIDRES 0.122266 Hz
AQ 4.0894465 sec
RG 205.37
DW 62.400 usec
DE 6.50 usec
TE 300.0 K
D1 1.00000000 sec
TD0 1

======== CHANNEL f1 ========
SFO1 400.1524711 MHz
NUC1 1H
P1 12.00 usec
PLW1 18.00000000 W

F2 - Processing parameters
SI 65536
SF 400.1500000 MHz
WDW EM
SSB 0
LB 0.30 Hz
GB 0
PC 1.00

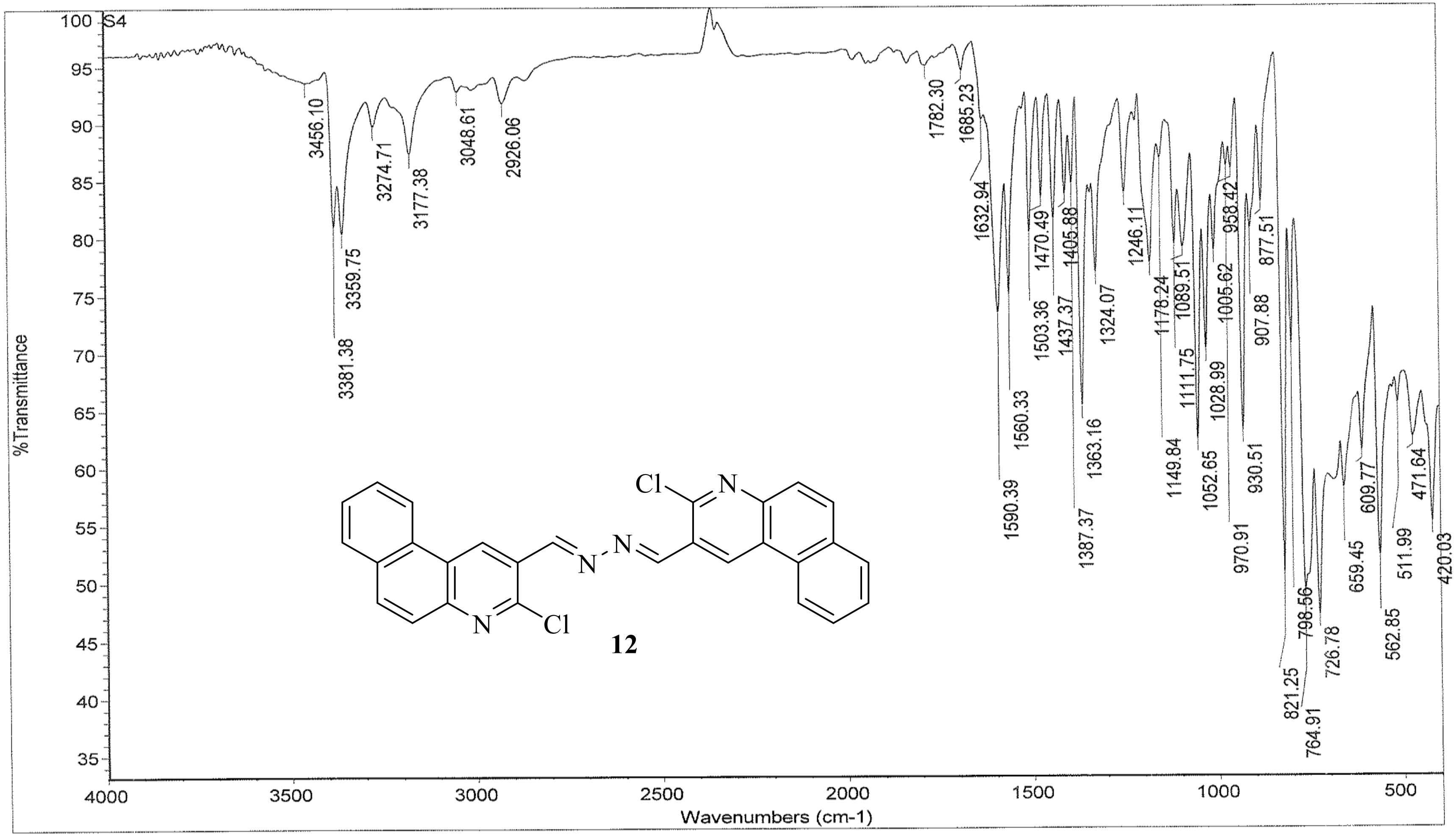
S4
%Transmittance
Wavenumbers (cm-1)
3456.10
3381.38
3359.75
3274.71
3177.38
3048.61
2926.06
1782.30
1685.23
1632.94
1590.39
1560.33
1503.36
1470.49
1437.37
1405.88
1387.37
1363.16
1324.07
1246.11
1178.24
1149.84
1111.75
1089.51
1052.65
1028.99
1005.62
970.91
958.42
930.51
907.88
877.51
821.25
798.56
764.91
726.78
659.45
609.77
562.85
511.99
471.64
420.03
Cl
N
12

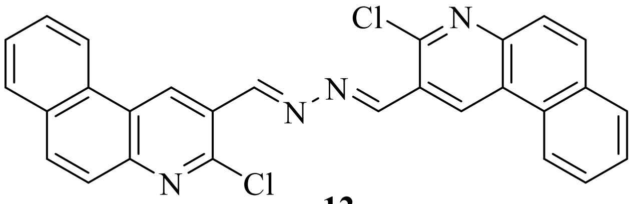

12

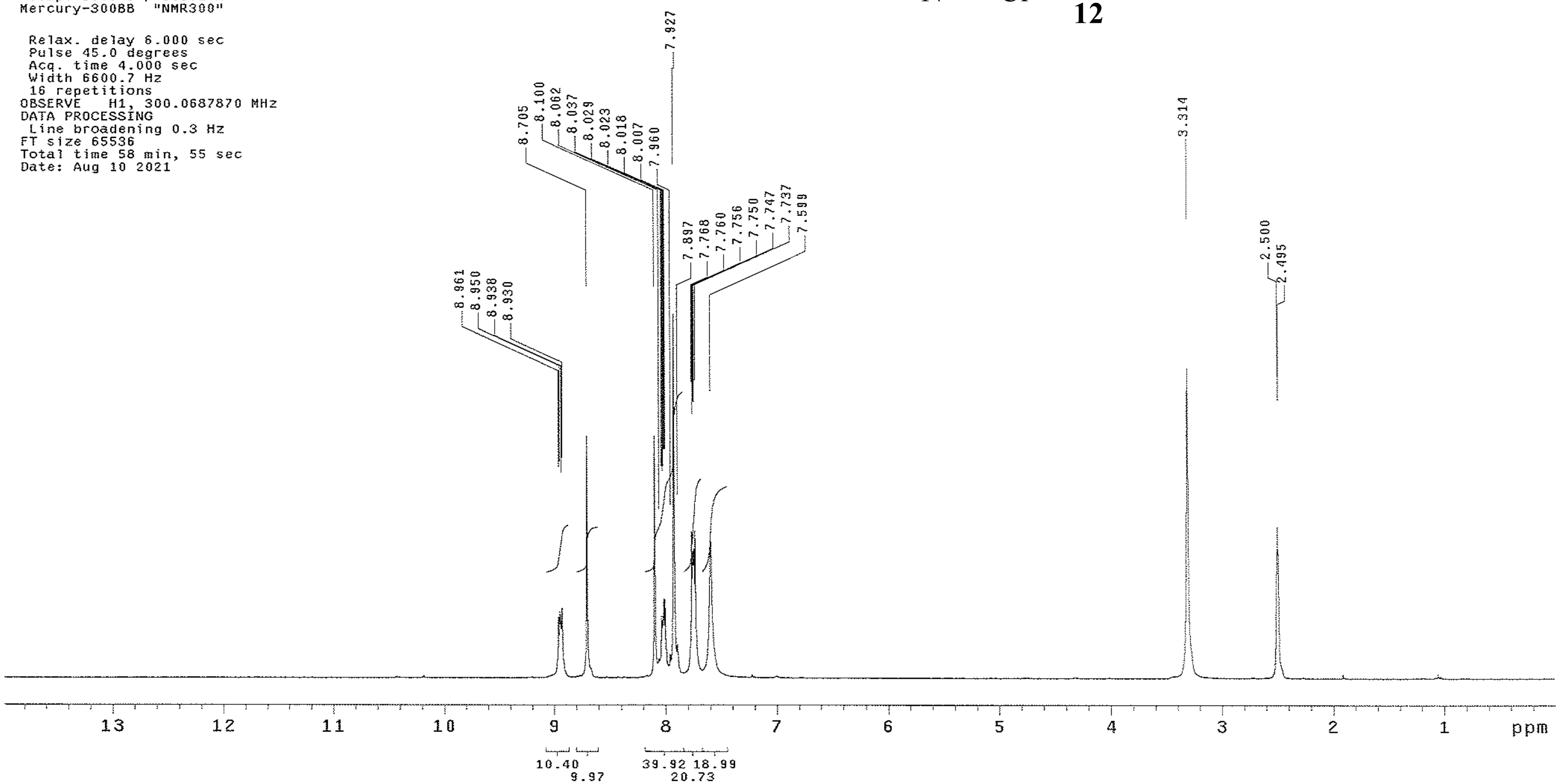
MahmoudAsran-54-DMSO-H1
Archive directory: /export/home/vnmr1/vnmrsys/data
Sample directory: DD5mm_test_12Mar2014-21:34:40
File: PROTON
Pulse Sequence: s2pul
Solvent: DMSO
Temp. 30.0 C / 303.1 K
Mercury-300BB "NMR300"
Relax. delay 6.000 sec
Pulse 45.0 degrees
Acq. time 4.000 sec
Width 6600.7 Hz
16 repetitions
OBSERVE H1, 300.0687870 MHz
DATA PROCESSING
Line broadening 0.3 Hz
FT size 65536
Total time 58 min, 55 sec
Date: Aug 10 2021
8.961
8.950
8.938
8.930
8.705
8.100
8.062
8.037
8.029
8.023
8.018
8.007
7.960
7.927
7.897
7.768
7.760
7.756
7.750
7.747
7.737
7.599
3.314
2.500
2.495
13
12
11
10
9
8
7
6
5
4
3
2
1
ppm
10.40
9.97
39.92
18.99
20.73
